# Translating transcriptomics analysis into diagnostic workflows: clinical variant identification and interpretation in hypothesis-driven and hypothesis-free approaches

**DOI:** 10.1016/j.ebiom.2026.106313

**Published:** 2026-05-28

**Authors:** Chingyiu Pang, Martin Man-Chun Chui, Wenshu Tang, Anna Ka-Yee Kwong, Hiu Yu Cherie Leung, Sze-Shing Fan, Alice Wing-Sze Kwok, Godfrey Chi-Fung Chan, Ho Ming Luk, Rosanna Ming-Sum Wong, Wanling Yang, Ivan Fai-Man Lo, Cheuk-Wing Fung, Joanna Yuet-Ling Tung, Anthony Pak-Yin Liu, Kit-San Yeung, Sheila Suet-Na Wong, Christopher Chun-Yu Mak, Brian Hon-Yin Chung

**Affiliations:** a Department of Paediatrics and Adolescent Medicine, School of Clinical Medicine, Li Ka Shing Faculty of Medicine, The University of Hong Kong, Hong Kong SAR, China; b Hong Kong Genome Institute, Hong Kong SAR, China; c Department of Paediatrics and Adolescent Medicine, Hong Kong Children’s Hospital, Hong Kong SAR, China; d Department of Clinical Genetics, Hong Kong Children’s Hospital, Hong Kong SAR, China

**Keywords:** Transcriptomics, Hypothesis-driven, Hypothesis-free, Outlier approach, ACMG variant curation, Genetic diagnosis

## Abstract

**Background:**

Despite significant advancements in genetic diagnosis, there are still bottlenecks in DNA-level testing. Challenges to diagnosis include the inability to identify the causal variant, and the lack of functional evidence leading to an accumulation of variants of uncertain significance (VUS). Recently, there has been growing evidence demonstrating the diagnostic value of RNA sequencing (RNA-seq).

**Methods:**

This diagnostic study implemented RNA-seq analysis of blood (and fibroblasts, if available) in 102 patients with genetically undiagnosed diseases. An outlier analysis for gene expression and splicing was adopted through a multi-modal machine learning algorithm (i.e., Detection of RNA Outliers Pipeline—DROP).

**Findings:**

Our analysis aided the interpretation of 22/102 (21.6%) patients through both hypothesis-driven (i.e., with a prior genetic candidate; n = 10) and hypothesis-free (i.e., without a prior genetic candidate; n = 12) approaches. Not only did this workflow aid genetic diagnosis (n = 12), but it also provided additional information to known findings (n = 4) and guided the discovery of unestablished disease mechanisms (n = 6).

**Interpretation:**

This study demonstrated the clinical and scientific value of blood transcriptomics through both hypothesis-driven and hypothesis-free approaches. We have proposed an initial framework for RNA-seq implementation into the American College of Medical Genetics and Genomics and the Association for Molecular Pathology (ACMG/AMP) guidelines using PVS1 (null variants) and PP4 (phenotypic specificity). However, other considerations are required, including further clarifications for thresholds, and detailed guidance on the incorporation of different aspects of RNA-seq results (e.g., degree of nonsense-mediated decay and completeness of splicing).

**Funding:**

This study was supported by grants from the Society for the Relief of Disabled Children, the Health and Medical Research Fund (HMRF) and Commissioned Paediatric Research at HKCH under HMRF both by the Health Bureau, The Government of the Hong Kong Special Administrative Region.

**Research in context**

**Evidence before this study**

Current sequencing technologies, including whole exome and genome sequencing, have shown a diagnostic yield capped at roughly 40%. To tackle this bottleneck, cumulative evidence has demonstrated the diagnostic value of incorporating transcriptomics into the genetic diagnostic workflow. However, a consensus has yet to be reached on when transcriptomics should be incorporated and how different aspects of the results can be considered during the variant curation process. A PubMed search was performed, using the keywords ‘(RNA-seq OR transcriptomics) AND (ES OR GS) AND (diagnosis OR adult OR child OR infant OR neonatal) NOT (carrier OR animal) in Titles and Abstracts’ for publications dated before September 2025.

**Added value of this study**

Our study aided the interpretation of genetically undiagnosed cases through implementing blood (and fibroblast) RNA-seq analysis using an outlier approach. Our findings support and reinforce the diagnostic value of RNA-seq in clinical genetics aligning with previous studies. Furthermore, we have demonstrated that blood RNA-seq is beneficial in both hypothesis-driven and hypothesis-free approaches, addressing a heated debate in the field. Through this work, we have aided not only diagnosis with currently known gene–disease associations, but also the discovery of unestablished disease mechanisms and the downstream effects of certain variants (e.g., RNA signatures). We have also proposed an initial framework for the application of RNA-seq results into the American College of Medical Genetics and Genomics and the Association for Molecular Pathology (ACMG/AMP) guidelines, where we identified areas that require further guidance.

**Implications of all the available evidence**

Given the growing clinical focus on genetic diseases and rapid advances in technologies, there is an increasing demand to tackle challenging interpretations of variants that remain unresolved at the DNA-level. Our study highlights the diagnostic value of transcriptomics and sheds light on aspects of its clinical implementation. Its applicability in a heterogeneous cohort, regardless of the presence of a prior candidate variant, supported the utility of RNA-seq in genetically unresolved cases. An initial framework has also been proposed to incorporate RNA-seq into the variant curation workflow, in which our study further highlighted considerations that are required for a more comprehensive evaluation of the results.

## Introduction

Rare diseases, individually affecting fewer than one in 2000 people by definition, have a significant burden worldwide.^1^^,^^2^ Despite individual rarity, rare diseases consist of more than 7000 types and collectively affect more than 400 million individuals globally.^2^ In Hong Kong, one in every 67 people is diagnosed with a rare disease.^3^ These patients often suffer from a long diagnostic odyssey owing to individually low disease prevalence.^1^ With an annual socio-economic cost of HK$484,253 (US$62,084) per patient on average in the Hong Kong population, the total cost and catastrophic health expenditure incidence rates increase with the duration of the diagnostic odyssey.^4^ Simultaneously, patients and caretakers shoulder a significantly lower health-related quality of life compared to counterparts with chronic diseases.^5^ These factors demonstrate the importance of tackling current hurdles in rare disease diagnosis in order to provide the most timely clinical management for patients.

A majority of rare diseases (80%) are known to have an underlying genetic cause with 70% presenting clinically in childhood,^2^ and ongoing efforts have been made to diagnose rare disease and advance genomic medicine. Clinical integration of genetic tests, exome reanalysis, newborn screening etc., have been eagerly proposed and performed in different countries. Large-scale genome projects have also been established worldwide. Despite advancements in sequencing technologies, however, whole exome sequencing (WES) only yields an overall diagnostic rate of 38%.^6^ According to our experiences, the diagnostic yield of WES ranges from 10 to 65% among different paediatric cohorts with a predominant Chinese ethnicity.^7^, ^8^, ^9^, ^10^, ^11^, ^12^, ^13^, ^14^, ^15^ Surprisingly, whole genome sequencing (WGS) was found to have a similar diagnostic yield as WES, despite higher clinical utility.^6^ Regardless of the sequencing technology, a large proportion of patients remain genetically undiagnosed owing to challenges in (1) causal variant identification due to the intrinsic property of WES selecting for coding regions, and limitations of current analytic protocols in prioritising challenging variants like non-coding variants, and (2) variant interpretation of likely causal variants that are identified but remain classified as variants of uncertain significance (VUS) due to the lack of functional evidence. This has led to an accumulation of VUS, which has been reported in 21–29% of cases remaining genetically undiagnosed after WES/WGS.^6^^,^^16^ This phenomenon is exacerbated in non-European populations owing to the limited data and studies available.^16^

While the first difficulty has been improved by multiple variant prioritisation algorithms like Exomiser^17^ and Genomiser,^18^ the issue with accumulating VUS remains a global challenge. RNA sequencing (RNA-seq) has emerged as a complementary diagnostic tool for DNA-based analysis by providing additional functional evidence. According to the latest Simple ClinVar data available (as of July 14, 2021),^19^ one-fourth of ClinVar VUS has potential RNA phenotypes. Thus, RNA-seq can guide the identification of missed causal variants from previous tests, and/or support the reclassification of VUS according to the American College of Medical Genetics and Genomics and the Association for Molecular Pathology (ACMG/AMP) guidelines.^20^^,^^21^ Several studies have shown the diagnostic utility of RNA-seq with an incremental diagnostic yield of 2.6–64.2% for unresolved ES/GS cases.^22^, ^23^, ^24^, ^25^, ^26^, ^27^, ^28^, ^29^, ^30^, ^31^, ^32^, ^33^, ^34^, ^35^, ^36^, ^37^, ^38^ The significant variability among these studies can be attributed to the differences in disease cohort, tissue samples used, analysis approach and recruitment criteria (Supplementary Table S1). RNA-seq analysis for rare disease diagnosis are typically performed using an ‘outlier’ approach, differing from the conventional differential expression approaches for case–control studies. In 2021, Yepez et al.^39^ proposed a multi-modular computational workflow–Detection of RNA Outliers Pipeline (DROP)–which investigates multiple RNA phenotypes including aberrant expression (AE) using OUTRIDER,^40^ and aberrant splicing (AS) using FRASER/FRASER2.^41^^,^^42^

Owing to the intrinsic differences between genomics and transcriptomics, the clinical implementation of RNA-seq is met with challenges in accessing clinically relevant tissues for analysis. As one of the factors for the effectiveness of RNA-seq, efforts have been made in the field to identify favourable clinically accessible tissues (CATs) as functional proxies for clinically relevant but inaccessible tissues like the brain. Fibroblasts and blood are common CATs being considered owing to their accessibility. Despite fibroblasts having a higher coverage of disease-causing genes,^26^^,^^35^^,^^43^ blood has demonstrated a coverage of 56.8–86.7% in different disease-specific gene panels^43^ while being the least invasive CAT. Blood RNA-seq alone has achieved an 8.5% incremental diagnostic yield,^24^^,^^28^^,^^29^^,^^36^^,^^38^^,^^44^ (Supplementary Table S1) showing that blood can serve as a readily available CAT with a promising diagnostic utility.

Growing interest lies in the clinical application of transcriptomics in the genetic diagnostic workflow. Recently, Zhao S. et al.^43^ suggested a clinical validation framework throughout the entire RNA-seq workflow for benchmarking between different laboratories. However, the direct application of RNA-seq results into the variant curation pipeline has yet to be investigated, apart from the PVS1 decision tree proposed by the ClinGen splice variant working group.^45^ In our study, we recruited 102 patients that remained genetically undiagnosed after routine genetic testing and/or WES. RNA was extracted from blood (and fibroblast tissues; n = 11) and subjected to RNA-seq analysis using the DROP pipeline to detect aberrant RNA phenotypes in expression and splicing. By using hypothesis-driven (i.e., with a prior genetic candidate) and hypothesis-free (i.e., without a prior genetic candidate) approaches, this study aims to shed light on the value of RNA-seq in genetic diagnosis, aspects of its clinical implementation and more importantly, considerations required when incorporating transcriptomics interpretation into the current ACMG/AMP guidelines.

## Methods

### Patient recruitment

Patients with congenital disorders who remained genetically undiagnosed after routine genetic testing/WES (n = 102) were recruited from two hospitals affiliated with The University of Hong Kong (HKU): Queen Mary Hospital and Hong Kong Children’s Hospital during the period of 2021–2024. Clinical information including sex, age and clinical phenotypes was retrieved from patients’ clinical records. Our study recruitment ensured inclusivity and sufficient sample size, resulting in a heterogeneous cohort. The sample sizes recommended for the applied analytical algorithms are a minimum of 50–60 and 30 samples for OUTRIDER^40^ and FRASER2.0,^41^ respectively, to ensure sufficient power.^39^

### Ethics

The study was approved by the institutional review boards of the University of Hong Kong/Hospital Authority Hong Kong West Cluster and Hong Kong Children’s Hospital (UW 12–211 and HKCH-REC-2020-035) and conformed to the Declaration of Helsinki. Written informed consent was obtained from all participants and/or their guardians.

### Sample preparation and RNA-seq

Peripheral blood collected from patients was treated with Red Blood Cell Lysis Solution (Qiagen). Parental samples were also collected if possible after clinical evaluation. Among the 102 patients, fibroblast samples were also available from 11 participants. Fibroblasts were cultured into a monolayer at 80% confluency in high-glucose Dulbecco’s Modified Eagle Medium (DMEM; Gibco) supplemented with 10% foetal bovine serum and 1% penicillin/streptomycin (Thermo Fisher Scientific).

The subsequent RNA extraction and RNA-seq processes were similar to those in our previous studies.^46^^,^^47^ In brief, blood and fibroblast samples were subjected to further lysis and RNA extraction using TRIzol™ Reagent (Thermo Fisher Scientific). Only extracted RNA with RNA integrity (RIN) ≥ 7 was used for RNA-seq at the Centre for PanorOmic Sciences (CPOS) of HKU. Library preparation through poly-A-tail capture was performed using the KAPA mRNA HyperPrep Kit (Roche Diagnostics) according to the manufacturer’s protocol. RNA-seq was performed in paired-end runs (151 bp) on an Illumina NovaSeq 6000 platform (RRID: SCR_016387) with a sequencing depth of at least 100 million reads to achieve higher sensitivity for outlier detection, while balancing the associated decrease in specificity.^39^^,^^40^^,^^42^^,^^43^ Only samples with 90% of sequenced bases reaching a Phred Score of 30 (99% base accuracy) were included.

### RNA-seq data processing and analysis

RNA-seq data were subjected to data cleaning by AfterQC (v0.9.6; RRID: SCR_016390) before being mapped to the GRCh37 human reference genome, and output as BAM files using STAR (v2.5.2a; RRID: SCR_004463) in two-pass mode. The sequencing data from different tissues were processed independently using the computational module workflow DROP. It is a protocol that automates RNA-seq analysis using an outlier approach from raw input files, allowing a standardised workflow with multiple modules.^39^ In our study, DROP v1.3.3 was utilised to detect AE outliers using OUTRIDER,^40^ and AS outliers using FRASER2.0 (v1.99.3).^41^ Denoising autoencoders to control for confounders not known *a priori* were integrated in the modules, including but not limited to technical confounders like sequencing batches and biological confounders like sex and age. This outlier approach is substantially different from a conventional differential expression analysis, where we compare expression levels between cases and controls. Here, we compare individual target samples with the rest of the cohort, on the basis of the rarity of two unrelated individuals having the same disease-causing variant leading to identical RNA phenotypes.^39^ As suggested by the DROP developers,^39^ publicly available RNA-seq datasets from The Genotype-Tissue Expression (GTEx) project (dbGaP accession number phs000424.v8.p2; RRID: SCR_013042)^48^ were included as controls to increase statistical power (sensitivity) by increasing sample size along with our internal controls; the details of GTEx controls are provided in Supplementary Table S2.

To increase the sensitivity of detecting expression outliers, we defined significant AE outliers at a false discovery rate (FDR) ≤ 0.1, which is slightly looser than the default threshold (FDR ≤ 0.05) used in other studies.^26^^,^^35^^,^^39^ Significant AS outliers were filtered based on the default thresholds (FDR ≤ 0.1 and effect size |ΔJ| ≥ 0.1).^41^ |ΔJ| (J = Intron Jaccard Index) represents the absolute value of differences between the expected (modelled by an autoencoder under the hypothesis that all individuals in the cohort have the same splicing specificity and efficiency), and the observed splicing specificity and efficiency of a particular splice site of an intron.^41^ The FDR for p-values derived from both modules (AE: negative-binomial distribution; AS: beta-binomial distribution) were generated using the Benjamini-Yekutieli method to correct for multiple testing.^40^, ^41^, ^42^ In this study, p-value correction was performed for 16,946 genes per sample (OUTRIDER) and 206,605 junctions per sample (FRASER2.0). Manual inspection of sequencing reads using the Integrative Genomics Viewer (IGV; RRID:SCR_011793) was performed in cases with candidate VUS and significant aberrant RNA outliers. In perfect scenarios, fold changes of 0.5 and 0 from AE, and |ΔJ| of 0.5 and 1 from AS would indicate heterozygous and homozygous events respectively. However, these values are highly subjected to variability owing to transcriptomics processes being dynamic and affected by different factors such as nonsense-mediated decay (NMD) efficiency, and therefore their interpretation was made along with manual inspection of IGV in case-specific scenarios.

Aberrant RNA outliers from both modules with candidate VUS and/or reported gene–disease associations in the Online Mendelian Inheritance in Man (OMIM; RRID:SCR_006437) database,^49^ specifically those that phenotypically matched with patient phenotypes, were prioritised for manual inspection. Potential candidate non-morbid genes or aberrant results (e.g., large numbers of outliers) also prompted deeper investigation if highly suspicious, especially those that might indicate the presence of an RNA signature (i.e., a specific or non-specific group of aberrant events caused by the defective gene). Causative variants were searched for using existing DNA/RNA-seq data. When causative variants remained unidentifiable in specific cases, short-read WGS (srWGS) and long-read WGS (lrWGS) were performed according to an optimised protocol as previously described.^50^ In brief, for srWGS, following genomic DNA extraction, polymerase chain reaction (PCR)-free srWGS libraries were prepared using the KAPA HyperPlus Kit (Kapa Biosystems Inc.) and sequenced on an Illumina NovaSeq 6000 (Illumina Inc.; RRID:SCR_016387) or NovaSeq X Plus sequencer (Illumina Inc.; RRID:SCR_024568). For lrWGS, the processes were performed following the instructions of the manufacturer, Oxford Nanopore Technologies (ONT; RRID:SCR_003756). Using 1 μg of extracted genomic DNA, libraries were prepared with the ONT Ligation Sequencing Kit (SQK-LSK114) with sequencing performed for up to 72 h on a PromethION P-24 device using an R10.4.1 flow cell (ONT, Oxford UK; RRID:SCR_017987). The generated srWGS and lrWGS data were then subjected to quality control, secondary analysis (including alignment to the GRCh38/hg38 human reference genome), and tertiary analysis. A wide range of variant types were investigated including single nucleotide variants, small insertion/deletion variants, copy number variants, large structural variants, short tandem repeat expansions, as well as methylation changes (only for lrWGS).

Manual curation of identified variants was then performed according to the ACMG/AMP guidelines.^20^^,^^21^^,^^45^^,^^51^ Sanger sequencing or real-time PCR was done in cases where variant confirmation and segregation analysis were required. The DNA and RNA datasets generated will not be made publicly available due to ethical restrictions regarding minor participants and patient anonymity. Results were returned to clinicians for further clinical follow-up.

### Statistics

In cases with an aberrant number of outliers, statistical significance was evaluated as follows: With the large variety of overexpressed genes attributed to the heterogeneity of our cohort, mean and standard deviation of the number of outliers from the control dataset (unrelated samples) were compared with the patient data for the derivation of Z-scores. Student’s T distribution with a degree of freedom of 122 was then used to derive two-tailed p-values from Z-scores. The p-value significance threshold was taken as 0.05.^52^

Other statistical tests performed in DROP are in-built executions of the pipeline.^39^, ^40^, ^41^, ^42^

### Role of the funding source

The funding sources are detailed in the Acknowledgement section. The funders had no role in the design and conduct of this study; collection, management, analysis and interpretation of the data; preparation, review, or approval of the manuscript; or the decision to submit the manuscript for publication. All authors had full access to the data in the study and accept responsibility for the decision to submit for publication.

## Results

### Cohort characteristics

A total of 102 probands were recruited for blood transcriptomics analysis with most cases analysed as singletons (48.0%) or trios (41.2%). While the current study did not identify explicit advantages of trio analysis, further studies are required to investigate the implementation of parental data to enhance the analysis. The mean age of the cohort was 9.6 years (standard deviation of 6.6), with a majority of probands aged 0–18 years old (89.2%). The remaining 11 cases were >18 years of age, having been recruited after being referred to the adult clinic with minimum follow-up on their genetic condition. Our cohort comprises a heterogeneous range of genetic conditions, with 57.8% of patients presenting with conditions associated with the central nervous system, followed by cardiac diseases and suspected mitochondrial diseases, among others. Further details of the cohort are provided in Table 1, Supplementary Information S1–S3 and Supplementary Tables S3 and S4.

**Table 1:** Cohort demographics.

Total	n = 102	
Sex		
Female	46	45.1%
Male	56	54.9%
Analysis		
Singleton	49	48.0%
Duos	10	9.8%
Trio	42	41.2%
Quadro	1	1.0%
Age		
Mean (SD)	9.6 years (6.6)	
≤18 years old	91	89.2%
>18 years old	11	10.8%
Disease categories		
Central nervous system^a^	59	57.8%
Suspected mitochondrial diseases	8	7.8%
Cardiac diseases	9	8.8%
Congenital anomalies	2	2.0%
Metabolic disorders	6	5.9%
Endocrine disorders	3	2.9%
Immunodysregulatory disorders	2	2.0%
Others^b^	13	12.7%

Sex-disaggregated demographics are provided in Supplementary Table S4.

a Includes neurodevelopmental disorder, neurodegenerative disorder, epilepsy, neurotransmitter disease, movement disorder and neurological immune disorder.

b Conditions associated with other systems or those that cannot be classified under other disease categories.

### Hypothesis-driven blood RNA-seq analysis

In our cohort, hypothesis-driven analysis aided the interpretation of ten cases (9.8%), in which prior VUS candidates were identified from previous genetic tests. Among these cases, six achieved genetic diagnoses with RNA-seq providing further supporting evidence to upgrade the previously identified VUS to a likely pathogenic/pathogenic (LP/P) variant according to the ACMG/AMP guidelines (Table 2; Supplementary Table S3; Supplementary Information S1).^20^^,^^45^^,^^51^ All upgraded variants were intronic variants outside the ± 1,2 dinucleotide canonical splice positions, where PVS1 criteria (i.e., “Null variant (nonsense, frameshift, canonical ± 1 or 2 splice sites, initiation codon, single or multi-exon deletion) in a gene where loss-of-function (LoF) is a known mechanism of disease”)^20^ or PS3 criteria under gene specific guidelines (i.e., case B29) can only be applied if we have functional data supporting pathogenicity through RNA-related findings.^45^

**Table 2:** Cases aided by hypothesis-driven blood RNA-seq analysis (n = 10).

Cases with new diagnosis (n = 6)						
Case No.	Indication and phenotypes	Previously identified VUS	Gene-disease association	RNA-seq finding	Original ACMG classification (before RNA-seq)	New ACMG classification (after RNA-seq)
B10^53^ (Trio)	Cardiac disease: dilated cardiomyopathy with neutropenia and DD	Maternally inherited hemizygous *TAFAZZIN* variant NM_000116.5: c.284+5G>A	Barth Syndrome (OMIM# 302060)	**AS:***TAFAZZIN* (|*Δ*J| = 0.5; FDR = 0.0000079)	**VUS** (PM2_supporting, PP3)	**Likely pathogenic** (PVS1_Strong (RNA), PM2_supporting, PP4_strong)
B11^53^ (Singleton)	Cardiac disease: hypertrophic cardiomyopathy, cardiac structural anomalies, DD and facial features of Noonan Syndrome	Compound heterozygous *LZTR1* variants: 1) Maternally inherited NM_006767.4: c.1261-3C>G and 2) Paternally inherited c.1943-256C>T	Noonan syndrome 2 (OMIM# 605275)	**AS:***LZTR1* (|*Δ*J| = 0.63; FDR = 0.00095) *LZTR1* (|*Δ*J| = 0.38; FDR = 0.032)	c.1261-3C>G **VUS** (PM2_supporting, PM3, PP3) c.1943-256C>T **Likely Pathogenic** (PM2_supporting, PM3, PVS1 (RNA))	c.1261-3C>G **Likely Pathogenic** (PM2_supporting, PM3, PVS1_strong (RNA)) c.1943-256C>T **Likely Pathogenic** (PM2_supporting, PM3, PVS1 (RNA))
B29 (Trio)	Neurodevelopmental disorder: macrocephaly, history of global DD and dysmorphic features	*De novo* heterozygous *PTEN* variant NM000314.8: c.209+3A>T	Cowden syndrome 1 (OMIM# 158350)	**AS:***PTEN* (|*Δ*J| = 0.55; FDR = 2.7 × 10^−11^)	**VUS** (PM2_supporting, PS2_moderate, PP3)	**Likely Pathogenic** (PM2_supporting, PS2_moderate, PS3_strong)
B56^a^ (Singleton)	Suspected mitochondrial disease: multiple motor phenotypes, global DD, and epilepsy	Compound heterozygous *RARS2* variants: 1) Maternally inherited NM_020320.5: c.1238-28T>G and 2) Paternally inherited c.685C>T p.(Arg229∗)	Pontocerebellar hypoplasia type 6 (OMIM# 611523)	**AS:***RARS2* (FDR = 0.31; unadjusted p-value = 0.000016; |*Δ*J| = 0.46)	c.1238-28T>G **VUS** (PM2_supporting, PM3, PP3) c.685C>T **Pathogenic** (PVS1, PM2_supporting, PM3)	c.1238-28T>G **Likely Pathogenic** (PVS1_strong(RNA), PM2_supporting, PM3) c.685C>T **Likely Pathogenic** (PVS1_strong(RNA), PM2_supporting, PM3)
B68 (Trio)	Neurodevelopmental disorder: bilateral hearing loss, hydronephrosis, spastic diplegia and history of global DD, ID	*De novo* hemizygous *MSL3* variant NM_078629.4: c.749+5G>A	Basilicata-Akhtar syndrome (OMIM# 301032)	**AS:***MSL3* (|*Δ*J| = 0.95; FDR = 7.9 × 10^−24^)	**VUS** (PP3, PS2_supporting, PM2_supporting)	**Likely pathogenic** (PVS1_strong(RNA), PS2_supporting, PM2_supporting)
B119 (Singleton)	Neurodevelopmental disorder: microcephaly, failure to thrive, global DD, ID, autism and bilateral hearing loss	*De novo* heterozygous *NIPBL* variant NM_133433.4:c.64+5G>C	Cornelia de Lange syndrome 1 (OMIM# 122470)	**AS:***NIPBL* (|*Δ*J| = 0.22; FDR = 0.0055)	**VUS** (PP3, PS2_supporting PM2_supporting)	**Likely Pathogenic** (PVS1_moderate (RNA), PS2_strong, PM2_supporting, PP4_Moderate)

Cases where RNA-seq provided further information for interpretation (n = 4)			
Case no.	Indication	RNA-seq finding	Information provided by RNA-seq
B12 (Trio)	Congenital Anomalies: type 1 jejunal atresia and inguinal hernia, also involving DD, genital, and ocular phenotypes.	**AS:***SON* (|*Δ*J| = 0.45; FDR = 0.00065)	***Refined in silico prediction and indicate limited damaging effects of variant*** • Variant: NM_138927.4 (*SON*): c.78-2A>C (paternally inherited heterozygous VUS) • RNA-seq: in-frame 6 bp deletion • Interpretation: Limited damaging effect
B37 (Trio)	Neurodevelopmental disorder: global DD with mild ID and indications of Dandy-Walker variant or vermian-cerebellar hypoplasia	**AE:** 14 genes on chromosomal Xq28 were detected as significant overexpression (FDR < 0.1; FC > 2).	***Confirming duplication and narrow down possible genes*** • Variant: Duplication on chromosomal Xq28 (*de novo* heterozygous) • RNA-seq: confirmed duplication and suggested *GDI1, RPL10, FAM50A, LAGE3* associated with X-linked intellectual developmental disorder as candidate genes underlying patient phenotype • Interpretation: *GDI1* on patient phenotypes are highly suspected^54^
B48^53^ (Singleton)	Cardiac disease: Dilated cardiomyopathy	*MYBPC3* is not detected as RNA splicing outlier	***Validate in silico prediction and refined prior experimental results*** • Variant: NM_000256.3 (*MYBPC3*):c.1224-80G>A (paternally inherited heterozygous VUS) • RNA-seq: exon 14 elongation (78 bp) • Interpretation: RNA-seq supported SpliceAI prediction^70^ and provided a more refined picture to the minigene splice report assay^81^ but did not change the classification of VUS
B80 (Duos)	Neurodevelopmental disorder: severe global DD with ID, failure to thrive, hypotonia, Dandy-walker malformation, and facial dysmorphism	**AS:***KMT2A* (|*Δ*J| = 0.47; FDR = 0.00097)	***Validated in silico prediction and indicate limited damaging effects of variant*** • Variant: NM_001197104.2 (*KMT2A*): c.4480–18_4480del (heterozygous, not maternally inherited) • RNA-seq: deletion of 3 bp in exon 12 without frameshift • Interpretation: limited damaging effect

Further description of individual cases is available in Supplementary Information S1. Thresholds for AE: FDR ≤ 0.1. Thresholds for AS: FDR ≤ 0.1 and |ΔJ| ≥ 0.1. ACMG: American College of Medical Genetics; AE = Aberrant Expression; AS = Aberrant Splicing; DD = developmental delay; FC = fold change; FDR = false discovery rate; ID = intellectual disability; OMIM= Online Mendelian Inheritance in Man; RNA-seq: RNA sequencing; VUS = Variants of uncertain significance; |*Δ*J| = difference in Intron Jaccard Index.

a This case was diagnosed through manual inspection of Integrative Genomics Viewer with insignificant RNA sequencing (RNA-seq) results based on thresholds set in this study.

**Fig. 1:**
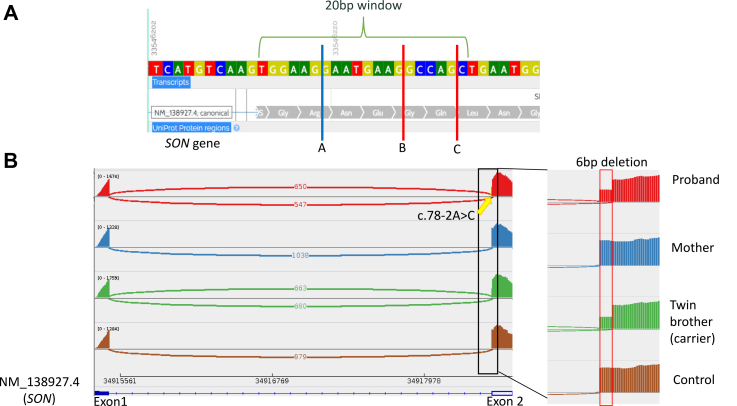
**In silico prediction and RNA-seq data of case B12.** (A) Three cryptic splice sites were identified within ± 20 bp window from the affected splice site in exon 2 of *SON (NM_138927)*. Blue line indicates in-frame deletion while red line indicates a frameshift. (B) The Integrative Genomics Viewer showed a clear usage of cryptic splice site in almost half of the transcripts in both proband and carrier twin brother who is not affected, which is absent in non-carrier mother and control. A zoom-in of the splice event showed a deletion of 6 bp in both the proband and the carrier twin brother.

In the remaining four cases, RNA-seq clarified uncertain findings from the DNA-level investigation (Table 2; Supplementary Table S3; Supplementary Information S1). In one case (B37), RNA-seq pinpointed candidate underlying genes within a large duplication region. Overexpression of 14 genes on chromosomal Xq28 was detected, aligning with the chromosomal microarray finding of *de novo* duplication. Four candidates were flagged to possibly explain the patient’s phenotype: *RPL10, FAM50A, LAGE3* and specifically *GDI1* supported by previously reported patients with recurrent but variable 0.3 Mb copy-number gain at Xq28.^54^ In three other cases (B12, B48,^53^ B80), RNA-seq refined or validated *in silico* predictions, refined prior experimental results (B48^53^) and suggested limited damaging effects of variants in two cases (B12, B80). This demonstrated that the diagnostic value of RNA-seq lies not only in upgrading variants but also in re-evaluating variants for potential downgrading or exclusion in subsequent analyses. This was exemplified with case B12, where the proband was experiencing gross motor delay, verbal expression delay, brain and genitalia abnormalities and multiple ocular phenotypes. A paternally inherited heterozygous variant NM_138927.4(*SON*): c.78-2A>C was identified in a prior genetic test. This variant was originally classified as a VUS owing to the presence of three potential cryptic splice sites in the ±20 bp window based on *in silico* predictions thereby leading to ambiguous interpretation (Fig. 1A). One cryptic splice site (A) leads to in-frame splicing while the other two (B and C) lead to frameshift, which are much more deleterious than the former. However, based solely on DNA-level data, no explicit conclusions could be drawn on the variant’s pathogenicity without knowing the preferred cryptic splice site. In our RNA-seq results, *SON* was detected as an AS outlier (|*Δ*J| = 0.45; FDR = 0.00065), showing that cryptic splice site A was used in almost half of the detected transcripts leading to an in-frame deletion of 6 bp without utilising other cryptic splice sites (B and C) (Fig. 1B). The insignificant expression results (fold change = 1.1; unadjusted p-value = 0.28; FDR = 1), further suggested that NMD was unlikely to be occurring at the other cryptic splice sites (B and C). This supported that the pathogenicity of this variant is limited as no frameshift events were observed, and the in-frame deletion is not situated within a known functional domain. Often overlooked during analysis, downgrading a variant’s pathogenicity is just as important as upgrading a variant to establish a genetic diagnosis.

### Hypothesis-free blood RNA-seq analysis

The interpretation of another 11 cases (10.8%) was aided by hypothesis-free blood RNA-seq analysis, where diagnostic discoveries were made in the absence of guidance from prior genetic tests. On average, 8.6 (AE) and 12.5 (AS) outliers per sample were filtered down to 2.0 (AE) and 2.9 (AS) outliers per sample after selecting for morbid genes, resulting in a handful of candidate genes for further interpretation (Supplementary Table S5). Five of these cases achieved diagnoses through the identification of previously undetected causative P/LP variants of currently known disease genes, including intronic variants and deletion mutations (Table 3; Supplementary Table S3; Supplementary Information S2). RNA-seq guided variant identification in DNA-level tests, where variants were identified directly from RNA data in two cases (B47 and B107), and provided functional evidence for variant pathogenicity classification.

**Table 3:** Cases aided by hypothesis-free blood RNA-seq analysis (n = 11).

Cases with new diagnosis (n = 5)						
Case no.	Indication	RNA-seq Finding	Gene-disease association	Variant identified	ACMG classification (after RNA-seq)	Role of RNA-seq in diagnosis
BF4 (Trio)	Suspected mitochondrial disease: Complex I and IV deficiency mitochondrial disease	**AE:***GFM1* (FC = 0.57; FDR = 0.016) *MFSD1* (FC = 0.03; FDR = 1.1 × 10^−10^) Results later confirmed with fibroblast samples^47^	Combined oxidative phosphorylation deficiency 1 (OMIM# 609060)	Biparentally inherited homozygous 104.5 kb deletion (chr3:158435847-158540316) (GRCh37)	**Likely Pathogenic** (PP4_strong, PM3_supporting, PM2_supporting)	• Guided variant identification in ES, further confirmed in GS. • Provided evidence for PP4_strong.
B47 (Trio)	Neurodevelopmental disorder: global DD with ID, autism, and central hypotonia	**AS:***KMT5B* (|*Δ*J| = 0.44; FDR = 0.057)	Intellectual developmental disorder 51 (MRD51; OMIM# 617788)	Maternally inherited heterozygous *KMT5B* variant NM_017635.5:c.977+2T>A	**Likely Pathogenic** (PVS1_strong (RNA), PM2_supporting, PP4_supporting)	• Variant identified in RNA data, confirmed with sanger sequencing • Provided evidence for PVS1_strong(RNA) • Prompted further investigation (e.g., DNA methylation signature)
B84 (Singleton)	Neurodevelopmental disorder: suspected distal myopathy, microcephaly, speech delay, learning disability and ADHD	**AE:***CTCF* (FC = 0.66; FDR = 0.000021)	Intellectual developmental disorder, autosomal dominant 21 (OMIM# 615502)	*De novo* heterozygous ∼280 kb deletion [NC_000016.10:g.(67429006_67710074)del]	**Pathogenic** (1 point for CNV loss)	• Guided variant identification in GS.
B105 (Trio)	Multisystem disorder: Neurofibromatosis with distinctive features.	**AS:***NF1* (|*Δ*J| = 0.32; FDR = 0.021)	Neurofibromatosis type 1 (OMIM# 162200)	*De novo* heterozygous *NF1* deletion NC_000017.11:g. 31327741_31329914delinsT (NM_001042492.3:c.5511_5610-382delinsT)	**Pathogenic** (PVS1_moderate (RNA), PS2, PM2_supporting, PP4_strong)	• Guided variant identification in GS • Provided evidence for PVS1_moderate (RNA)
B107 (Trio)	Multisystem disorder: Neurofibromatosis with distinctive features	**AS:***NF1* (|*Δ*J| = 0.56; FDR = 0.0046)	Neurofibromatosis type 1 (OMIM# 162200)	*De novo* heterozygous *NF1* variant NM_001042492.3:c.2410-16A>G	**Pathogenic** (PVS1_strong (RNA), PM2_supporting, PP4_strong, PS2)	• Variant identified in RNA data, confirmed in GS • Provided evidence for PVS1_strong(RNA)

Cases with disease mechanism discovery (n = 6)			
Case no.	Indication	RNA-seq finding	Information provided by RNA-seq
B22^56^ (Trio)	Neurodegenerative disorder: DD and progressive motor phenotypes.	**AE:***PSMF1* (FC = 0.23; FDR = 0.020) **AS:***PSMF1* (|*Δ*J| = 0.44; FDR = 8.3 × 10^−24^)	***Discovered potential new disease gene*** • RNA-seq: 17 bp exon 2 elongation (majority) leading to NMD • Variant: NM_006814.4 (*PSMF1*):c.282+5G>A (biparentally inherited homozygous) • Investigation: RNA-seq flagged *PSMF1* as possible disease gene, subsequently aiding new gene–disease association through international collaboration
B31^52^ (Trio)	Neurodevelopmental disorder: global DD with severe ID and hypotonia, short stature and dysmorphic features	**AS:** Significant aberrant splice events in 106 genes (FDR<0.1), which is significantly larger (Z-score = 4.8, Student T two-tailed p-value = 4.7 × 10^−6^) than other internal controls and external GTEx controls—RNA signature	***Discovered potential new disease gene*** • RNA-seq: RNA signature • Variant: NM_004640.7 (*DDX39B*): c.368G>A p.(Arg123Gln) (*de novo* heterozygous) • Investigation: RNA-seq flagged *DDX39B* as possible disease gene, leading to new gene–disease association through international collaboration and establishment of RNA signature verifying gene function.
B66^52^ (Singleton)	Neurodevelopmental disorder: global DD with hypotonia, seizure, autistic behaviour, short stature and dysmorphic features.	**AS:** Significant aberrant splice events in 303 genes with FDR<0.1 which is significantly larger (Z-score = 14.9, Student T two-tailed p-value = 2.4 × 10^−29^) than other control and external GTEx controls—RNA signature	***Validating functionality of newly discovered disease-genes*** • RNA-seq: RNA signature • Variant: NM_004640.7 (*DDX39B*): c.109G>T p.(Gly37Cys) (*de novo* heterozygous) • Investigation: RNA signature helped verify the function of *DDX39B*
B67^52^ (Duos)	Neurodevelopmental disorder: delayed motor development, history of seizures, autism and short stature.	**AS:** Signature aberrant splice events in 297 genes with FDR<0.1 which is significantly larger (Z-score = 14.6, Student T two-tailed p-value = 1.2 × 10^−28^) than other control and external GTEx controls—RNA signature **AS:***DDX39B* (| *Δ*J| = 0.48; FDR = 1.8 × 10^−10^)	***Validating functionality of newly discovered disease-genes*** • RNA-seq: RNA signature and 33 bp in-frame deletion in exon 5 of *DDX39B* • Variant: NM_004640.7 (*DDX39B*): c.433-1G>T (maternally inherited heterozygous) • Investigation: RNA signature helped verify the function of *DDX39B.* Aberrant splice event supported the pathogenicity of the variant.
B93 (Trio)	Neurodevelopmental disorder: global DD and autism	**AE:***HECTD4* (FC = 0.51; FDR = 0.013)	***Suggested possible new disease mechanism*** • RNA-seq: *HECTD4* downregulation by half • Variant: GCC repeat expansion of >1000 bp in *HECTD4* 5’ UTR (heterozygous) and is hypermethylated • Investigation: possible new disease mechanism where milder form of the associated AR neurodevelopmental disorder is manifested in monoallelic form through methylation-related events
B114 (Singleton)	Multisystem disorder: moderate ID, bilateral sensorineural hearing loss, and genitalia abnormalities.	**AE:***WDR11* (FC = 0.55; FDR = 0.0014) 26 genes (including *WDR11*) within the chr10 deletion region are under expressed (FDR<0.1; FC = 0.32–0.61)	***Suggested possible expansion of disease spectrum*** • RNA-seq: downregulation of *WDR1*1 by half • Variant: NC_000010.11:g.(119969782_133655950) del (heterozygous) • Interpretation: Under expression of 26 genes (including *WDR11*) with this region supported the deletion. Patient phenotype lies between the established *WDR11*-associated AD and AR disease, suggesting a spectrum of disease associated with this gene.

Further description of individual cases is available in Supplementary Information S2. Thresholds for AE: FDR ≤ 0.1. Thresholds for AS: FDR ≤ 0.1 and |ΔJ| ≥ 0.1. ACMG = American College of Medical Genetics; AD = Autosomal dominant; ADHD = Attention deficit hyperactivity disorder; AE = Aberrant Expression; AR = autosomal recessive; AS = Aberrant Splicing; CNV = copy number variant; DD = developmental delay; ES = exome sequencing; FC= Fold change; FDR = false discovery rate; GS = genome sequencing; GTEx = Genotype-Tissue Expression Project; ID = intellectual disability; NMD = nonsense mediated decay; OMIM= Online Mendelian Inheritance in Man; RNA-seq: RNA sequencing; UTR = untranslated region; |*Δ*J| = difference in Intron Jaccard Index.

In the remaining six cases, RNA-seq guided the discovery of unestablished disease mechanism by prioritising relevant genes for further functional studies through (1) discovering new disease genes; (2) widening the variant type by suggesting tandem repeat variants in a disease that only had single nucleotide variants reported; and (3) broadening the phenotypic spectrum of existing diseases (Table 3; Supplementary Table S3; Supplementary Information S2).

Regarding the discovery of new disease genes, RNA-seq guided and supported the establishment of two new disease genes. Our RNA-seq analysis directly detected a new disease gene as an outlier in case B22, who was suspected to have a mitochondrial or neurodegenerative disease. We detected *PSMF1* with both aberrant expression (Fold change (FC) = 0.23; FDR = 0.020) and aberrant splicing (|*Δ*J| = 0.44; FDR = 8.3 × 10^−24^) in this proband which guided the identification of biparentally inherited NM_006814.4 (*PSMF1*):c.282+5G>A in both RNA-seq and exome data. The role of *PSMF1* in proteasome transport in axons^55^ sparked interest in the possibility of this gene being associated with neurological disorders. This subsequently led to further collaboration with an international research team for the establishment of disease associations between *PSMF1* and a neurodegenerative syndrome with movement disorders.^56^ The downregulation of this gene from our RNA-seq results is suggestive of a LoF mechanism for this gene–disease association. In a separate proband, B31, who also presented with neurodevelopmental phenotypes, RNA-seq indirectly detected the downstream effect (e.g., RNA signature) of the aberrant causal gene. A significantly larger number (n = 106) of aberrant splicing outliers were detected in this case compared to controls (Z-score = 4.8, Student T two-tailed p = 4.7 × 10^−6^). The *de novo* variant NM_004640.7 (*DDX39B*): c.368G>A was subsequently identified in exome data guided by the RNA-seq finding. *DDX39B* is a key member of the TREX-complex which is important for mRNA transcription and export processes.^57^ Further collaboration with an international research team identified five additional individuals with variants in this gene sharing similar phenotypes establishing its association with TREX-complex–related neurodevelopmental syndrome.^52^ Additionally, a consistent RNA signature (i.e., large number of non-specific aberrant splice events) was detected in affected individuals, B66 and B67 who share neurodevelopmental disorder phenotypes, which might serve as future diagnostic candidates.

In some complex cases, the detection of an aberrant expression outlier by RNA-seq can serve as a catalyst, guiding subsequent advanced genomic testing to uncover previously unrecognised variant types. Demonstrating this, we identified *HECTD4* as an aberrant expression outlier (FC = 0.51; FDR = 0.013) in proband B93, who presented with global developmental delay and autism spectrum disorder (ASD). This aberrant expression prompted subsequent lrWGS analysis revealing a heterozygous GCC expansion of >1000 bp in the 5’ UTR of *HECTD4*, accompanied by simultaneous hypermethylation on the same allele. No other likely variants were identified in the gene. *HECTD4* is previously associated with “Neurodevelopmental disorder with seizures, spasticity, and complete or partial agenesis of the corpus callosum” (MIM #620250) in an autosomal recessive form. However, our patient’s phenotypes were found to be less severe than the previous (and limited) reported cases with LoF and missense variants.^58^ The SFARI Gene database, which evaluates the strength of association between specific genes and ASD,^59^ along with previous literature^60^^,^^61^ has indicated possible association between heterozygous *HECTD4* LoF variants with ASD. Together, these multi-omic findings suggest a possible expansion of the pathogenic variant spectrum for *HECTD4*, wherein tandem repeat expansions may lead to a milder form of the neurodevelopmental disorder following an autosomal dominant inheritance pattern.

Apart from variant types, RNA-seq also suggested possible widening of phenotypic spectrum, showing RNA-seq’s extended capability of providing more in depth understanding of reported diseases. For example, *WDR11* was detected as an aberrant expression outlier (FC = 0.55; FDR = 0.0014) in proband B114 with both genital and intellectual developmental phenotypes. *WDR11* has been associated with two diseases with conflicting and limited understanding,^62^, ^63^, ^64^, ^65^ including “Hypogonadotropic hypogonadism 14 with or without anosmia” (OMIM #614858) in an autosomal dominant form with mainly missense variants and “Intellectual developmental disorder” (OMIM #620237) in an autosomal recessive form with LoF variants. Our proband’s phenotype overlapped with both diseases, suggesting a possible expansion of the phenotypic spectrum of *WDR11*-related diseases. This prompted the identification of a heterozygous deletion NC_000010.11:g.(119969782_133655950)del from WGS, supported by 26 under expressed genes from our AE result (FDR < 0.1; FC = 0.32–0.61). This deletion contained *WDR11* and none of the remaining genes within the deletion were found to provide likely explanations of the patient’s phenotypes. For both cases discussed (B93 with *HECTD4,* and B114 with *WDR11*), WGS had no positive findings from gene panels associated with patient phenotypes, therefore we highly suspect the clinical relevance of *HECTD4* and *WDR11* in these two cases. Nevertheless, further studies and deeper understanding are required to confirm our hypothesis and establish a diagnosis.

### Fibroblast RNA-seq

Eleven of the 102 patients in our cohort had fibroblast samples available for RNA-seq analysis; for one of them (BF4), fibroblast RNA-seq confirmed the positive blood RNA-seq finding. Although the other ten cases had negative results in blood RNA-seq analysis, one of these cases was solved by hypothesis-free fibroblast RNA-seq, as reported in our previous publication.^47^ Interestingly, fibroblast RNA-seq detected an RNA signature absent in blood RNA-seq. Proband BF1 presented with global developmental delay, mild intellectual disability, leukoencephalopathy, progressive spasticity, liver derangement, progressive renal failure with protein-losing enteropathy, and suspected bilateral optic atrophy. A significant number (n = 26) of histone genes were detected as aberrantly expressed in fibroblasts, but not in blood, when compared to controls (FC = 2.3–7.9; FDR = 2.9 × 10^−7^-0.054). This guided the identification of compound heterozygous variants (NR_023317: n.[28C>T]; [35G>A]) in a small nuclear RNA gene *RNU7-1*, which is responsible for mRNA processing and associated with Aicardi-Goutières syndrome (OMIM #619487).^66^ The RNA-seq finding combined with an abnormal interferon signature (interferon score of 18.6; decimalised age of 19.3 years) consistent with the disease enabled the application of PP4_strong evidence. This led to a classification of likely pathogenic for both variants (PM3, PM2_supporting, PP4_strong) and the establishment of a diagnosis (Supplementary Information S3 and Supplementary Table S3).

## Discussion

Through the application of blood (and fibroblast) RNA-seq analysis on 102 patients with genetically undiagnosed disorders, our pipeline aided the interpretation of 21.6% (22/102) of cases. With an incremental diagnostic yield of 11.8% (12/102) based on currently known gene–disease associations, nine of these cases demonstrated aberrant splice events. Blood RNA-seq alone led to a diagnosis in 10.8% (n = 11) of cases, which is slightly higher than reported in previous studies (∼ 8.5%) (Supplementary Table S1).^24^^,^^28^^,^^29^^,^^36^^,^^38^^,^^44^ This demonstrates the diagnostic value of blood RNA-seq in rare genetic diseases, even for clinically well-defined disorders like Noonan syndrome, with particular benefits in identifying mutations with splicing phenotypes. In addition to facilitating diagnoses, our findings show that blood RNA-seq can serve both clinical and scientific value through new disease mechanism discoveries, re-evaluating VUS candidates (e.g., downgrading pathogenicity), and facilitating hypothesis-free research to aid more patients suffering from similar diseases.

Regarding the clinical integration of RNA-seq and translatability, our analysis addressed an ongoing debate on the “most appropriate” practice for RNA-seq integration into the diagnostic workflow. Two approaches are possible: (1) hypothesis-driven, where RNA-seq is used to validate candidate findings from DNA-level tests, and (2) hypothesis-free, where the analysis is primarily driven by RNA-seq results with no prior genetic candidates. More specifically, our findings address whether a hypothesis-free approach (i.e., RNA-centric or RNA-first) is beneficial for case interpretation. Upon reviewing 18 studies that used RNA-seq to aid genetic diagnosis (Supplementary Table S1), we observed that the hypothesis-driven approach and hypothesis-free approach aided interpretation of 72.1% (147/204) and 27.9% (57/204) cases, respectively. For cases achieving diagnosis (n = 150), a hypothesis-free approach was responsible for 25.3% of the diagnoses. Despite the seeming advantage of a hypothesis-driven approach in increasing the diagnostic rate owing to the detection of “low-hanging fruit” given prior candidate genes, the hypothesis-free approach in our study has demonstrated its importance in reprioritising variants missed in previous genetic tests. The hypothesis-free approach also served to identify genes with limited understanding, enabling the establishment of new gene–disease associations. Our results support the utility of blood RNA-seq in both hypothesis-driven and hypothesis-free approaches. The proportion of cases aided by each type of analysis was approximately equal (50:50), showing that they are equally important and useful in solving undiagnosed cases. Aligning with the literature, hypothesis-free analysis reprioritised/guided variant identification in DNA data (n=3), identified underlying variants in RNA (n = 2), and aided discovery of disease mechanisms (n = 6). This supports the application of RNA-seq regardless of whether prior candidates have been indicated in undiagnosed cases. The applicability of hypothesis-free approach has also been demonstrated by our workflow, with a resultant handful of candidate genes identified for further manual inspection. The capability of our pipeline to analyse samples from heterogeneous diseases, facilitated by the autoencoder, further enhanced its generalisability and translatability without having to accumulate samples from the same disease cohort for resource-demanding patient-specific studies. Our pipeline maximises benefits in healthcare management of patients with rare diseases by providing more timely diagnoses, which are essential for clinical management decisions regarding precision treatments and supportive care planning.

Despite the promising clinical diagnostic utility of transcriptomics, at present, there is a lack of a consensual framework regarding its clinical application. Zhao et al.^43^ recently proposed a validation framework for benchmarking RNA-seq workflows across different laboratories, while our study demonstrated different considerations required when applying RNA-seq results into the variant curation pipeline. According to the ACMG/AMP guidelines,^20^^,^^45^^,^^51^ results from RNA-seq can be applied to variant curation mainly through two criteria: (1) PVS1 (i.e., a null variant identified in a gene with LoF as an established disease mechanism), and (2) PP4 (i.e., phenotypic specificity).

For PVS1, RNA-seq can provide further information in three aspects to determine the final strength of this criterion.^45^^,^^51^ First, it provides functional data on whether the transcript has undergone nonsense-mediated decay (NMD) through aberrant expression (AE) findings. The most widely used rule to predict NMD is that a transcript will escape NMD if the premature termination codon (PTC) is situated in the last exon or the last 50–55 bp of the second-to-last exon.^51^^,^^67^ However, our data showed that out of nine newly diagnosed cases with aberrant splicing events, only one case had concordant results between NMD prediction and RNA-seq (Supplementary Table S6 and Supplementary Informations S1 and S2). This discordance was also reported in previous studies,^38^^,^^43^ suggesting that this might be partially attributed to variable NMD efficiencies in different tissues. To remain conservative, we suggest that the “predicted to undergo NMD” pathway in the PVS1 decision tree should only be selected if RNA-seq clearly demonstrates NMD (i.e., a statistically significant decrease in fold change that matches the zygosity).^51^ Evidently, RNA-seq is able to provide a more accurate understanding of NMD efficiency and hence avoid the overestimation of “pathogenicity” for the variant under investigation. Second, RNA-seq results act as functional evidence (PVS1 criterion) through aberrant splicing (AS) findings that replaces *in silico* predictions (PP3 criterion), the latter of which has a lower strength of pathogenicity (Tables 2 and 3 and Supplementary Informations S1 and S2). Additionally, aligning with previous studies,^38^^,^^68^ RNA-seq can provide further refined information on splicing events compared to *in silico* predictions, demonstrated by 4/8 newly diagnosed cases with splice variants in our study (Supplementary Table S6 and Supplementary Informations S1 and S2). An accurate understanding of aberrant events is of paramount importance for future development of specific nucleic acid therapies.^69^ Third, RNA-seq provides more quantifiable results for assessing the “completeness”^45^ of the splice event as opposed to more traditional methods like gel electrophoresis. While explicit thresholds have yet to be established, our study considered an aberrant event complete when RNA-seq results match the zygosity of the variant.

**Fig. 2:**
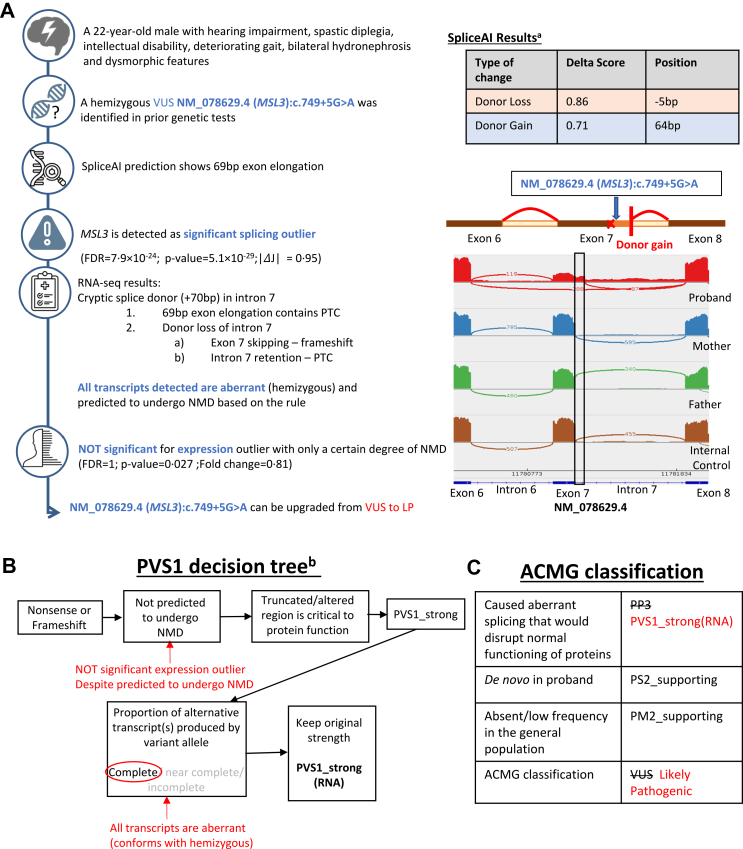
**Upgrading a VUS variant to likely Pathogenic in case B68.** (A) The timeline shows the interpretation and sequence of analysis of this case, from identifying a *MSL3* VUS candidate variant to upgrading the variant to likely pathogenic. A resultant intron retention was initially predicted based on SpliceAI results. Statistical tests performed are in-built executions of the DROP pipeline.^39^, ^40^, ^41^, ^42^^a^The results are directly derived from SpliceAI^70^ webtool (https://spliceailookup.broadinstitute.org/). (B) This shows the pathway taken for PVS1 decision tree, indicating how RNA-seq results were incorporated. ^b^The decision trees are referenced from Abou Tayoun et al.^51^ and Walker et al.^45^ (C) Criteria in black are used before RNA-seq results were available. Criteria in red are changes and final classification made based on RNA-seq results. J: Intron Jaccard Index; FDR: false discovery rate; LP: likely pathogenic; NMD: nonsense-mediated decay; PTC: premature termination codon; VUS: variant of uncertain significance.

**Fig. 3:**
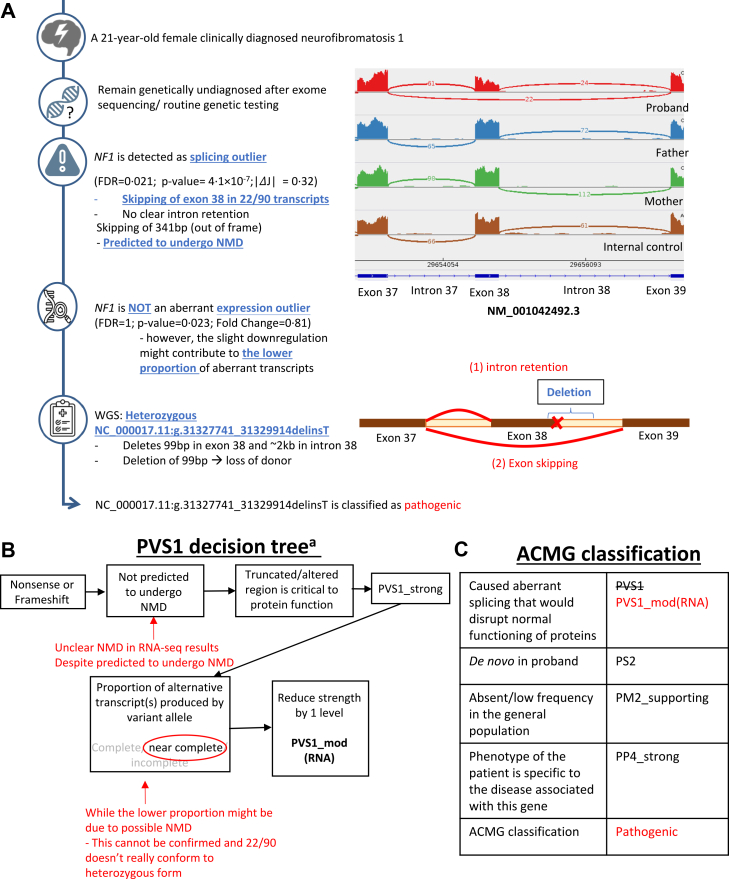
**RNA-seq results guided the identification of a pathogenic variant for case B105.** (A) The timeline shows the interpretation and sequence of analysis of a case with clinically diagnosed neurofibromatosis 1. RNA-seq results guided the identification of pathogenic *NF1* variant in whole genome sequencing. 2 outcomes (i.e., intron retention and exon skipping) are possible in the event of donor loss based on the prediction. Statistical tests performed are in-built executions of the DROP pipeline.^39^, ^40^, ^41^, ^42^ (B) This shows the pathway taken for PVS1 decision tree, indicating how RNA-seq results were incorporated. ^a^ The decision trees are referenced from Abou Tayoun et al.^51^ and Walker et al.^45^ (C) Criteria in black are used before RNA-seq results were available. Criteria in red are changes and final classification made based on RNA-seq results. J: Intron Jaccard Index; FDR: false discovery rate; NMD: nonsense-mediated decay.

As an illustration of the RNA-seq application, two examples are described in cases B68 (Fig. 2) and B105 (Fig. 3):

**In B68**, RNA-seq identified *MSL3* as a significant splicing outlier (FDR = 7.9 × 10^−24^, |*Δ*J| = 0.95). For the curation of the hemizygous NM_078629.4(*MSL3*): c.749+5G>A variant, PP3 is replaced with PVS1_strong (RNA) by choosing “not predicted to undergo NMD” and “complete” for the aberrant events. This is due to the uncertainty of NMD in AE module (FC = 0.81; unadjusted p-value = 0.027; FDR = 1) and events conforming with hemizygosity, supported by a |*Δ*J| value near 1.
**In B105**, RNA-seq flagged *NF1* as a significant splicing outlier (FDR = 0.021; |*Δ*J| = 0.32), clearly showing 22/90 transcripts skipping exon 38. This guided the identification of the heterozygous NC_000017.11:g.31327741_31329914delinsT mutation through srWGS. The variant is classified as pathogenic with PVS1_mod(RNA) by choosing “not predicted to undergo NMD” and “near complete” for the splice event. This is attributed to the insignificant fold change (FC = 0.81; unadjusted p-value = 0.023; FDR = 1) and the aberrant event not conforming to heterozygosity.

For PP4 (phenotypic specificity), this criterion can be applied in situations where downstream events of the mutation are detected instead of the variant-carrying gene. Demonstrated by BF1, RNA-seq analysis identified enrichment of overexpressed histone genes, which can be regarded as “RNA phenotypes”. This enables the application of PP4 in pathogenicity classification and hence, achieving diagnosis (Supplementary Information S3). Similarly, for *DDX39B* cases (Table 3 and Supplementary Information S2), the significantly increased number of aberrant RNA events (i.e., RNA signature) can serve as an “RNA phenotype” for the application of PP4 in variant curation for future diagnosis. However, PP4_supporting is suggested, as multiple other disease genes have been reported to present similar aberrant RNA phenotypes.^71^^,^^72^ In another case (BF4), RNA-seq flagged *GFM1* as significantly downregulated, which later guided the identification of a 104.5 kb homozygous deletion that included 11 expression enhancers of *GFM1*. This RNA finding acts as a “phenotype” specific to loss of *GFM1* enhancers and supported the LP classification of the deletion mutation through PP4_strong (Table 3 and Supplementary Information S2).

Despite the strategies applied by our study, the current curation framework still requires further work to better incorporate multiple aspects of RNA-seq data. Several questions remain. For example, does the “completeness” of an event refer to expression and/or splicing data? How should we interpret isoform switching-related data? Which algorithms and thresholds should be implemented for analysis? The current thresholds, which are based heavily on adjusted p-values, are likely leading to the overlooking of aberrant events, which might also explain the substantial inconsistency between NMD and expression data. As exemplified by case B56, manual curation identified a statistically insignificant aberrant splice event in *RARS2*, resulting in the upgrading of a previously detected VUS to LP. In addition, continuous amendments are required to determine the specificity of an RNA signature and the threshold of aberration. Further benchmarking and determination of the “best” workflow for transcriptomics implementation are required to maximise its benefit in clinical genetics.

We acknowledged several limitations in our study. First, while the inclusion of 2 additional patients with known *DDX39B* variants might have slightly inflated the number of cases our pipeline aided, they are not included in our incremental diagnostic yield. More importantly, their inclusion led to the discovery of a potential RNA signature that might act as a future diagnostic entity. RNA signatures have already reported in multiple different genes including *RNU4ATAC* variants in RNU4atac-opathy,^71^ *RNU4-2* variants in ReNU syndrome^73^ or *RNU12* variants in CDAGS syndrome.^72^ Another case (BF1) in our cohort has also indicated the potential presence of an RNA signature in *RNU7-1* for Aicardi-Goutières syndrome. Not only does it potentially guide the identification of variants, but it also supports the variant’s pathogenicity through the application of PP4. Second, other omics technologies will be required to complement transcriptomics for variants that do not have an RNA phenotype. ClinVar reports that 64% of VUS are missense variants, which likely affect protein stability/folding.^19^ The clinical value of incorporating proteomics into the RNA-seq diagnostic pipeline has been proven in a mitochondrial cohort and our own neurodevelopmental cohort, yielding an incremental diagnostic rate of 22.3% and 14.7% upon DNA-level analysis, respectively.^47^^,^^74^ ^preprint^ Approximately half of these diagnosed cases were only detected in proteomics in both papers. Further studies are required to establish similar systematic workflows incorporating multi-omics technologies (e.g., proteomics, epigenomics) to advance clinical genetics in both diagnosis and precision treatments. Third, other tissues might need to be considered in cases where blood transcriptomics might not be informative, as in our case BF1. Multi-omics data are more subject to tissue-specific variabilities than “static” DNA. Multiple studies have shown that fibroblasts, while more invasive to obtain, have a higher coverage of Mendelian disease genes.^35^^,^^43^ Our previous study has also demonstrated the diagnostic utility of amniocyte transcriptomics, with a comparable coverage of expressed genes to fibroblasts.^46^ There are currently emerging resources to aid the decisions on clinically accessible tissues (CAT) for analysis. For example, a score to estimate the minimum required sequencing depth (MRSD)^75^^,^^76^ for a gene in the CATs, and MAJIQ-CAT^77^ which estimates how representative a CAT is to the tissue of interest in splicing. The choice of CATs should consider all these aspects, including the invasiveness of tissue sampling. If genes are not expressed in all CATs, transactivation of silent genes or transdifferentiation of CATs into the tissue of interest have also been shown to aid case interpretation.^78^, ^79^, ^80^ Nevertheless, further validation and benchmarking are still required for the application of these data into the clinical diagnostic workflow.

In conclusion, our study highlights the clinical diagnostic and scientific value of blood RNA-seq in both hypothesis-driven and hypothesis-free approaches within a heterogeneous rare disease cohort. It enabled diagnosis, discoveries, and the identification of potential diagnostic signatures. While suggesting an initial framework for its clinical implementation, our study also directed aspects where further guidance is required for the comprehensive incorporation of RNA-seq results into variant curation workflows.

## Contributors

C.P., M.M.C.C. and W.T. wrote the manuscript and both C.P. and M.M.C.C. have accessed and verified the data reported. C.P., M.M.C.C., A.K.Y.K., and K.S.Y. performed data analysis. C.P. was responsible for the visualisation of the data. C.P. and M.M.C.C. performed data curation. C.P., A.K.Y.K., and H.Y.C.L., performed experiments. S.S.F., A.W.S.K., G.C.F.C., H.M.L., R.M.S.W., I.F.M.L., C.W.F., J.Y.L.T., A.P.Y.L., S.S.N.W. and B.H.Y.C. were responsible for patient recruitment. G.C.F.C., H.M.L., R.M.S.W., I.F.M.L., C.W.F., J.Y.L.T., A.P.Y.L., S.S.N.W. and B.H.Y.C. characterised clinical data from patients. W.Y. helped with the setting up of server and bioinformatics pipeline. C.C.Y.M. and B.H.Y.C. conceptualised and supervised the entire project. All authors read and approved the final version of the manuscript.

## Data sharing statement

Clinical, genetic and transcriptomics data are provided in Supplementary tables and informations. DNA and RNA datasets generated from this study are not made publicly available owing to ethical restrictions regarding minor participants and patient anonymity. Requests for the datasets can be directed to the corresponding author, and data will be de-identified before being transmitted to the qualified researchers after review. The codes for DROP pipeline can be accessed through https://github.com/gagneurlab/drop. GTEx datasets were accessed through NCBI dbGaP (the Database of Genotypes and Phenotypes).

## Declaration of interests

There are no existing conflicts of interest to declare.

## Appendix A Supplementary data

Supplementary Informations S1–S3 and Tables S1–S6
