# Supplementary material for: Translating transcriptomics analysis into diagnostic workflows: clinical variant identification and interpretation in hypothesis-driven and hypothesis-free approaches
Source: eBioMedicine. 2026 May 28;128:106313. doi: 10.1016/j.ebiom.2026.106313 (PMC13233572; doi:10.1016/j.ebiom.2026.106313)
Supplement: Supplementary Informations S1–S3 and Tables S1–S6 [file mmc1.docx]

**Supplementary material: Translating Transcriptomics Analysis into Diagnostic Workflows: Clinical Variant Identification and Interpretation in Hypothesis-driven and Hypothesis-free Approaches**

Chingyiu Pang^1^, Martin Man-Chun Chui^1^, Wenshu Tang^2^, Anna Ka-Yee Kwong^1^, Hiu Yu Cherie Leung^1^, Sze-Shing Fan ^1^, Alice Wing-Sze Kwok^1^, Godfrey Chi-Fung Chan^1,3^, Ho Ming Luk^4^, Rosanna Ming-Sum Wong^3^, Wanling Yang^1^, Ivan Fai-Man Lo^4^, Cheuk-Wing Fung^3^, Joanna Yuet-Ling Tung^3^, Anthony Pak-Yin Liu^1,3^, Kit-San Yeung^3^, Sheila Suet-Na Wong^3^, Christopher Chun-Yu Mak*^1^, Brian Hon-Yin Chung*^^1,2,3^

^1^Department of Paediatrics and Adolescent Medicine, School of Clinical Medicine, Li Ka Shing Faculty of Medicine, The University of Hong Kong, Hong Kong SAR, China

^2^ Hong Kong Genome Institute, Hong Kong Special Administrative Region, China

^3^Department of Paediatrics and Adolescent Medicine, Hong Kong Children’s Hospital, Hong Kong SAR, China

^4^Department of Clinical Genetics, Hong Kong Children’s Hospital, Hong Kong SAR, China

*Co-last authors

^ Corresponding author

Brian Hon-Yin Chung

Room 115, 1/F, New Clinical Building, Queen Mary Hospital, Hong Kong

Email: bhychung@hku.hk

Phone: (852) 2255-4482

Fax: (852) 2855-1523

**Table of Contents**

| **Supplementary Information 1:** Description of 10 cases aided by Hypothesis-Driven Blood RNA-seq Analysis | p.3-11 |
| --- | --- |
| **Supplementary Information 2:** Description of 11 cases aided by Hypothesis-free Blood RNA-seq Analysis | p.11-20 |
| **Supplementary Information 3:** Description of BF1 case aided by Fibroblast RNA-seq | p.21 |
| **Supplementary table 1:** Review of 18 RNA-seq Studies | p.22-25 |
| **Supplementary table 2:** Details of Control Data from GTEx | p.26 |
| **Supplementary table 3:** Clinical Information and Outcome for 102 Probands | p.27-44 |
| **Supplementary table 4:** Cohort Demographics (sex-disaggregated data) | p.45 |
| **Supplementary table 5:** Number of Outliers Before and After Filtering for OMIM genes for 11 Hypothesis-free cases | p.46 |
| **Supplementary table 6:** Comparisons between predictions and RNA-seq results in expression and splicing for 9 cases with aberrant splicing events | p.47-49 |

**Supplementary Information 1: Description of 10 cases aided by Hypothesis-Driven Blood RNA-seq Analysis**

**B10**

This participant^1^  was presented with antenatal onset dilated cardiomyopathy with moderate mitral regurgitation and sporadic isolated ventricular ectopics. Other clinical features include developmental delay, suspected cortical visual impairment, poor feeding, deranged and progressively deteriorating renal function, history of lactic acidosis, neutropenia. This patient was initially suspected of mitochondrial disorder, where whole exome sequencing (WES) results came back negative, and therefore, subsequently referred for whole genome sequencing (WGS).

By WGS, a maternally inherited hemizygous NM_000116.5 (*TAFAZZIN*): c.284+5G>A, r.[284_285ins[gugaa;284+6_284+106], 284_285ins[gugaa;284+6_284+87]], p.(Thr96*) variant was identified. *TAFAZZIN*, previously known as *TAZ* gene, is associated with Barth Syndrome (OMIM# 302060) with X-linked recessive inheritance. This syndrome is characterized as an X-linked mitochondrial disease with dilated cardiomyopathy, skeletal myopathy, neutropenia and abnormalities in the ultrastructure of mitochondria.^2^ This variant is absent from general populations and spliceAI^3^  has predicted a disruption of splicing, likely causing 106bp exon elongation. The pathogenicity classification of this frameshift variant remains to be variants of uncertain significance (VUS) according to the ACMG/AMP guidelines.^4–6^ (PM2_supporting and PP3). Our RNA-seq data identified *TAFAZZIN* as an aberrant splicing outlier (|𝛥J| = 0·5; FDR=0·0000079; unadjusted p-value= 4.6×10^-11^), showing two aberrant transcripts with 106bp exon elongation (major) and 87bp exon elongation (minor) which matches with hemizygosity. This frameshift variant is predicted to undergo nonsense-mediated decay (NMD), however, this gene was not detected as a significant expression outlier (unadjusted p-value = 0·45; fold change = 0·91; FDR=1). Our RNA-seq data provided more functional evidence to allow the application of PVS1 criteria in place of PP3, ^5,6^ while provided more refined information when compared to spliceAI^3^  predictions. Along with additional laboratory findings including mild increase in urine organic acids and elevated MLCL/CL ratio on dried bloodspot and lymphocytes, this variant can subsequently be upgraded to a likely pathogenic classification (ACMG/AMP^4–6^ : PVS1_strong(RNA), PM2_supporting and PP4_strong).


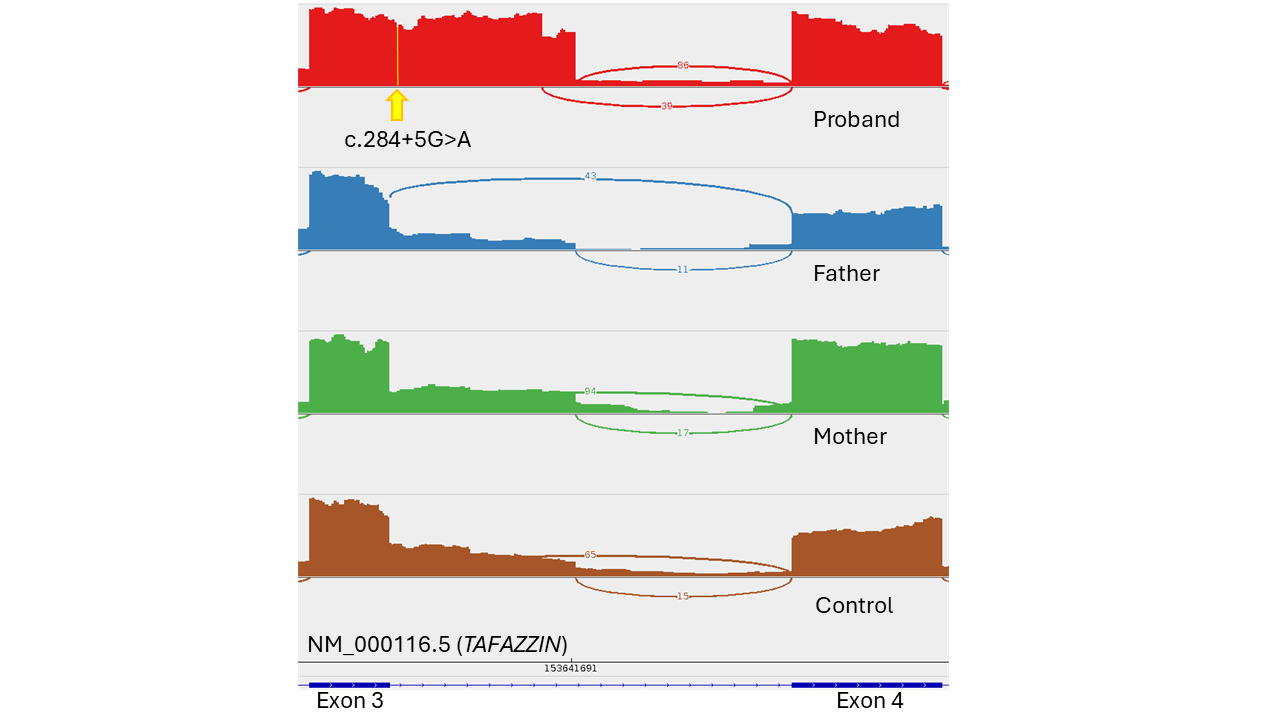


*B10 (RNA-seq IGV): In the father, mother and control, who all do not carry the c.284+5G>A variant, most of the transcripts spliced out the entire intron 3 as expected, with a small proportion of transcripts having 106 exon elongation. However, in the proband (with c.284+5G>A), there is a complete absence of the normal expected splicing (removing intron 3), instead most of the transcripts have 106 exon elongation of exon 3 with minor transcripts having 87bp exon elongation.*

**B11**

This patient ^1^ was presented with hypertrophic cardiomyopathy, left coronary artery fistula to the right ventricle, secundum atrial septal defect. Additional clinical phenotypes include developmental delay, recurrent hypoglycaemia and facial features of Noonan syndrome. Trio WES results were negative and was therefore, referred for WGS.

WGS identified two compound heterozygous variants in *LZTR1* inherited from each parent -- paternally inherited NM_006767.4: c.1943-256C>T, r.1943_1944ins1943-378_1943-262, p.(Gly648_Thr649ins*36) and maternally inherited NM_006767.4: c.1261-3C>G, r.[1260_1261ins[1261-22_1261-4;gag],r.1260_1261ins[1260+1_1261-4;gag]], p.[(Phe421Serfs*13), (Phe421Valfs*21)] variant. *LZTR1* is associated with Noonan syndrome 2 (OMIM# 605275) with autosomal recessive inheritance. Noonan syndrome is characterized by distinct facial features, short stature and cardiac anomalies.^7^  The paternally inherited variant NM_006767.4: c.1943-256C>T was classified as likely pathogenic owing to its rarity in the general population (gnomAD v4.1.0 = 0.000079)^8^ , being *in trans* with multiple pathogenic or likely pathogenic variants in affected patients, and near complete NMD shown in reverse transcription polymerase chain reaction (RT-PCR)^9^ (ACMG/AMP^4–6^ : PVS1(RNA), PM2_supporting, PM3).  The maternally inherited variant NM_006767.4: c.1261-3C>G was found rare in the general population (gnomAD v4.1.0 = 0.0000012)^8^ , *in trans* with multiple pathogenic/likely pathogenic variants in affected patients and predicted to affect splicing causing a 22bp exon elongation by spliceAI^3^, therefore remains to be a VUS (ACMG/AMP^4–6^ : PM2_supporting, PM3 and PP3). Our RNA-seq analysis identified *LZTR1* as a significant aberrant splicing outlier, demonstrating two events correlating with each of the variants while matching the zygosity. For the paternally inherited variant, in-frame 117bp cryptic exon was detected (|𝛥J| = 0·38; FDR=0·032; unadjusted p-value=1.8×10^-7^), confirming the predictions from spliceAI^3^  and results from RT-PCR.^9^ For the maternally inherited variant, two aberrant frameshift transcripts were detected with 22bp exon elongation and intron 11 retention respectively (|𝛥J| = 0·63; FDR = 0·00095; unadjusted p-value= 3.2×10^-8^). This gene was predicted to undergo NMD, however, only a certain degree of NMD has been detected (FDR =1; unadjusted p-value=0·0018; Fold change=0·75). RNA-seq results confirmed previous findings for the likely pathogenic c.1943-256C>T variant while provided additional functional evidence to upgrade the VUS c.1261-3C>G variant to likely pathogenic by replacing PP3 with PVS1_strong (ACMG/AMP^4–6^ : PM2_supporting, PM3 and PVS1_strong(RNA)).


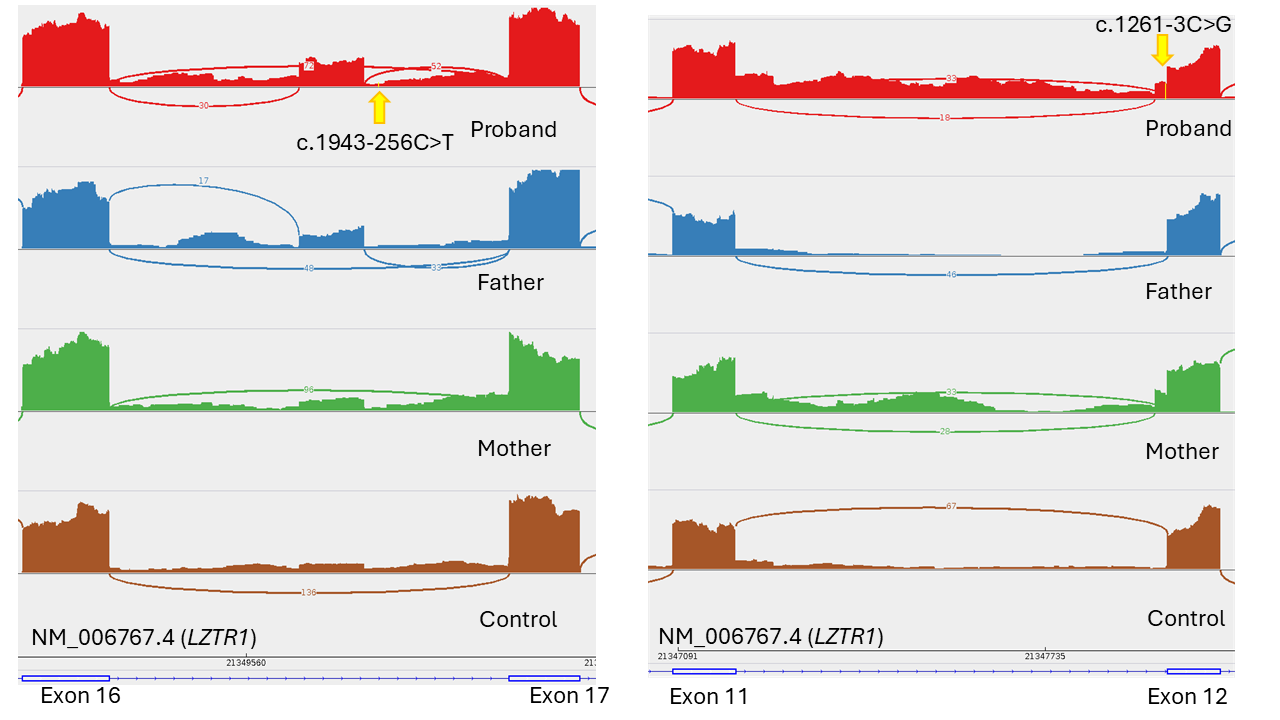


*B11 (RNA-seq IGV): Region affected by the paternal variant (left) – both father and proband, who carries the 1943-256C>T variant, demonstrated a clear 117 bp cryptic exon in intron 16. This is not observed in mother and control, who do not carry the variant. Region affected by the maternal variant (right) – both the mother and proband, carrying c.1261-3C>G, show a 22bp exon elongation of exon 12 and slight intron 11 retention. This is not observed in father and control, who do not carry the variant.*

**B29**

This patient was presented with macrocephaly, history of global developmental delay and dysmorphic features including frontal bossing, down-slanting eye and low set ears. Clinicians suspected Sotos syndrome and obstructive sleep apnoea syndrome due to snoring with hypertrophy of tonsil and adenoids. The patient was subsequently recruited for trio WES analysis.

Trio WES revealed a *de novo* heterozygous *PTEN* variant NM_000314.8: c.209+3A>T, r.165_209del, p.(Arg55_Leu70delinsSer). *PTEN* is associated with PTEN hamartoma tumour syndrome with autosomal dominant inheritance including Cowden syndrome 1 (OMIM# 158350). Children with Cowden syndrome typically presents macrocephaly, frontal bossing and developmental delay.^10,11^ This variant was originally classified as VUS owing to being absent in general population, occurs *de novo* in the proband with paternity and maternity confirmed and predicted to disrupt splicing by spliceAI^3^  leading to a 45bp deletion (ACMG/AMP^4,6^ : PM2_supporting, PS2_moderate and PP3). Our RNA-seq analysis identified *PTEN* as a significant aberrant splicing outlier (|𝛥J| =0·55; FDR = 2·7×10^-11^; unadjusted p-value=2.8×10^-17^), confirming the spliceAI^3^  prediction by demonstrating heterozygous exon 3 (45bp) skipping in the patient. As an in-frame deletion, the aberrantly spliced transcript is not predicted to undergo NMD, which is further supported by our AE data (fold change = 0·97; unadjusted p-value = 0·83; FDR=1). According to the criteria specification registry guidelines modified particularly for *PTEN* from ClinGen, exon 3 is critical to protein function, which was also supported by a previous functional study showing a significantly reduced phosphatase activity of the mutated protein.^12^ With additional function evidence provided by RNA-seq while following the *PTEN*-specific guideline from ClinGen, this variant is further upgraded to likely pathogenic by applying PS3_strong instead of PVS1_moderate in place of PP3 (PM2_supporting, PS2_moderate and PS3_strong).


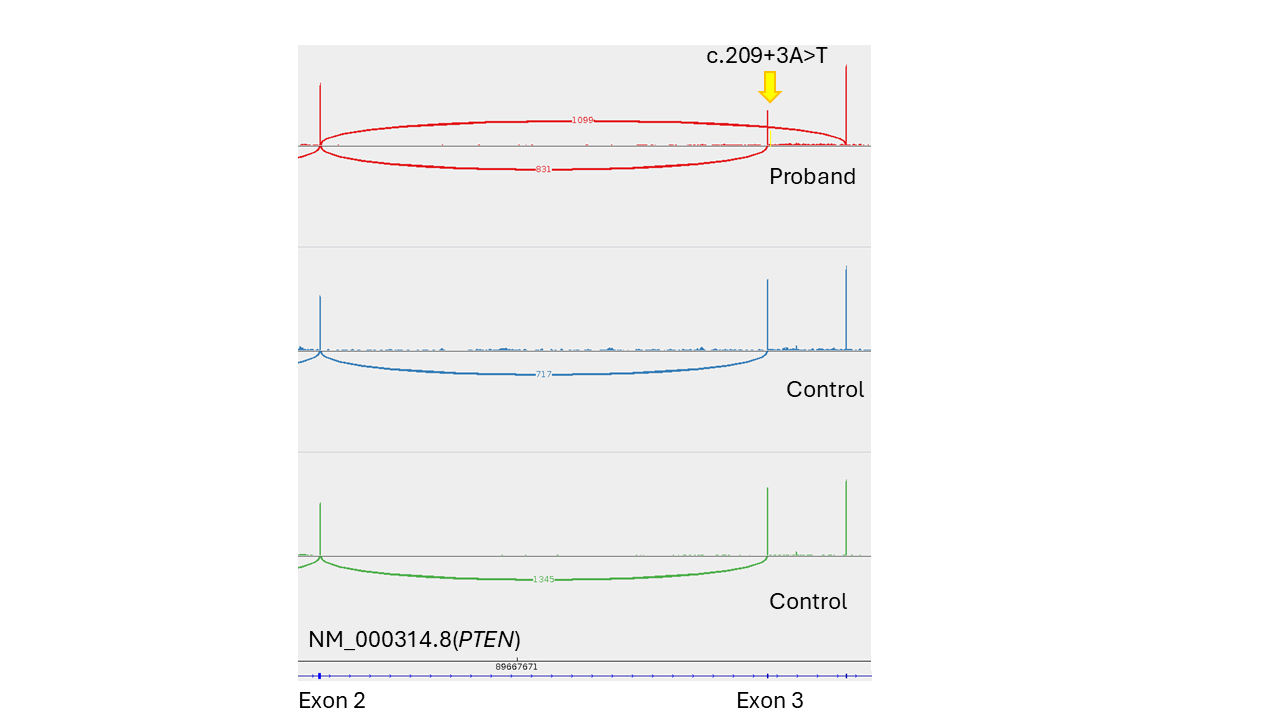


*B56 (RNA-seq IGV): Carrying c.209+3A>T, Proband’s IGV clearly showed more than half of the transcripts were found to skip exon 3 of PTEN. This event was clearly not found in other two controls who do not carry the same variant.*

**B56**

The participant had suspected movement disorder with multiple motor phenotypes including global developmental delay (motor delay), severe hypotonia, progressive cerebellar ataxia, intentional tremor, unsteadiness and dystonia. Simultaneously, this patient was affected by cognitive decline, memory loss/regression, slow speech and epilepsy. This patient was subsequently recruited for trio WES analysis.

Trio WES results identified compound heterozygous variants in *RARS2* gene inherited from each parent – parentally inherited NM_020320.5: c.685C>T, r.685c>u, p.(Arg229*) variant and maternally inherited NM_020320.5: c.1238-28T>G, r.[1237_1238ins[1238-90_1238-1;1238-28u>g], 1237_1238ins[1238-74_1238-1;1238-28u>g], 1237_1238ins[1237+1_1238-1;1238-28u>g]], p.[(Thr413_Thr414ins*13),(Thr413Asnfs*15),(Thr413Serfs*11)] variant. These variants were further confirmed with trio WGS analysis. *RARS2* is associated with Pontocerebellar hypoplasia type 6 (OMIM# 611523) with autosomal recessive inheritance, which is characterized by seizures, ataxia, and neurodevelopmental regression.^13^ The paternally inherited nonsense variant (c.685C>T p.(Arg229*)) is at extremely low frequency on gnomAD (gnomAD v4.1.0= 0.0000025)^8^ , predicted to undergo NMD owing to the premature termination codon and has been reported *in trans* with another pathogenic variant in literature^14^ . Therefore, it was classified as pathogenic (ACMG/AMP^4–6^ : PVS1, PM2_supporting and PM3). The maternally inherited variant (c.1238-28T>G), classified as VUS, is of extremely low frequency on gnomAD (gnomAD v4.1.0= 0.0000031)^8^ , inherited *in trans* with another pathogenic variant and predicted to disrupt splicing by spliceAI^3^  leading to a potential 90bp exon elongation (ACMG/AMP^4–6^ : PM2_supporting, PM3 and PP3). RNA-seq results did not identify *RARS2* as a splicing outlier (FDR= 0·31; unadjusted p-value = 0·000016; |𝛥J| =0·46), but manual inspection showed that 2 cryptic splice acceptors leading to two types of aberrant transcripts with 74bp and 90bp exon elongation respectively, where some degree of intron 14 retention has also been detected. These match with heterozygosity as expected based on the DNA-level finding. While 74bp exon elongation and intron retention transcripts are out-of-frame, with 90bp exon elongation transcript being in-frame, premature termination codon is being introduced in intron 14, therefore these transcripts are predicted to undergo NMD. According to the AE results, only a certain degree of NMD can be detected (Fold change= 0·77; unadjusted p-value= 0·0045; FDR =1) with similar efficiency of NMD between the paternal and maternal variants. Without a definitive significant expression result, the paternally inherited variant (c.685C>T p.(Arg229*)) is downgraded to likely pathogenic by applying PVS1_strong instead of PVS1 (ACMG/AMP^4–6^ :PVS1_strong(RNA), PM2_supporting and PM3). For maternal variant (c.1238-28T>G), additional functional evidence provided by RNA-seq upgraded it from VUS to likely pathogenic by replacing PP3 with PVS1_strong (ACMG/AMP^4–6^ :PVS1_strong(RNA), PM2_supporting and PM3). Simultaneously, RNA-seq also provided more refined information on aberrant transcripts caused by the splice variant when compared to *in silico* prediction by spliceAI.^3^


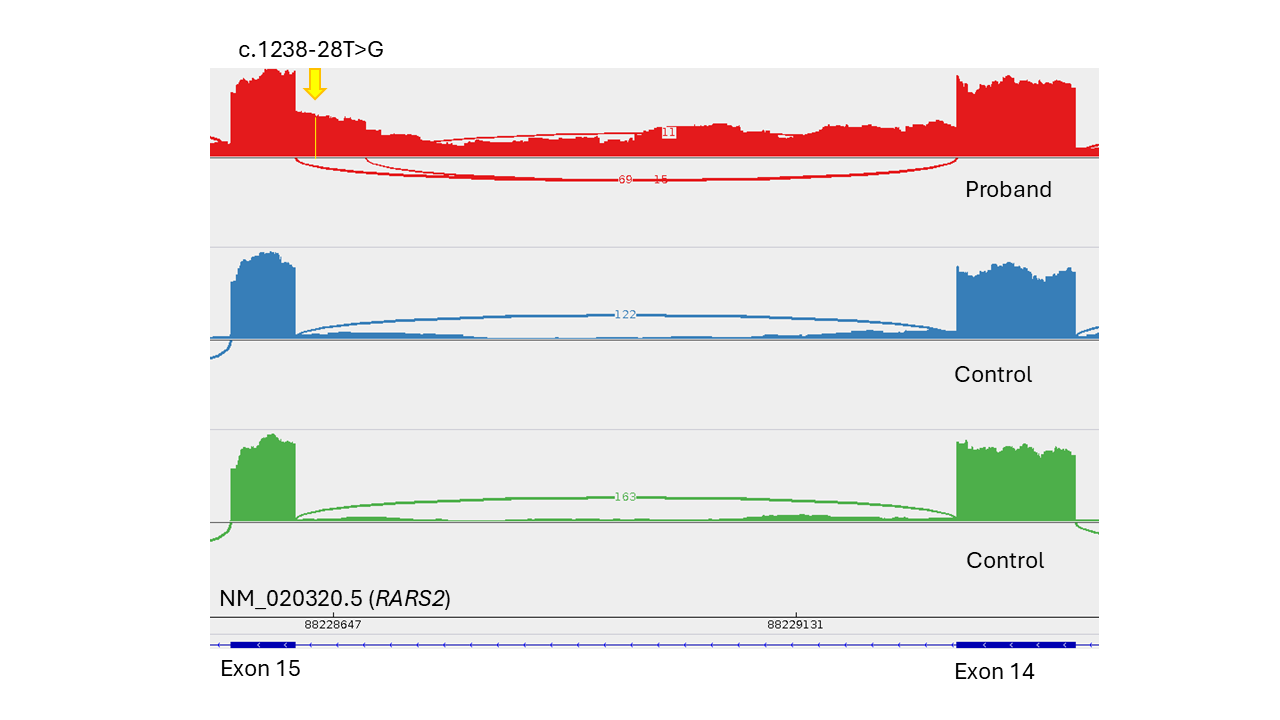


*B56 (RNA-seq IGV): This captures the region affected by the maternal variant c.1238-28T>G. Compared with the two control samples without the variant, proband has 3 aberrant events in intron 14: 1) 74bp exon elongation of exon 15; 2) 90bp exon elongation of exon 15; 3) intron retention of intron 14.*

**B68**

The patient was clinically presented with bilateral moderate-severe hearing loss, bilateral hydronephrosis, spastic diplegia, deteriorating gait, history of global developmental delay, and mild intellectual disability with behavioural problem. The participant also had astigmatism, facial dermal dysplasia and multiple dysmorphic features including low anterior hairline, narrow forehead, down-slanting palpebral fissure, small mouth and prominent ears. This patient was recruited for singleton WES and trio WGS analysis.

Both sequencing analyses revealed a hemizygous NM_078629.4(*MSL3*): c.749+5G>A, r.[589_749del, 749_750ins[gugaa;749+6_749+69], 749_750ins[gugaa;749+6_750-1]], p.[ (Lys197Cysfs*2), (Asn250delinsLys*)] variant, trio WGS confirmed that this variant is not found in parents and is *de novo* in the patient. *MSL3* is associated with Basilicata-Akhtar syndrome (OMIM# 301032) with X-linked dominant inheritance, which is characterized by global developmental delay and intellectual disability, progressive gait disturbance and recurrent facial dysmorphism.^15,16^  Other phenotypes include autism spectrum disorder, visual problems, hearing impairment and symptoms in the urinary system.^15,16^ This variant is predicted to disrupt splicing leading to a 69bp exon elongation by spliceAI^3^ , arisen *de novo* in proband and is absent from general population, hence, concluding to be a VUS variant (ACMG/AMP^4–6^ : PP3, PS2_supporting and PM2_supporting). RNA-seq results detected *MSL3* as a significant splicing outlier (|𝛥J| = 0·95; FDR=7·9×10^-24^; unadjusted p-value=5.1×10^-29^) while revealing multiple alternative forms of aberrant transcripts conforming to hemizygosity including 69bp exon elongation as predicted *in silico*, exon 7 skipping and intron 7 retention. All transcripts are predicted to undergo NMD with exon 7 skipping resulting in a frameshift, and other two transcripts retaining a premature termination codon (intron retention also results in a frameshift). However, according to our expression results, *MSL3* was only detected to undergo a certain degree of NMD (Fold change= 0·81; unadjusted p-value= 0·027; FDR =1). With RNA-seq providing more refined functional evidence revealing multiple aberrant transcripts, it provided sufficient support to upgrade this variant from VUS to likely pathogenic by replacing PP3 with PVS1_strong (ACMG/AMP^4–6^ : PVS1_strong(RNA), PS2_supporting and PM2_supporting).


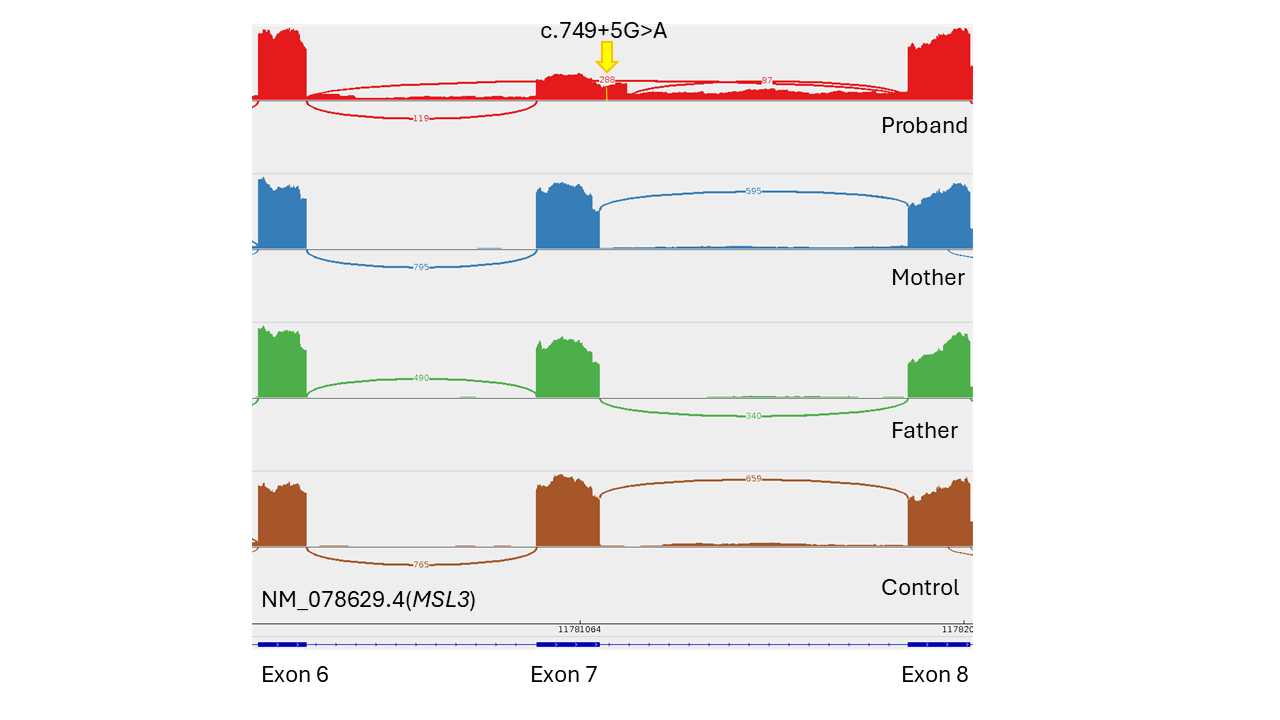


*B68 (RNA-seq IGV): Three aberrant events were detected in proband when compared to mother, father and control, who all do not carry the c.749+5G>A variant: 1) 69bp exon elongation of exon 7; 2) skipping of exon 7; 3) intron retention of intron 7.*

**B119**

This participant had microcephaly with failure to thrive, global developmental delay (discrepant language delay), moderate intellectual disability, autism spectrum disorder and bilateral hearing loss (mild in left ear and profound in right ear). The patient also clinically presented bilateral eye astigmatism, abnormal genitalia, decreased thyroid stimulating hormone and soft dysmorphism. Singleton WES and trio WGS were performed for this patient.

Both sequencing analyses identified a heterozygous NM_133433.4(*NIPBL*): c.64+5G>C, r.-79_333del, p.(Met1_Ser111del) variant in the proband, with a confirmation being *de novo* in trio WGS. *NIPBL* is associated with Cornelia de Lange syndrome 1 (OMIM# 122470) with autosomal dominant inheritance. This syndrome is characterized by distinctive craniofacial appearance, failure to thrive, intellectual disability and autism, with other phenotypes including hearing problems and abnormal genitalia.^17^  This variant was classified as VUS as it is absent in general population, found *de novo* in proband with confirmed paternity and maternity confirmed, and spliceAI^3^  predicted it to cause exon 2 skipping (ACMG/AMP^4–6^ : PM2_supporting, PS2_supporting and PP3). Through our RNA-seq analysis, *NIPBL* was detected as a significant splicing outlier (|𝛥J| =0·22; FDR=0·0055; unadjusted p-value=2.6×10^-7^) showing exon 2 skipping as predicted *in silico*, suggestive of heterozygosity. Deletion of exon 2 removed the canonical initiation codon, however, a potential in-frame alternative start codon is situated in exon 4 leading to the possibility of expression of truncated protein. This is supported by our expression results, where *NIPBL* is not identified as significant expression outlier with no indication of reduced expression (fold change= 1·1; unadjusted p-value =0·059; FDR=1). This functional evidence enabled the replacement of PP3 with PVS1_moderate. Along with additional clinical confirmation on patient presenting soft dysmorphism that matches features of Cornelia de Lange syndrome and syndrome-specific DNA methylation signature, this variant is upgraded to likely pathogenic (ACMG/AMP^4–6^ :PVS1_moderate(RNA), PS2_strong, PM2_supporting and PP4_moderate).


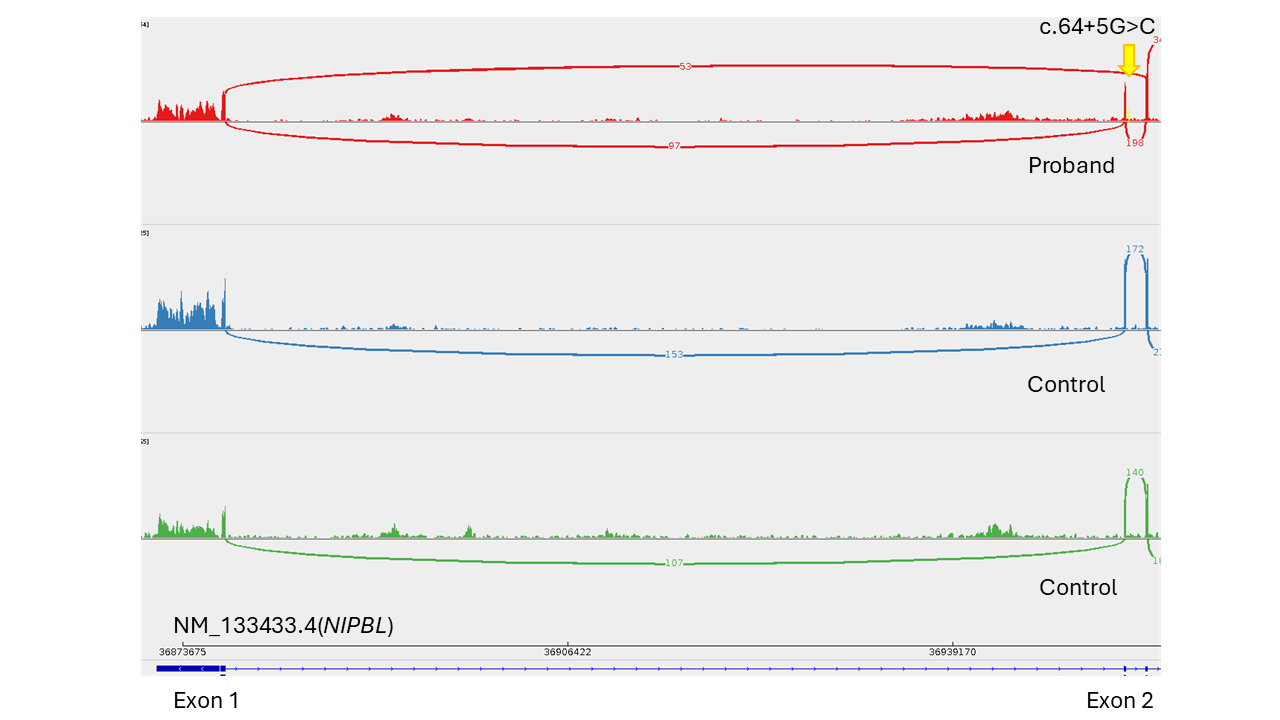


*B119 (RNA-seq IGV): Compared to the two controls without the c.64+5G>C variant, proband clearly shows skipping of exon 2 of NIPBL.*

**B12**

This patient was presented with type 1 jejunal atresia, bilateral inguinal hernia, bilateral undescended testes and penile base haemangioma. With magnetic resonance imaging (MRI) results showing subependymal heterotopia and polymicrogyria, the patient had history of suspected seizure, gross motor delay with verbal expression delay. Other clinical features include ocular issues (bilateral eye corneal opacity, glaucoma and retinal coloboma), micrognathia, macroglossia, syndactyly and sacral dimple. This family was first referred for duo WES, which came back negative, and was subsequently recruited for trio WGS analysis.

Trio WGS identified a heterozygous variant NM_138927.4(*SON*): c.78-2A>C, r.78_83del, p.(Ser26_Gly27del) in the proband inherited from the carrier father, also found in carrier twin brother. *SON* is associated with ZTTK syndrome (OMIM# 617140 ) with autosomal dominant inheritance, a multi-system syndrome which is characterized by developmental delay with intellectual disability.^18^  Other phenotypes like ocular abnormalities, genitourinary manifestations, polymicrogyria, subependymal heterotopia are also found in affected patients.^18^  According to the ACMG/AMP^4–6^ guidelines, none of the criteria can be applied and hence, this variant remains to be classified as a VUS. In particular, while the variant effects the canonical splice site, *in silico* prediction shows 3 possible cryptic splice sites in +/- window that would result in different aberrant transcripts. Cryptic splice site (A) (Figure 1) would lead to in-frame splicing while the other two (B and C) likely lead to frameshift. In our RNA-seq data, *SON* was detected as an aberrant splicing outlier (|𝛥J| = 0·45; FDR = 0·00065; unadjusted p-value=3.1×10^-9^), indicating that cryptic splice site (A) was used in almost half of the transcripts detected, causing a 6bp deletion in exon 2. Supported by expression analysis showing no significant reduced expression of *SON* (fold change=1·1; unadjusted p-value=0·28; FDR=1) with a heterozygous splice event, no indications of the usage of cryptic splice site B and C were demonstrated, and their absence are unlikely owing to the presence of NMD. Along with the unlikely tissue-specific splice events^19^  and lack of segregation in the family, this functional evidence supports a limited pathogenicity of this variant with the in-frame deletion situating outside known functional domain of the gene, hence, lowering the probability of the variant being pathogenic.


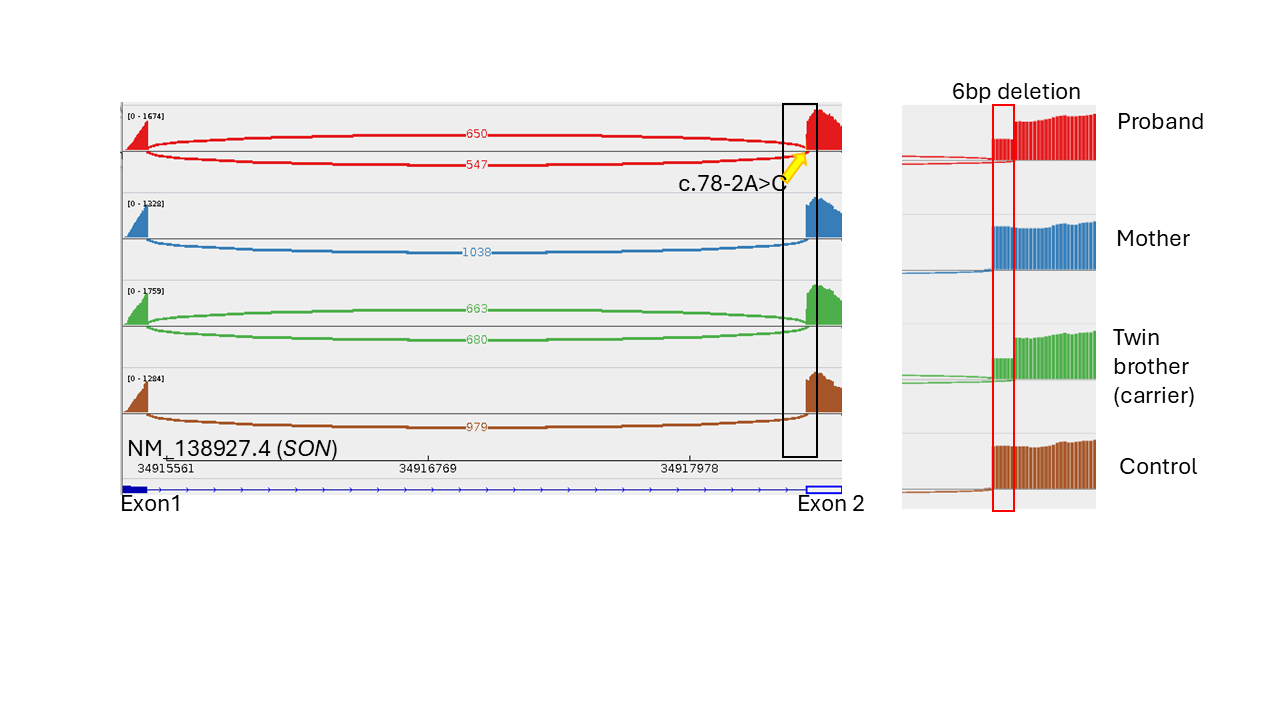


*B12 (RNA-seq IGV): Proband and the twin brother (unaffected), who both carries the c.78-2A>C, demonstrate a 6bp deletion in around half proportion of the transcripts. This event is absent in the mother and control, who do not have the same variant.*

**B37**

This patient was presented with global developmental delay with mild intellectual disability, and MRI results showed possible indications of Dandy-Walker variant or vermian-cerebellar hypoplasia. Mild prominence of ventricular system was also found, suspected to be attributed to under-development or hydrocephalus. Chromosomal microarray identified a *de novo* pathogenic 241.92kb duplication at Xq28 region, however, clinical correlation of duplicated genes and patient phenotypes still required further investigation. This patient was then referred to RNA-seq study.

Our RNA-seq results, confirming the duplication results from chromosomal microarray test, showed significant overexpression of 14 genes in the Xq28 region (FDR<0·1; fold change >2). Among these genes, four genes (*GDI1, RPL10, FAM50A* and *LAGE3*) are further flagged as candidate genes as they are associated with X-linked intellectual developmental disorders (OMIM#: 300849, 300998, 300261 and 301006). While none of the 14 overexpressed genes are reported to have triplosensitivity based on ClinGen curation, previously reported patients with recurrent but variable 0.3Mb copy-number gain at Xq28 also presented similar phenotypes like intellectual disability, ventricular aberrations and dandy-walker malformation.^20^ The duplication regions in the reported cases and our patient contains overlapping genes, and always includes 18 annotated genes, 4 of which are *GDI1, RPL10, FAM50A* and *LAGE3.* Among the contained genes, *RPL10*, *ATP6AP1* and *GDI1* are highly expressed in brain. The severity of the phenotypes likely correlates with the increasing number of copy number, and among which, *GDI1* perfectly correlates with disease severity. In line with the literature, we highly suspect the pathogenicity of these overexpressed genes at Xq28, and specifically *GDI1*. However further investigations are required to establish a definitive gene-disease association, including further clinical phenotyping of cases, *in vitro* experiments of overexpression assay in human cell lines, expression analysis on other available clinically accessible tissues and animal studies that would be supportive of the triplosensitive effects of these genes. Other more recent developments on transactivation/transdifferentiation into clinically affected tissues^21–23^, induced pluripotent stem cells and organoids can also be considered to better understand the effect of the overexpression on affected tissues.

**B48**

This participant^1^  with dilated cardiomyopathy and history of attention deficit hyperactivity disorder with autism was recruited for WES and trio WGS analysis. WES results came back negative while a heterozygous paternally inherited *MYBPC3* variant NM_000256.3:c.1224-80G>A, r.1223_1224ins1224-78_1224-1, p.(Ser408_Lys409insXaa[26]) was detected through WGS. *MYBPC3* has been reported to potentially associate with dilated cardiomyopathy (Left ventricular noncompaction; OMIM# 615396) in autosomal dominant inheritance. This variant is classified as VUS with previously reported minigene splice report assay showing in-frame 78bp insertion. (ACMG/AMP^4–6^: PM4). Our RNA-seq analysis did not detect *MYBPC3* as a significant aberrant splicing outlier, but manual inspection confirmed and provided a more refined picture showing a creation of cryptic acceptor led to a 78bp exon 14 elongation, which also supported the spliceAI^3^ prediction. Expression analysis showed no significant changes compared to the rest of the cohort (fold change=0·9; unadjusted p-value=0·79; FDR =1) as expected for an in-frame exon elongation. This evidence did not enable changes in the criteria applied and hence, this variant remained to be a VUS.


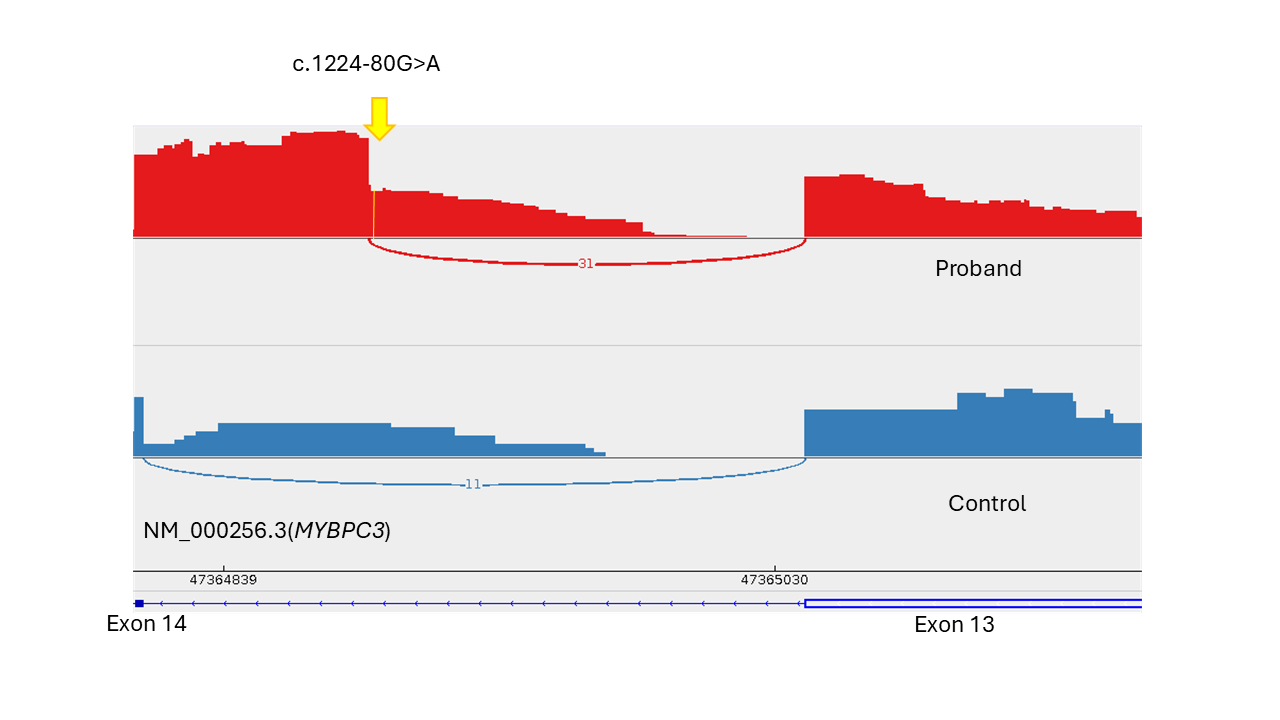


*B48 (RNA-seq IGV): Proband with c.1224-80G>A shows a clear 78bp exon 14 elongation, that is absent in the control without the same variant.*

**B80**

This patient had severe global developmental delay with intellectual disability, failure to thrive, hypotonia, autism spectrum disorder. MRI results showed Dandy-walker malformation. Other clinical features include facial dysmorphism, pectus excavatum, patent foramen ovale, eczema with erythema, and hearing impairment. WES results of this individual were negative and therefore, was subsequently recruited for duo WGS.

WGS analysis identified a heterozygous NM_001197104.2(*KMT2A*):c.4480-18_4480del, r.4480_4482del, p.(Gln1494del) variant that is not inherited from the mother. *KMT2A* is associated with Wiedemann-Steiner syndrome (OMIM# 605130) in autosomal dominant inheritance, which is characterized by developmental delay with intellectual disability and distinct facial features.^24^  Growth restriction, hypotonia and cardiac defects have also been presented in affected individuals.^24^  This variant, being absent from general population and predicted to cause an in-frame deletion of 3bp, was classified as VUS (ACMG/AMP^4–6^: PM2_supporting and PVS1_moderate). Our RNA-seq data identified *KMT2A* as a significant aberrant splicing outlier (|𝛥J| =0·47; FDR=0·00097; unadjusted p-value=1.6×10^-8^) and confirmed the heterozygous deletion of 3bp in exon 12 with no frameshift. Expression analysis did not indicate the presence of significant NMD (fold change=1·1; unadjusted p-value=0·40; FDR=1). This evidence supported the limited damaging effect of the variant and therefore, the classification remains to be VUS.


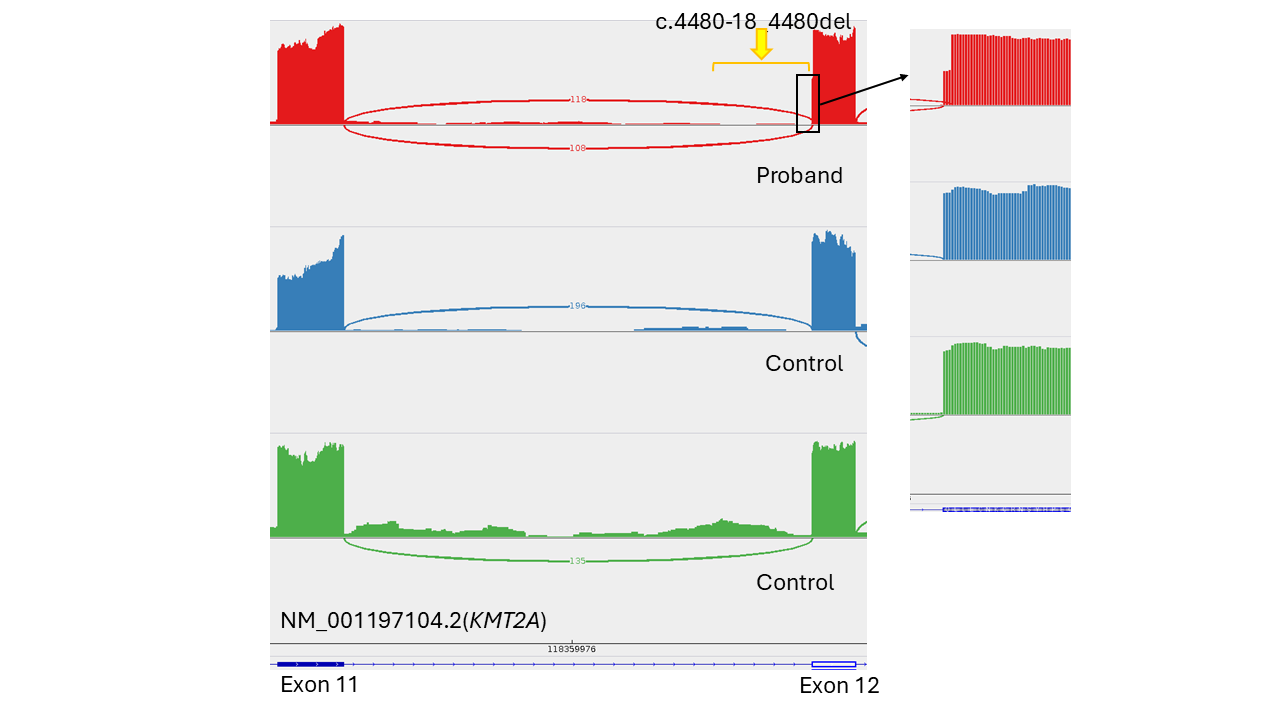


*B80 (RNA-seq IGV): Comparing with the two controls, proband with c.4480-18_4480del has an aberrant 3bp deletion in exon 12 in half of the transcripts.*

**Supplementary Information 2: Description of 11 cases aided by Hypothesis-free Blood RNA-seq Analysis**

**BF4**

This patient was clinically diagnosed of Complex I and IV deficiency mitochondrial disease, presented with cerebral atrophy, epileptic spasm, dystonia, neurogenic bladder, mild ptosis, severe intellectual disability and oromotor dysfunction with feeding difficulty. MRI results showed bilateral basal ganglia hyperintensity. The patient also had recurrent urinary tract infections and skeletal problems. Previous WES test and mitochondrial DNA analysis were unrevealing and hence, this patient was recruited for RNA-seq analysis.

Our RNA-seq results revealed significant under expression of two genes in proximity – *GFM1* (fold change= 0·57; FDR = 0·016; unadjusted p-value=3·1×10^-7^) and *MFSD1* (fold change = 0·03; FDR = 1·1×10^-10^; unadjusted p-value = 7·1×10^-16^). *GFM1* is associated with combined oxidative phosphorylation deficiency 1 (OMIM# 609060) in autosomal recessive inheritance, which can affect CI and CIII-V enzyme activities and causing phenotypes like severe encephalopathy, feeding difficulties and dystonia.^25^ This finding prompted the reanalysis of WES results with quantitative PCR followed by trio WGS analysis, identifying a homozygous 104.5 kb deletion (GRCh37 chr3:158435847-158540316) inherited from each of the parents. This deletion was found to overlap with *MSFD1,* and include 11 enhancers associated with *GFM1* as predicted by GeneHancer.^26^ Among which, 9 were denoted to be “elite” enhancers for *GFM1* (i.e. regions that have a high-likelihood of being an enhancer and a strong gene-disease association). Supported by ENCODE, this deletion contains 77 active enhancer-like regions that are associated with *GFM1.*^27^  To further validate the findings, fibroblast sample was collected from this patient to perform RNA-seq and proteomics analysis, in which *GFM1* was detected as a significant RNA-and-protein expression outlier and *MSFD1* was detected as RNA expression outlier, further validating a >50% reduction of expression of both genes.^28^ With this RNA and proteomics result acting as a specific “phenotypes” for this deletion, it was subsequently curated as likely pathogenic owing to that 1) neither the 104kb deletion nor deletion of similar size have been reported in control population; 2) it occurred as homozygous in the proband and 3) RNA-seq and proteomics results providing functional evidence on the basis that the involvement of other mitochondrial disease associated loci have been ruled out (ACMG/AMP^4–6^:PM2_supporting, PM3_supporting and PP4_Strong).


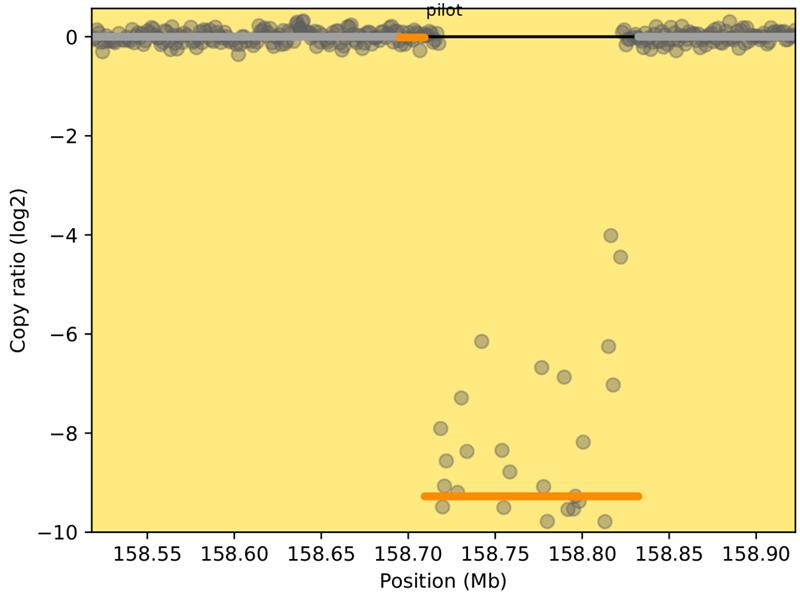


*BF4: This figure shows a decreased copy ratio GRCh38 chr3:158718058-158822527. With a copy ratio (log2) <-1, this conforms with a homozygous deletion of this chromosomal region.*

**B47**

The core features of this patient include global developmental delay with moderate intellectual disability, autism spectrum disorder, macrocephaly, lax joint and central hypotonia. Previous WES results were negative and therefore was recruited for RNA-seq analysis.

Our transcriptomics results identified *KMT5B* as a significant aberrant splicing outlier (|𝛥J| = 0·44; FDR=0·057; unadjusted p-value=2·3×10^-6^). Further manual inspection revealed a heterozygous variant NM_017635.5:c.977+2T>A, r.977_978ins[ga; 977+3_977+5], p.(Arg327*) inherited from unaffected mother, which was further confirmed to be present in the proband through sanger sequencing. *KMT5B* was associated with Intellectual developmental disorder 51 (MRD51; OMIM# 617788) in autosomal dominant inheritance. This disorder was characterized by intellectual disability, autism spectrum disorder, language problems and macrocephaly,^29^ with healthy carriers being previously reported.^30^ This variant was shown to cause a heterozygous 5bp exon elongation in exon 9 causing frameshift, and an inclusion of a premature termination codon. While this aberrant transcript is predicted to undergo NMD, our RNA-seq results counter supports it by showing no significant decrease in *KMT5B* expression (fold change= 0·99, unadjusted p-value=0·89, FDR=1) indicative of escaping from NMD. This counter support of NMD lowered the applicable PVS1 criteria from very strong to strong, avoiding over-estimation of pathogenicity of the variant. Based on the ACMG/AMP^4–6^ guideline, this variant was classified as likely pathogenic owing to 1) variant being absent from the general population ; 2) patient’s phenotypes likely matching with this disorder with DNA methylation signature correlating with MRD51 affected individuals (clinical confirmation prompted by RNA-seq findings) ; 3) RNA-seq results showing disruption of splicing while providing evidence for transcript escaping from NMD (PM2_supporting, PP4_supporting and PVS1_strong(RNA)).


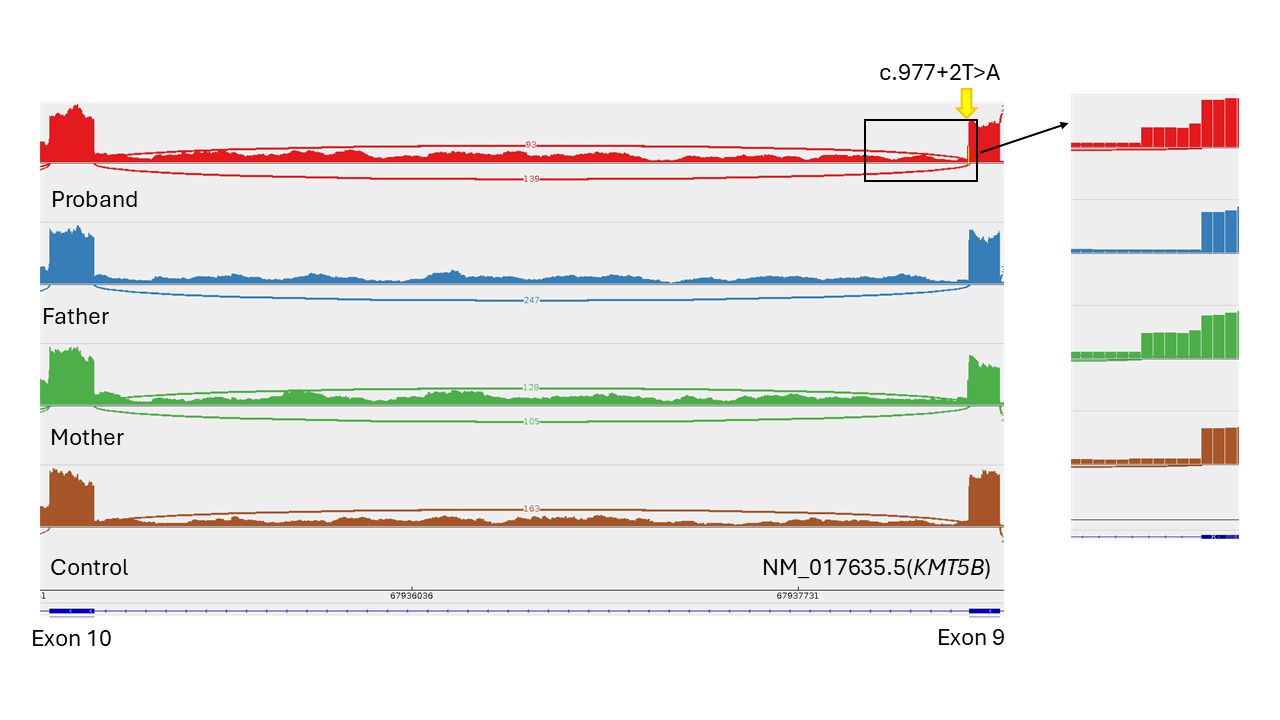


*B47 (RNA-seq IGV): Compared with father, mother and control, who all does not carry c.977+2T>A, proband’s data clearly showed a 5bp exon elongation of exon 9.*

**B84**

This patient was presented with muscle weakness with suspected distal myopathy, bilateral lower limbs muscle wasting, abnormal walking gait with frequent falls, microcephaly, learning disability, speech delay and attention deficit hyperactivity disorder. Other features include astigmatism, scoliosis, swan-like deformities of the fingers, feet deformity and bicuspid aortic value with moderate to severe aortic insufficiency.

Being recruited for our RNA-seq study, 5 genes (*CTCF*, *CARMIL2*, *ATP6V0D1*, *ACD* and *RIPOR1*) from chromosome 16 in proximity were detected to be under-expressed. Among them, *CTCF* had a fold change of 0.66 (FDR=0·000021; unadjusted p-value= 1·5×10^-10^) and is associated with Intellectual developmental disorder, autosomal dominant 21 (OMIM# 615502), where also called syndromic intellectual disability in ClinGen curation database. This syndrome is characterized by developmental delay (commonly presenting motor and speech delay) with intellectual disability. Other features include microcephaly, scoliosis, hypotonia, eye anomalies, attention deficit hyperactivity disorder and bicuspid aortic valve.^31,32^ While clinical phenotypes are highly compatible with the patient phenotypes, hypotonia might partially explain muscle phenotypes of the patient and further understanding will be required to address patient’s other muscular phenotypes. Previous cell and animal model suggested the role of *CTCF* in myogenesis.^33^  To identify the variant, trio WGS was subsequently performed for this family and identified a ~280kb *de novo* NC_000016.10: g. (67429006_67710074) del in the proband, overlapping with the 5 under-expressed genes. This deletion covers the entire *CTCF* gene, supporting the almost halved expression detected from RNA-seq results. As *CTCF* is curated to be haploinsufficient in ClinGen, this deletion is classified as pathogenic with 1 point for copy number variant (CNV) loss based on the ACMG/AMP copy number variant (CNV) guideline^34^. No other likely variants were identified from myopathy-related panels in WGS that might further explain the muscle phenotypes of the patient.


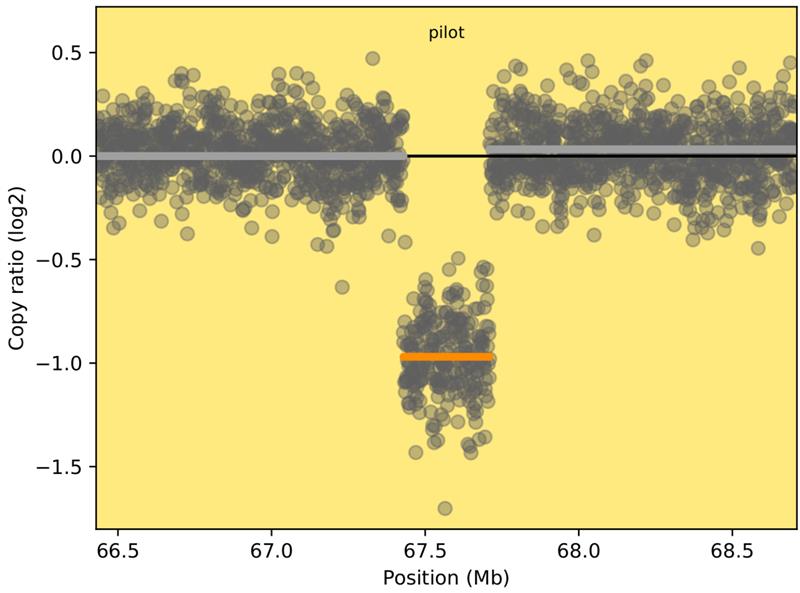


*B84: This figure shows a decreased copy ratio of GRCh38 chr16: 67429006-67710074. A value of copy ratio (log2) indicates a heterozygous deletion of this chromosomal region.*

**B105**

This patient was clinically diagnosed of neurofibromatosis with distinctive features including multiple café au lait, plexiform neurofibroma, axillary and inguinal freckles. The patient also had lisch nodule, hamartomata in brainstem and minimal increase in size of multiple brain regions. Previous NF1 genetic test was shown to be negative and therefore this individual was recruited for our RNA-seq study.

Our RNA-seq analysis detected *NF1* as a significant aberrant splicing outlier (|𝛥J| =0·32; FDR=0·021; unadjusted p-value= 4·1×10^-7^). *NF1* is a well-known gene that is associated with neurofibromatosis type 1 (OMIM# 162200) in autosomal dominant inheritance. Manual inspection showed a heterozygous skipping of exon 38 (341bp) leading to a frameshift, introducing a potential premature termination codon in exon 39 and predicted to undergo NMD. However, our expression results showed no significant results (Fold change = 0·81; unadjusted p-value= 0·023; FDR=1) with only a certain degree of NMD, which potentially indicate possible incomplete NMD. This patient was then recruited for trio WGS and subsequently identified a heterozygous deletion: NC_000017.11:g.31327741_31329914delinsT (NM_001042492.3:c.5511_5610-382delinsT, r.5269_5609del, p.(Gly1758Serfs*4)), occurred *de novo* in the proband. This deletion led to the loss of splice donor in exon 38 that results in the skipping of the exon. The RNA-seq data showing incomplete NMD lowered the PVS1 criteria from PVS1 to PVS1_moderate. With *de novo* occurrence, phenotypic specificity and absence in general population, this variant is classified as pathogenic (ACMG/AMP^4–6^:PVS1_mod(RNA), PS2, PP4_strong and PM2_supporting). While RNA-seq did not downgrade the variant in this case, accurate understanding of events in patient are important as opposed to relying on *in silico* predictions, especially in cases where the probability of variant pathogenicity is marginal.


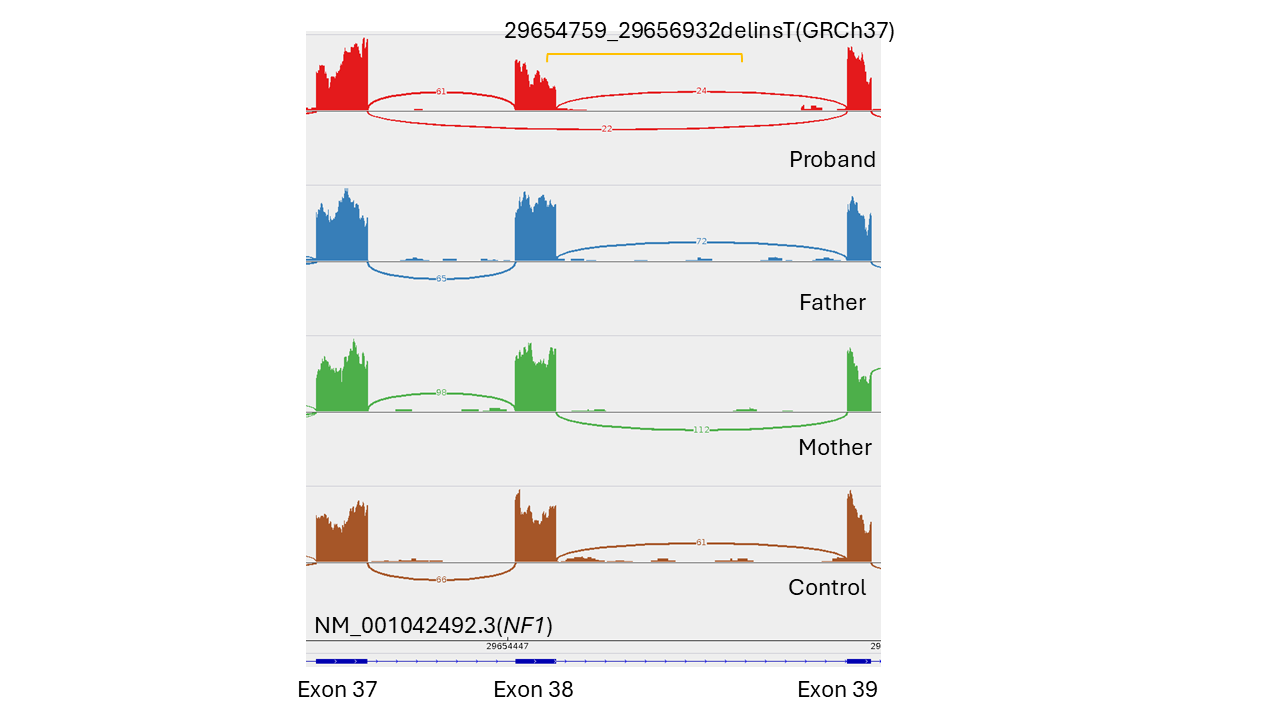


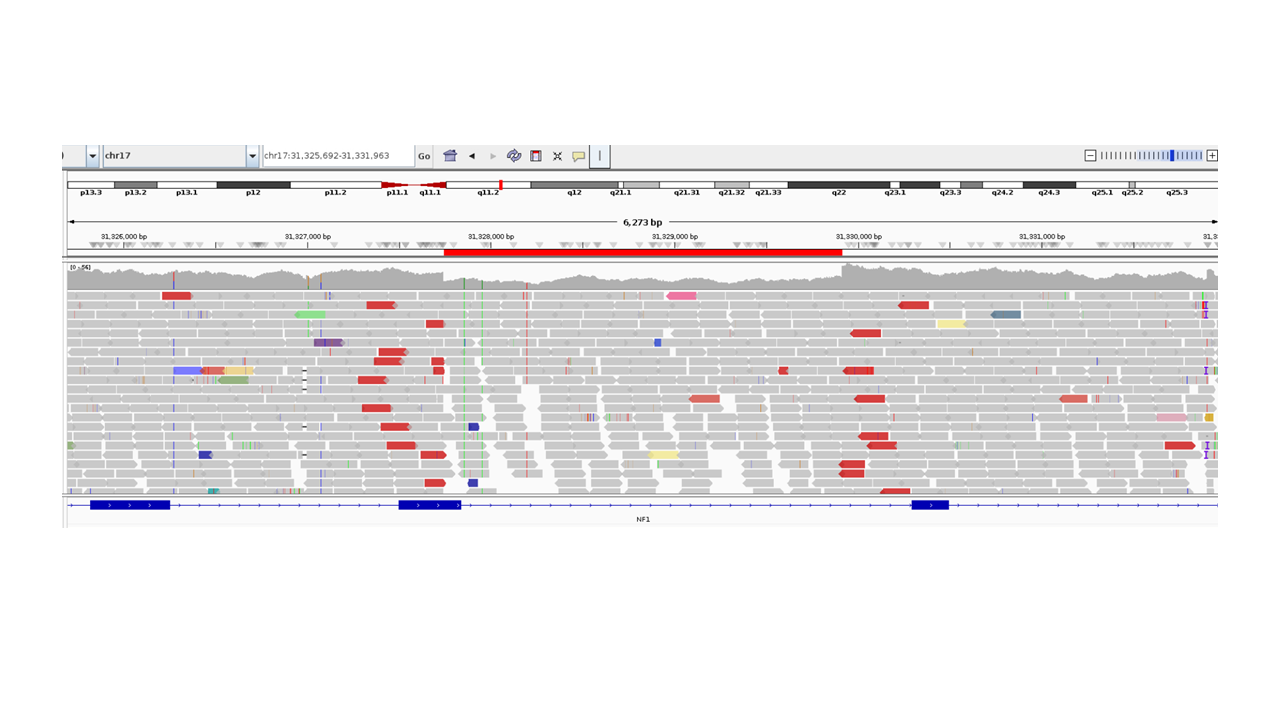


*B105 (IGV): For the RNA-seq results, compared to father, mother and control, proband carrying the indel variant demonstrates a clear event of exon 38 skipping (upper). Looking at the WGS results (lower), the decreased coverage with explicit boundaries indicated a deletion. The deleted region is indicated by the red bar at the top.*

**B107**

This participant was clinically diagnosed of neurofibromatosis with distinctive features of multiple café au lait spots, axillary freckles, lisch nodules. Plexiform neurofibroma was found in cervical spine with other clinical features including combined attention deficit hyperactivity disorder, left facial venous malformation and frequent epistaxis. The patient remained to be genetically undiagnosed despite previous next generation sequencing and multiplex ligation-dependent probe amplification methods, who was then recruited for RNA-seq analysis.

RNA-seq results detected *NF1* as significant aberrant splicing outlier (|𝛥J| =0·56; FDR=0·0046; unadjusted p-value=8·5×10^-8^). Further manual inspection revealed a heterozygous NM_001042492.3:c.2410-16A>G, r.2409_2410ins[2410-15_2410-1], p.(Gln803_Ala804ins*5) variant, suspected to be *de novo*. *NF1* is a well-known gene that is associated with neurofibromatosis type 1 (OMIM# 162200) in autosomal dominant inheritance. The presence and *de novo* inheritance of the variant is further confirmed by trio WGS. This variant is a well-reported pathogenic variant (ClinVar VCV000572474.22). RNA-seq results showed a heterozygous 15bp exon elongation of exon 21, which was also absent in the parents, leading to an in-frame insertion. Despite being in-frame, the insertion included a premature termination codon, which renders the transcript to be predicted to undergo NMD. However, our expression analysis showed no significant reduction of *NF1* expression (Fold change= 0·89; unadjusted p-value=0·20; FDR=1). Absence of indication of NMD from our RNA-seq data lowered the applicable strength of PVS1 in this case from very strong to strong. Along with the absence of variant in the general population, phenotypic specificity, occurrence in *de novo*, this variant is classified as Pathogenic (ACMG/AMP^4–6^:PVS1_strong(RNA), PM2_supporting, PP4_strong and PS2). Without RNA-seq results, PP3 will be used instead based on spliceAI^3^ predictions.


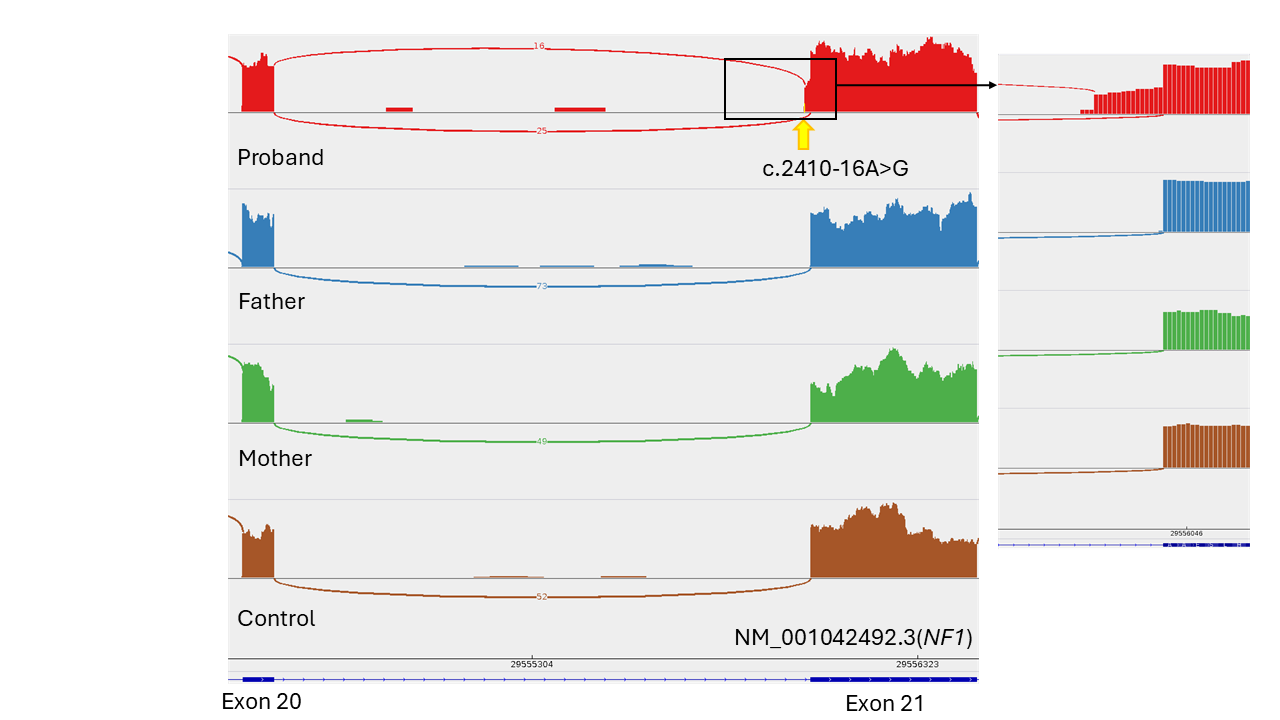


*B107 (RNA-seq IGV): Proband, with c.2410-16A>G, shows 15bp exon elongation of exon 21, which is absent in non-carriers (i.e. father, mother and control).*

**B22**

This patient had suspected mitochondrial disease/neurodegenerative disease, presenting phenotypes including developmental delay, infantile onset hypotonia with progressive deterioration in walking and cognition, proximal muscle weakness, left limb spasticity and ataxia. Bilateral hearing impairment, swallowing problem and borderline intelligence were also reported. He had an elder sister, who has deceased, suffered from similar and more severe phenotypes. With no potentially relevant variant identified from exome analysis, this patient was subsequently referred for RNA-seq analysis.

Our RNA-seq results identified *PSMF1* as both an aberrant expression (fold change = 0·23; FDR = 0·020; unadjusted p-value= 1·3×10^-7^) and splicing outlier (|𝛥J| = 0·44; FDR = 8·3 × 10^-24^; unadjusted p-value=8·8×10^-30^). There was no gene-disease association reported for *PSMF1*, but it has been reported to be involved in proteosome transport in axons important for neuronal function and synaptic development,^35^  which might potentially underlie neurological disorders when its function is disrupted. This further guided the identification of NM_006814.4:c.282+5G>A, r.[282_283ins[gugaa;282+6_282+17],283_365del,?], p.[(Leu94_Glu95ins*7), (Glu95Aspfs*6),?] in homozygous form from the RNA-seq data and exome reanalysis, in which the same variant is also found homozygous in his sister and heterozygous in parents. Manual inspection of RNA-seq results showed 17bp exon 2 elongation (majority) with other minor aberrant transcripts with different lengths of exon elongation and exon 3 skipping. A majority of the transcripts causes frameshift and subsequently lead to NMD, which is supported by our significant expression result and suggestive of loss of function disease mechanism. This finding led to further collaboration with international research team, resulting in a total of 24 affected individuals. Further investigation was performed including *in silico* modelling, RNA-seq analysis for other patients, minigene assay, etc. leading to the establishment of association between *PSMF1* and a neurodegenerative syndrome with movement disorders in autosomal recessive inheritance.^36^


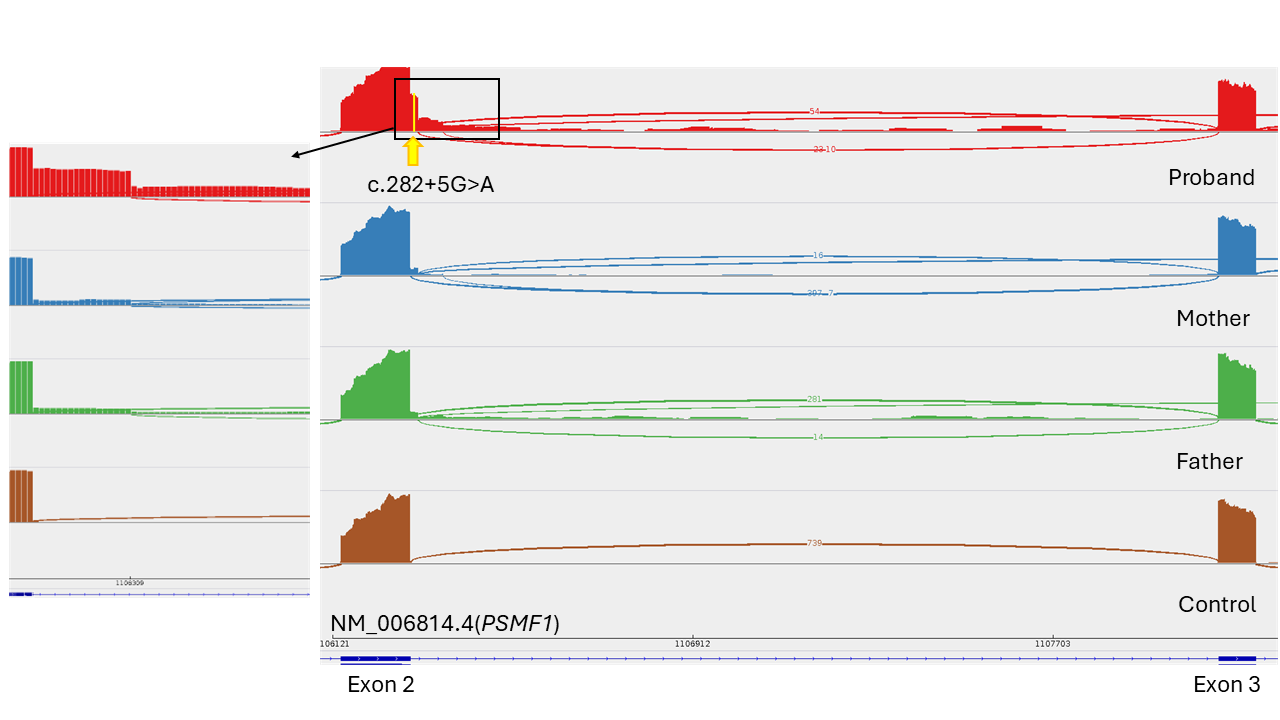


*B22 (RNA-seq IGV): Compared with non-carriers (mother, father and control), proband shows a majority of aberrant transcripts with 17bp exon 2 elongation, with other minor transcripts showing different lengths of exon elongation and exon 3 skipping.*

**B31 (with B66 and B67)**

This patient was presented with global developmental delay with severe intellectual disability, generalized hypotonia, dyskinesia, dysphagia with oromotor delay and abnormal brain MRI results. He was also found to have short stature with relative macrocephaly, dysmorphic features, truncal obesity bilateral clinodactyly of hands, hallux valgus and convergent squint with fleeting eye contact. With negative results from previous array Comparative Genomic Hybridization and WES, this patient was referred for RNA-seq analysis.

RNA-seq results revealed 106 significant aberrant splicing outlier genes with FDR<0·1, which is significantly more than other internal controls and external GTEx controls (Z-score = 4·8, Student T two-tailed p-value = 4·7×10^-6^). This guided exome reanalysis and identified a *de novo* heterozygous NM_004640.7 (*DDX39B*): c.368G>A, r.368g>a, p.(Arg123Gln) variant. *DDX39B* is not detected as a splicing nor expression outlier with an expression fold change of 1·1 (unadjusted p-value = 0·59, FDR=1). There was no gene-disease association for *DDX39B*, but the role of the gene has prompted further investigation. *DDX39B* is involved in RNA processing as a member of ATP-dependent RNA helicases (DEAD-box family) and TREX-complex affecting transcription, export and splicing processes.^37,38^ Disruption of normal function of this gene likely leads to the exceptional amount of aberrant splice events in the proband. This led to further collaboration with international research team and collectively recruited 6 individuals with *DDX39B* variants. Further investigation was performed including clinical phenotyping, *in silico* modelling, co-immunoprecipitation and protein expression assays and animal model studies. Global transcriptomics analysis was further performed for **B66** and **B67**, revealing similar RNA phenotype as **B31** proband. **B66,** carrying *de novo* NM_004640.7: c.109G>T, r.109g>u, p.(Gly37Cys), had 303 genes aberrantly spliced with FDR<0·1 ; **B67**, with maternally inherited heterozygous NM_004640.7: c.433-1G>T, r.433_465del, p.(Val145_Lys155del), had 297 genes aberrantly spliced with FDR<0·1. Both are significantly more than the control cohort (**B66:** Z-score=14·9, Student T two-tailed p-value = 2·4×10^-29^; **B67**: Z-score=14·6, Student T two-tailed p-value =1·2×10^-28^). Furthermore, while *DDX39B* is also not detected as a splicing nor expression outlier (fold change=0·96, unadjusted p-value=0·71, FDR=1) in **B66,** RNA-seq analysis of **B67** detected *DDX39B* as aberrant splicing outlier (|𝛥J| = 0·48; FDR=1·8 × 10^−10^; unadjusted p-value=3·3× 10^-15^), showing an in-frame deletion of 33bp in exon 5. The RNA-seq finding conformed with heterozygosity and is not predicted to undergo NMD, which is also supported by our RNA-seq insignificant expression results (fold change = 1·1, unadjusted p-value=0·35, FDR=1). This finding supported the pathogenicity of the splice variant in **B67**. The aberrant global transcriptomics signature (445 genes aberrantly spliced; Z-score=22·3; Student T two-tailed p-value=8·5×10^-45^) and *DDX39B* as a significant splicing outlier (| 𝛥J| = 0·57; FDR=3·2 × 10^−13^; unadjusted p-value=3·9×10^-18^) are also detected in the proband’s mother. Among the 950 aberrantly spliced genes, only 34 were found in three or more individuals, with a limited consistency in splice loci, even between B67 and the proband’s mother. This finding supported that the mis-splicing event is non-specific.^39^  Global transcriptomics enabled the detection of potential RNA signature and supported the establishment of gene-disease association between *DDX39B* with *TREX*-complex-related neurodevelopmental syndrome.^39^


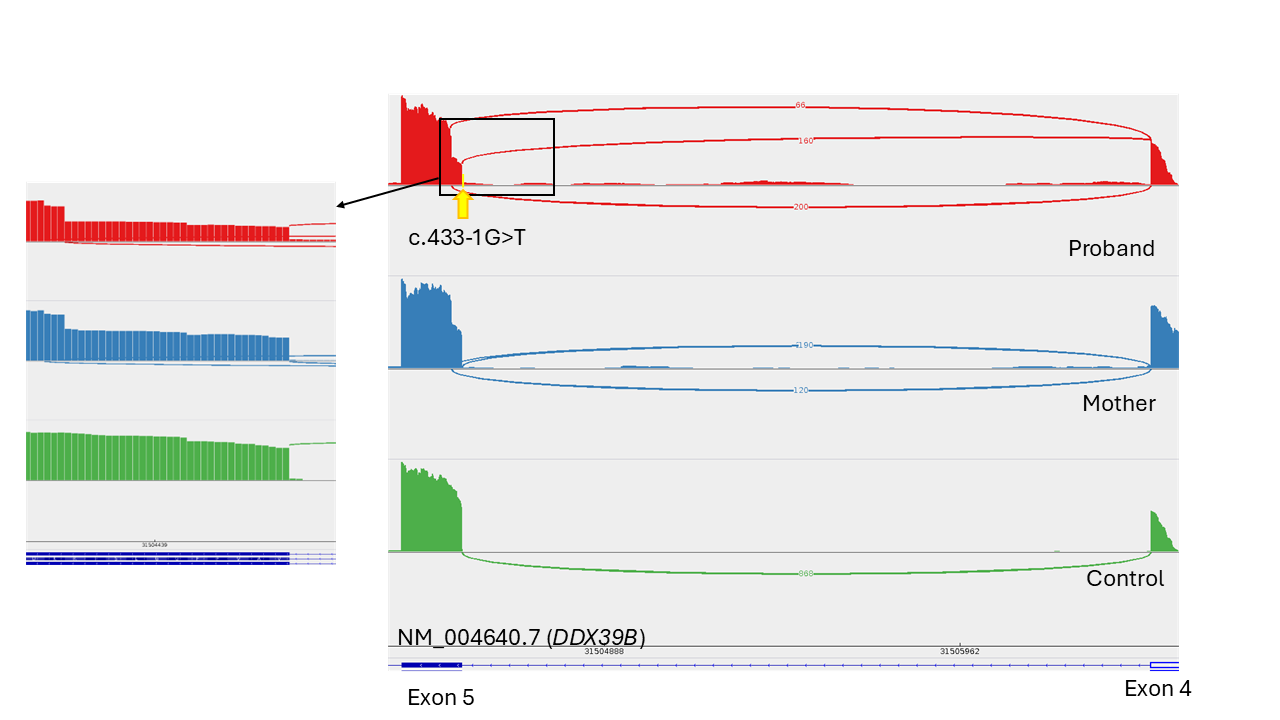


*B67(RNA-seq IGV): B67 proband and mother both carry the splice variant c.433-1G>T, hence showing an in-frame deletion of 33bp in exon 5, which is expectedly absent in the non-carrier control.*

**B93**

This patient had significant global developmental delay and autism spectrum disorder. Multiple family members are also presented with autism spectrum disorder. WGS analyses were negative for developmental disorder gene panels and therefore this patient was referred for RNA-seq analysis.

RNA-seq results detected *HECTD4* as an aberrant expression outlier (fold change = 0·51; FDR = 0·013; unadjusted p-value=1·5× 10^−7^). *HECTD4* is associated with Neurodevelopmental disorder with seizures, spasticity, and complete or partial agenesis of the corpus callosum (OMIM# 620250) with autosomal recessive inheritance. Loss of function and missense variants are mainly found in affected patients, who commonly present global developmental delay with intellectual disability, dysmorphism, seizures and partial or complete agenesis of corpus callosum.^40^ While no significant pathogenic variant found in *HECTD4,* long-read WGS was subsequently performed for this patient and identified a heterozygous expansion of GCC repeat (>1000bp) in the 5’UTR of *HECTD4*, inherited from mother with a relatively shorter expansion (~1kb) in one allele. Multiple lengths of expansion of the affected haplotype were identified in the proband, which might be attributed to “somatic instability” of the repeated region resulting in both expansion and contraction alleles after inheriting the ~1kb allele from mother.^41^  Methylation analysis showed hypermethylation in the expanded allele likely leading to silenced expression and potentially explaining the reduction in RNA-seq expression results. The establishment of diagnosis cannot be made at this point as no second pathogenic variant *in trans* with this event has been identified. However, considering the lower severity of phenotypes in this patient compared with reported patients, we suspected that haploinsufficiency of *HECTD4* might lead to milder forms of associated disorder. This gene has been scored as “high confidence” in SFARI gene, a database with genes implicated to be associated with autism spectrum disorder.^42^ This is further supported by the identification of *de novo*/heterozygous loss-of-function variants in patients with ASD,^43,44^  where this gene has also a probability of loss-of-function intolerance of 1 in gnomAD.^8^  Considering other potential developmental disorder genes have been excluded from WGS analysis, the clinical relevance of this mutation is highly suspected. Regardless, further investigations would be required to fully understand and establish the gene-disease association, starting from recruiting more patients, clinical phenotyping, *in vitro* cellular model knock-out assays, animal model studies, eSTR analysis investigating the association between repeat expansion and gene expression etc.


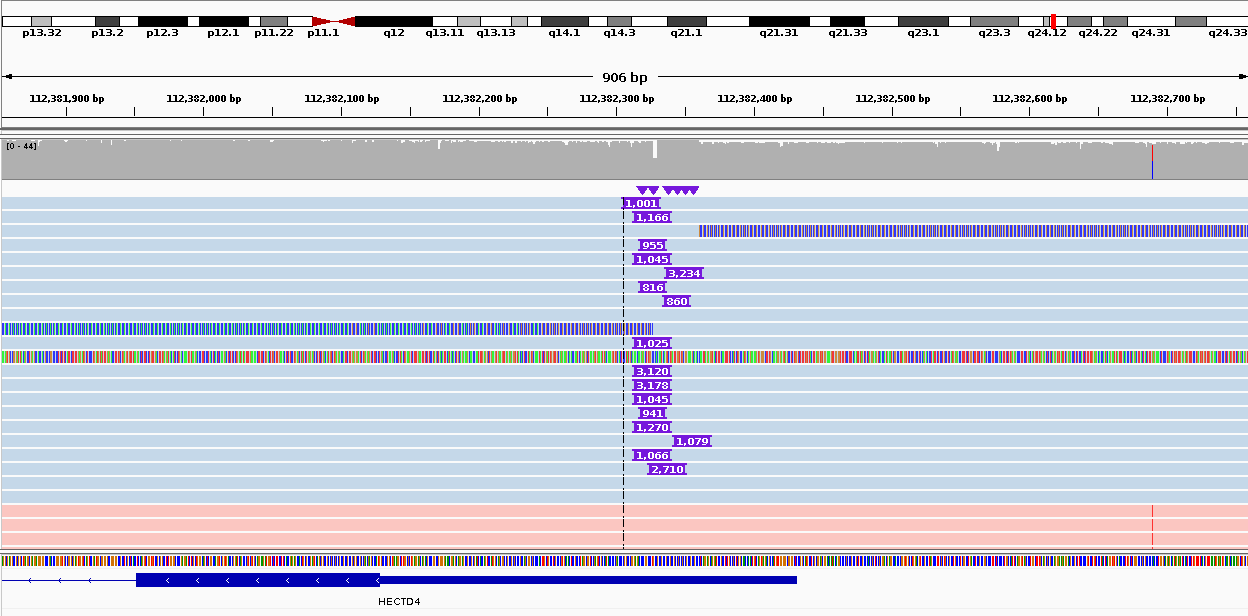


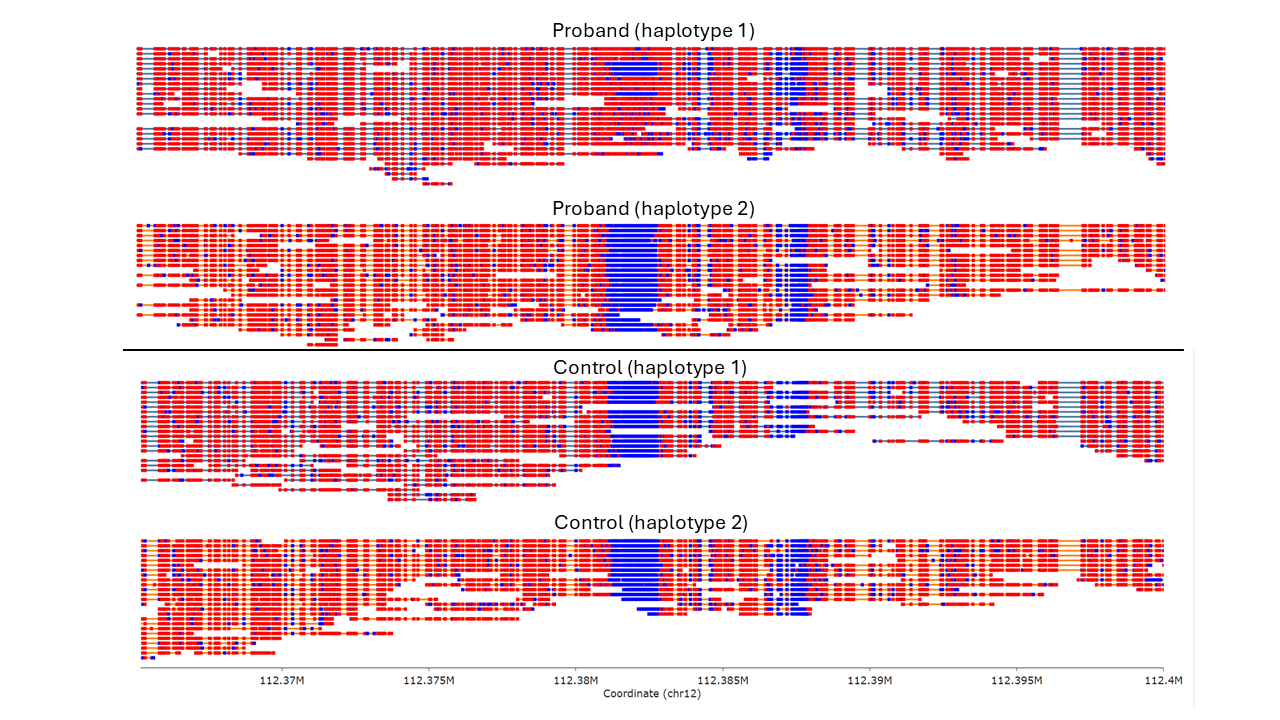


*B93: Long-read WGS results showing GCC expansion in the proband (upper), with blue reads representing haplotype 1 and pink reads representing haplotype 2. Most of the haplotype 2 carry GCC expansion indicated by the purple box, with a number showing the length of the repeat. Long-read WGS results from proband and an internal control (lower). Blue indicates unmethylated while red indicates methylated region. In control (i.e. without GCC expansion) both haplotypes are unmethylated. However, in proband (haplotype 1), the GCC expansion region is hypermethylated.*

**B114**

This patient was presented with borderline microcephaly, soft dysmorphism, moderate intellectual disability and bilateral sensorineural hearing loss. Genitalia abnormalities including small penis and undescended right testis, and vascular malformation (small patent ductus arteriosus, dilated main pulmonary artery and branched pulmonary arteries, and dilated left atrium and ventricle) are also found in patient. RNA-seq analysis was performed for this patient.

RNA-seq result detected *WDR11* as an expression outlier (fold change=0·55; FDR = 0·0014; unadjusted p-value= 1·9×10^-7^). *WDR11* is associated with two different diseases: 1) Hypogonadotropic hypogonadism 14 with or without anosmia (OMIM# 614858) in autosomal dominant inheritance, characterized by pubertal failure. Reported patients mainly carry missense variants. 2) Intellectual developmental disorder, autosomal recessive 78 (OMIM# 620237), characterized by microcephaly, mild short stature and intellectual disability. Loss-of-function variants were found in affected individuals. ^45,46^ There is currently limited understanding of the association between *WDR11* and human disorders. Heterozygous variants found to cause autosomal dominant congenital hypogonadotropic hypogonadism has also been reported to cause primary hypogonadism.^47^ While a cohort with biallelic *WDR11* variants found patients and parents carrying heterozygous loss-of-function variants are not reported to have hypogonadism-related phenotypes.^45^ A case with loss-of-function heterozygous *WDR11* variant has also been reported to have normal neurological development but abnormal genital and olfactory phenotypes.^48^ Our proband is presented with phenotypes overlapping the two diseases, suggesting possible expansion of phenotypic spectrum of *WDR11*-related diseases. This prompted duo WGS analysis for the patient and found a heterozygous NC_000010.11:g.(119969782_133655950)del variant that is not found in the father, classified as likely pathogenic (score 0.9 for CNV loss)^34^ .This is supported by our RNA-seq data where 26 expression outliers within this region are detected with a fold change ranging from 0·32-0·61 (FDR<0·1). This deletion contained *WDR11* and none of the remaining genes within the deletion are likely explaining patient phenotypes. WGS analysis found no other positive findings from gene panels related to patient’s phenotypes. Further studies including recruiting similarly affected patients, clinical phenotyping, *in vitro* gene-knock out assays, animal models, RNA-level correlation with phenotypic spectrum etc. would be required to further understand and pinpoint the underlying clinically relevant gene for this patient and further investigate on *WDR11* in human diseases.


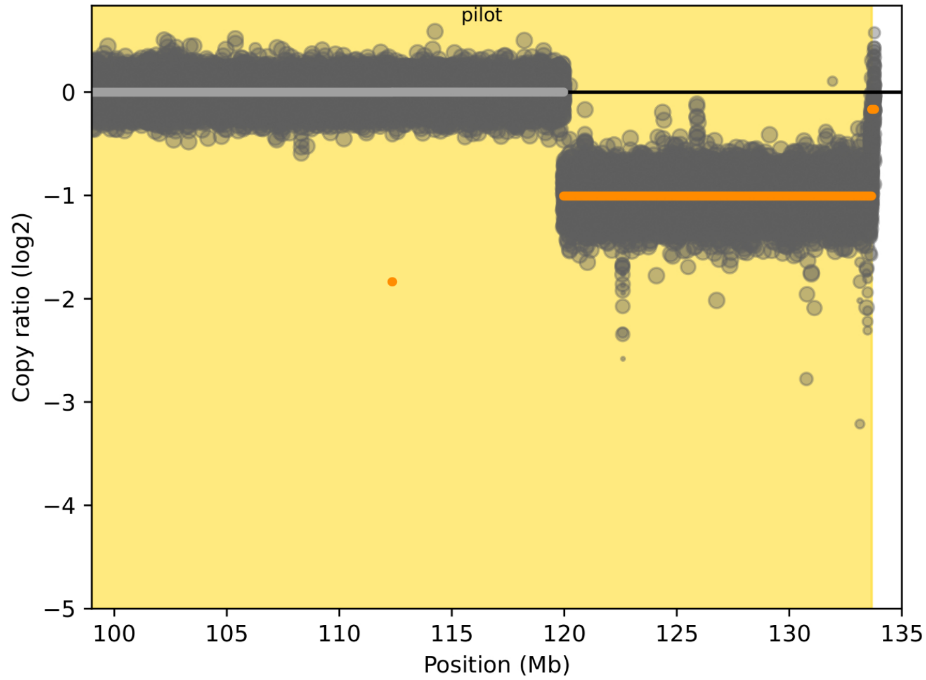


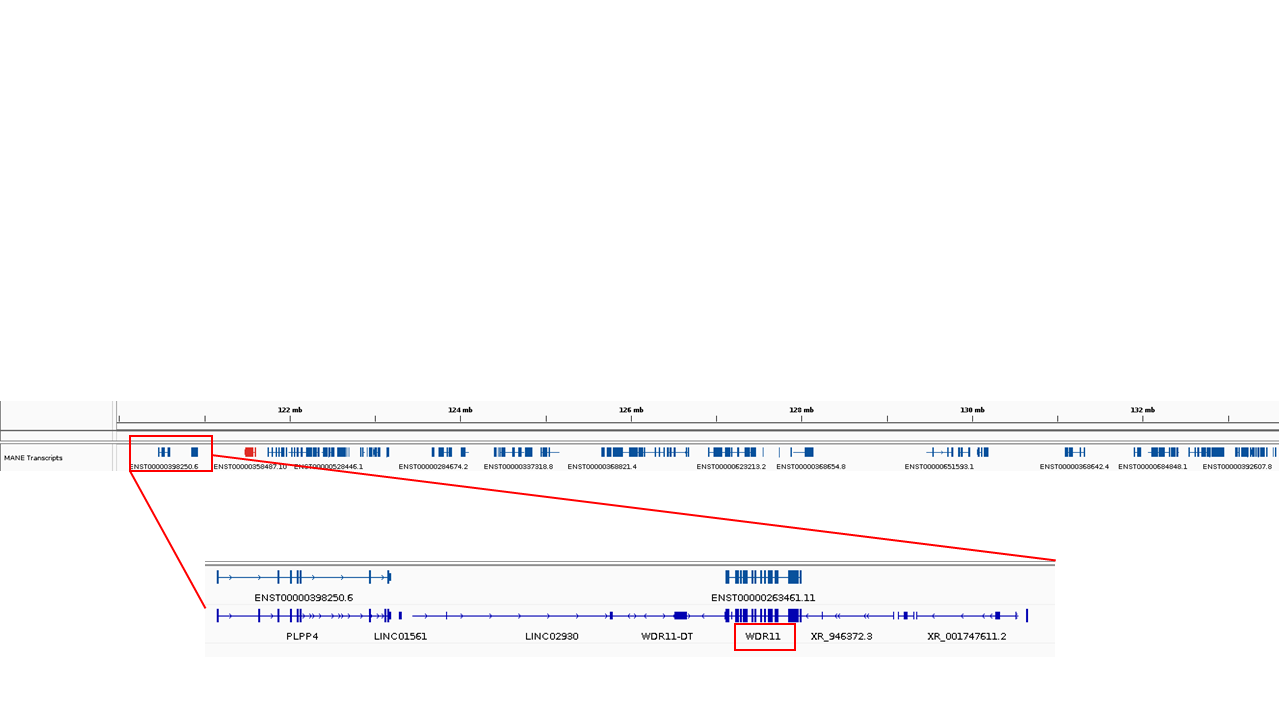


*B114: This figure shows a decreased copy ratio of GRCh38 chr10:119969782-133655950. A value of copy ratio (log2) indicates a heterozygous deletion of this chromosomal region. Genes (MANE transcripts) that are within the deleted region, in which includes WDR11 (lower).*

**Supplementary Information 3: Description of BF1 case aided by Fibroblast RNA-seq**

**BF1**

This patient was presented with global developmental delay with mild intellectual disability, leukoencephalopathy with mild progressive spasticity, liver parenchymal derangement with fibrosis and portal hypertension, progressive renal failure and protein-losing enteropathy, and suspected bilateral optic atrophy. Previous extensive investigations and WES were unrevealing and therefore this patient was referred for RNA-seq analysis.

Initial blood RNA-seq results were negative, but fibroblast RNA-seq results identified 26 histone genes as overexpression outliers controls (fold change=2·3-7·9; FDR= 2·9×10^-7^-0·054; unadjusted p-value= 1.8×10^-12^ -8.2×10^-6^). This transcriptomics signature guided the reanalysis of exome data and identified compound heterozygous NR_023317: n.[28C>T]; [35G>A] variants with no inheritance confirmed. *RNU7-1*, a non-coding RNA component of small nuclear ribonucleoproteins involved in mRNA processing, is associated with Aicardi-Goutières syndrome 9 (OMIM# 619487) in autosomal recessive inheritance.^49^  This disease is characterized by early-onset encephalopathy with intellectual disability.^50^  These variants have been reported in affected patients with similar RNA phenotype of misprocessed canonical histone transcripts and increased type I interferon response.^49^ Further clinical confirmation of stimulated type I interferon signalling in the patient (“inflammatory “interferon score = 18·6, decimalized age of 19·3 years), along with the highly specific histone transcript overexpression, enabled the application of PP4 at a strong level. This results in a classification of likely pathogenic for both variants, considering they are both rare in the general population (n.[28C>T] gnomAD v4.1.0= 0.00065; n.[35G>A] gnomAD v4.1.0 = 0.000050)^8^  and have been reported in other affected patients (ACMG/AMP^4–6^: PP4_strong, PM2_supporting, PM3). This case has previously been reported in our previous publication.^28^

**Supplementary table 1. Review of 18 RNA-seq Studies**

| **Paper** | **Cohort size (RNA-seq performed)** | **Disease** | **Tissue** | **Diagnostic Rate (%)^** | **Total Hypothesis-free cases (diagnosed + aided)** | **Total hypothesis-driven cases (diagnosed + aided)** | **Hypothesis-free Diagnosis** | **Hypothesis-driven diagnosis** | **Hypothesis-free**  **aided (no diagnosis)** | **Hypothesis-driven aided (no diagnosis)** |
| --- | --- | --- | --- | --- | --- | --- | --- | --- | --- | --- |
| Blake B, Brady LI, Rouse NA, Nagy P, Tarnopolsky MA. The Efficacy of Whole Genome Sequencing and RNA-Seq in the Diagnosis of Whole Exome Sequencing Negative Patients with Complex Neurological Phenotypes. J Pediatr Genet. 2023;12(3):206-12. | 20 cases | paediatric onset neurological phenotypes | Blood | Mainly through WGS, no additional diagnosis made through RNA-seq | 0 | 1 | 0 | 0 | 0 | 1 |
| Kremer LS, Bader DM, Mertes C, Kopajtich R, Pichler G, Iuso A, et al. Genetic diagnosis of Mendelian disorders via RNA sequencing. Nature Communications. 2017;8(1):15824 | 48 cases with unresolved ES | Mendelian disorders | Fibroblast | 5/48 (10·4%) | 3 | 2 | 3 | 2 | 0 | 0 |
| Riquin K, Isidor B, Mercier S, Nizon M, Colin E, Bonneau D, et al. Integrating RNA-Seq into genome sequencing workflow enhances the analysis of structural variants causing neurodevelopmental disorders. Journal of Medical Genetics. 2024;61(1):47. | 9 cases with unresolved GS | Neurodevelopmental disorders | fibroblast | 2/9 (22·2%) | 0 | 3 | 0 | 2 | 0 | 1 |
| Deshwar AR, Yuki KE, Hou H, Liang Y, Khan T, Celik A, et al. Trio RNA sequencing in a cohort of medically complex children. The American Journal of Human Genetics. 2023;110(5):895-900 | 39 cases | Rare genetic disorder | Blood | 3/39 (7·7%) | 3 | 1 | 3 | 0 | 0 | 1 |
| Cummings BB, Marshall JL, Tukiainen T, Lek M, Donkervoort S, Foley AR, et al. Improving genetic diagnosis in Mendelian disease with transcriptome sequencing. Sci Transl Med. 2017;9(386). | 50 cases | Muscle disorders | Muscle | 17/50 (34%) | 7 | 12 | 7 | 10 | 0 | 2 |
| Murdock DR, Dai H, Burrage LC, Rosenfeld JA, Ketkar S, Müller MF, et al. Transcriptome-directed analysis for Mendelian disease diagnosis overcomes limitations of conventional genomic testing. J Clin Invest. 2021;131(1). | 83 cases unresolved by ES/GS | Rare disease [Neurology(46%), Muscular disorders(21%), immune diseases (8%)] | Blood or Fibroblast | 14/83(16·9%) | 9 | 5 | 9 | 5 | 0 | 0 |
| Estévez-Arias B, Matalonga L, Yubero D, Polavarapu K, Codina A, Ortez C, et al. Phenotype-driven genomics enhance diagnosis in children with unresolved neuromuscular diseases. European Journal of Human Genetics. 2025;33(2):239-47. | 13 cases | unresolved neuromuscular diseases | Muscle | 3/13 (23·1%) | 0 | 3 | 0 | 3 | 0 | 0 |
| Lunke S, Bouffler SE, Patel CV, Sandaradura SA, Wilson M, Pinner J, et al. Integrated multi-omics for rapid rare disease diagnosis on a national scale. Nature Medicine. 2023;29(7):1681-91 | 115 cases | Diverse | Blood | 3/115 (2·6%) | 0 | 5 | 0 | 3 | 0 | 2 |
| Frésard L, Smail C, Ferraro NM, Teran NA, Li X, Smith KS, et al. Identification of rare-disease genes using blood transcriptome sequencing and large control cohorts. Nat Med. 2019;25(6):911-9. | 71 cases (unresolved prior to RNA-seq) | Rare diseases [Neurology(28%)] | Blood | 4/71 (5·6%) | 1 | 8 | 1 | 3 | 0 | 5 |
| Cloney T, Gallacher L, Pais LS, Tan NB, Yeung A, Stark Z, et al. Lessons learnt from multifaceted diagnostic approaches to the first 150 families in Victoria’s Undiagnosed Diseases Program. Journal of Medical Genetics. 2022;59(8):748 | 24 cases (GS and RNA-seq performed together) | Rare diseases | Fibroblast | 2/24 (8·3%) | 2 | 2 | 0 | 2 | 2 | 0 |
| Colin E, Duffourd Y, Tisserant E, Relator R, Bruel A-L, Tran Mau-Them F, et al. OMIXCARE: OMICS technologies solved about 33% of the patients with heterogeneous rare neuro-developmental disorders and negative exome sequencing results and identified 13% additional candidate variants. Frontiers in Cell and Developmental Biology. 2022;Volume 10 - 2022 | 19 cases (unresolved prior to RNA-seq) | Neurodevelopmental disorders | Blood or Fibroblast | 2/19 (10·5%) | 1 | 2 | 1 | 1 | 0 | 1 |
| Lee H, Huang AY, Wang L-k, Yoon AJ, Renteria G, Eskin A, et al. Diagnostic utility of transcriptome sequencing for rare Mendelian diseases. Genetics in Medicine. 2020;22(3):490-9. | 48 cases (unresolved prior to RNA-seq) | undiagnosed rare Mendelian diseases | Blood or Fibroblast or Muscle | 7/48 (14·6%) | 0 | 7 | 0 | 7 | 0 | 0 |
| Gonorazky HD, Naumenko S, Ramani AK, Nelakuditi V, Mashouri P, Wang P, et al. Expanding the Boundaries of RNA Sequencing as a Diagnostic Tool for Rare Mendelian Disease. Am J Hum Genet. 2019;104(3):466-83. | 25 cases (unresolved prior to RNA-seq) | Muscle disorders | Fibroblast or Muscle | 6/25 (24%) | 6 | 3 | 3 | 3 | 3 | 0 |
| Colin E, Duffourd Y, Chevarin M, Tisserant E, Verdez S, Paccaud J, et al. Stepwise use of genomics and transcriptomics technologies increases diagnostic yield in Mendelian disorders. Frontiers in Cell and Developmental Biology. 2023;Volume 11 - 2023 | 15 cases (GS and RNA-seq performed together) | Rare diseases | Blood or Fibroblast | 3/15 (20%) | 0 | 3 | 0 | 3 | 0 | 0 |
| Yépez VA, Gusic M, Kopajtich R, Mertes C, Smith NH, Alston CL, et al. Clinical implementation of RNA sequencing for Mendelian disease diagnostics. Genome Medicine. 2022;14(1):38. | 197 cases (unresolved prior to RNA-seq) | mitochondrial disease | fibroblast | 18/197 (9·1%) | 18 | 25 | 5 | 13 | 13 | 12 |
| Jaramillo Oquendo C, Wai HA, Rich WI, Bunyan DJ, Thomas NS, Hunt D, et al. Identification of diagnostic candidates in Mendelian disorders using an RNA sequencing-centric approach. Genome Medicine. 2024;16(1):110. | 86 cases | Suspected mendelian disorder | Blood | 18/86(20·9%) | 3 | 15 | 3 | 15 | 0 | 0 |
| Marchant RG, Bryen SJ, Bahlo M, Cairns A, Chao KR, Corbett A, et al. Genome and RNA sequencing boost neuromuscular diagnoses to 62% from 34% with exome sequencing alone. Annals of Clinical and Translational Neurology. 2024;11(5):1250-66 | 53 cases selected post-exome (performed with other investigation and tailored for each case) | Monogenic neuromuscular disorders | Muscle | 34/53 (64·2%) | 1 | 40 | 0 | 34 | 1 | 6 |
| Luo X, Xiao B, Liang L, Zhang K, Xu T, Liu H, et al. Blood RNA-seq in rare disease diagnostics: a comparative study of cases with and without candidate variants. Journal of Translational Medicine. 2025;23(1):586. | 121 cases | Suspected mendelian disorder | Blood | 9/121 (7·4%) | 3 | 10 | 3 | 6 | 0 | 4 |

|  |  |  |  | **Total** | 57 | 147 | 38 | 112 | 19 | 35 |
| --- | --- | --- | --- | --- | --- | --- | --- | --- | --- | --- |

^ diagnostic rates only included cases either classified as hypothesis-free diagnosis or hypothesis-driven diagnosis

* classification of hypothesis-free/driven and diagnosed/aided are the same as that in the manuscript

Remark: The minimum diagnostic yield is defined as the smallest diagnostic rate that is not equal to 0 (when calculating the range of diagnostic rate among these 18 studies).

**Supplementary table 2. Details of Control Data from GTEx**

|  | **Sample ID** | **Sex** |  |  | **Sample ID** | **Sex** |  |  | **Sample ID** | **Sex** |
| --- | --- | --- | --- | --- | --- | --- | --- | --- | --- | --- |
| 1 | SRR1311062 | female |  | 21 | SRR1351166 | female |  | 41 | SRR1398183 | female |
| 2 | SRR1312598 | male |  | 22 | SRR1351836 | male |  | 42 | SRR1405437 | male |
| 3 | SRR1314113 | female |  | 23 | SRR1354138 | female |  | 43 | SRR1412987 | male |
| 4 | SRR1317832 | male |  | 24 | SRR1354291 | male |  | 44 | SRR1440137 | male |
| 5 | SRR1318005 | male |  | 25 | SRR1354665 | male |  | 45 | SRR1440421 | male |
| 6 | SRR1318605 | female |  | 26 | SRR1356248 | female |  | 46 | SRR1444852 | male |
| 7 | SRR1319772 | male |  | 27 | SRR1365873 | female |  | 47 | SRR1446244 | male |
| 8 | SRR1320137 | male |  | 28 | SRR1366813 | male |  | 48 | SRR1452631 | female |
| 9 | SRR1320403 | male |  | 29 | SRR1367499 | female |  | 49 | SRR1457397 | female |
| 10 | SRR1322619 | female |  | 30 | SRR1376294 | female |  | 50 | SRR1458706 | male |
| 11 | SRR1326471 | female |  | 31 | SRR1376619 | male |  | 51 | SRR1459739 | male |
| 12 | SRR1329047 | female |  | 32 | SRR1377714 | female |  | 52 | SRR1464618 | female |
| 13 | SRR1337540 | male |  | 33 | SRR1378026 | female |  | 53 | SRR1471331 | female |
| 14 | SRR1338055 | male |  | 34 | SRR1383855 | female |  | 54 | SRR1477149 | female |
| 15 | SRR1338648 | female |  | 35 | SRR1386967 | male |  | 55 | SRR1478722 | female |
| 16 | SRR1343501 | male |  | 36 | SRR1388081 | male |  | 56 | SRR1480789 | female |
| 17 | SRR1348294 | male |  | 37 | SRR1389133 | female |  | 57 | SRR1481351 | female |
| 18 | SRR1348468 | female |  | 38 | SRR1392072 | male |  | 58 | SRR1489116 | female |
| 19 | SRR1349032 | female |  | 39 | SRR1392478 | male |  | 59 | SRR1498313 | male |
| 20 | SRR1349276 | male |  | 40 | SRR1395509 | female |  | 60 | SRR1500170 | male |

**Supplementary Table 3. Clinical Information and Outcome for 102 Probands.**

| **Patient ID** | **Types of samples** | **Disease category** | **Clinical Phenotype** | **Outlier genes found to be associated with the phenotypes** | **Positive finding - help to make diagnosis** | **RNA-seq provide additional information for case interpretation** |
| --- | --- | --- | --- | --- | --- | --- |
| BF1^28^ | Blood/Fibroblast | Neurodevelopmental disorder | Mild ID, leukoencephalopathy, progressive hypotonia, liver derangement, renal failure | No significant finding in blood RNA-seq but 26 histone genes were identified as significant overexpression outliers (fold change = 2·3-7·9; FDR= 2·9×10^-7^-0.054; unadjusted p-value= 1.8×10^-12^ -8.2×10^-6^) in fibroblast RNA-seq | This transcriptomics signature in fibroblast RNA-seq directed the identification of likely pathogenic *RNU7-1* variants (NR_023317): n.[28C>T]; [35G>A] (*in trans*) in the non-protein coding region associated with Aicardi–Goutières syndrome through ES data, aligning with phenotypes in our patient |  |
| BF2 | Blood/Fibroblast | Neurodevelopmental disorder | GDD, infantile spasm, cortical visual impairment, movement disorders, lower and upper limb muscle wasting, neuropathic and myopathic changes, worsening of oromotor function, moderate oropharyngeal dysphagia | No significant finding |  |  |
| BF3 | Blood/Fibroblast | Neurodevelopmental disorder | GDD, moderate ID, endocrine dysfunction, short statue, initial central hypotonia evolving hypertonia and weakness, spasticity, muscle weakness, progressive, progressive dystonia, bilateral sensorineural deafness, bilateral undescended testes | No significant finding |  |  |
| BF4^28^ | Blood/Fibroblast | Suspected mitochondrial disease | GDD, severe ID, epileptic spasm, dystonia, bilateral hip subluxation, mildly decrease of mitochondrial complex I and IV activity in muscle, thoracic scoliosis, urination problem, recurrent urinary tract infection, swallowing difficulty, mild ptosis | Blood results: *GFM1* (fold change= 0·57; FDR = 0·016; unadjusted p-value=3·1×10^-7^) and *MFSD1* (fold change= 0·03; FDR = 1·1×10^-10^; unadjusted p-value=7·1×10^-16^) were detected as AE outliers.  Fibroblast results:  *GFM1* (fold change=0·35 ; FDR=4·5×10^-15^; unadjusted p-value=5.0×10^-20^) and *MSFD1* (fold change = 0·01; FDR=1·4×10^-21^; unadjusted p-value= 7.9×10^-27^) were detected as AE outliers. | *GFM1* and *MFSD1* are located in proximity in chromosome 3. *GFM1* is associated with combined oxidative phosphorylation deficiency 1. The detection of *GFM1* and *MFSD1* outliers guide the identification of a 104.5 kb deletion (chr3:158435847-158540316) (GRCh37) found to cover *MFSD1* and some candidate enhancers of *GFM1.* |  |
| B5 | Blood | Suspected mitochondrial disease | Developmental regression, progressive dystonia, movement disorder | No significant finding |  |  |
| B6 | Blood | Metabolic disorder | Methylmalonic acidaemia, SUCLA2 / SUCLG1 deficiency and cobalamin deficiency, high lactate and alanine, failure to thrive | No significant finding |  |  |
| B7 | Blood | Movement disorder | Cerebellar ataxia, oculomotor problem, episodic dyskinesia | No significant finding |  |  |
| BF8 | Blood/Fibroblast | Multisystem disorder | GDD, epilepsy, hearing impairment, visual impairment, duodenal atresia, mild hypertonic, hypertonia in wrist and ankles, dystonia/dyskinesia | No significant finding |  |  |
| B9 | Blood | Nil | Acute liver failure, methemoglobinemia, congenital diarrhoea | No significant finding |  |  |
| B10^1^ | Blood | Cardiac disease | Dilated Cardiomyopathy, suspected mitochondrial disorders, moderate ID with an intronic VUS variant *TAFAZZIN* (NM_000116.5) c.284+5G>A | *TAFAZZIN* was detected as a AS outlier (\|ΔJ\| = 0·5; FDR = 0·0000079; unadjusted p-value= 4.6×10^-11^) | RNA-seq result showed two exon elongation events that will cause immature termination of translation. This provide functional evidence to upgrade the hemizygous *TAFAZZIN* (NM_000116.5) c.284+5G>A from VUS to likely pathogenic. |  |
| B11^1^ | Blood | Cardiac disease | Hypertrophic obstructive cardiomyopathy, facial feature of Noonan syndrome with a VUS NM_006767.4 (*LZTR1*):c.1261-3C>G and likely pathogenic c.1943-256C>T variant *in trans* | Two aberrant splicing events were detected in *LZTR1* as AS outlier (\|ΔJ\| = 0·63, FDR = 0·00095, unadjusted p-value= 3.2×10^-8^; \|ΔJ \|= 0·38, FDR=0·032, unadjusted p-value=1.8×10^-7^) | *LZTR1* variants (NM_006767.4):c.1261-3C>G and c.1943-256C>T were found in the noncoding region and RNA-seq result showed that they will cause the aberrant splicing (including exon elongation, intron retention and cryptic exon) leading to immature termination. This confirmed the pathogenicity of c.1943-256C>T and provided functional evidence to upgrade c.1261-3C>G from VUS to likely pathogenic. |  |
| B12 | Blood | Congenital anomalies | Gross motor delay, verbal expression delay, brain and genitalia abnormalities and multiple ocular phenotypes with a VUS variant NM_138927.4 (*SON*): c.78-2A>C | *SON* was detected as AS outlier (\|ΔJ\| = 0·45; FDR = 0·00065; unadjusted p-value=3.1×10^-9^) |  | Three potential cryptic splice sites were suggested from *in silico* predictions. RNA-seq data showed aberrant splicing leading to in-frame deletion but the other potential cryptic splice sites leading to frameshift have not been used. The variant NM_138927.4 (*SON*): c.78-2A>C remained as VUS and cannot be upgraded, indicating that the association of this variant to the patient's phenotype is limited. |
| B13 | Blood | Immunodysregulatory disorder | Relapse hemophagocytic lymphohistiocytosis, prolonged fever, hepatosplenomegaly, lymphadenopathy | No significant finding |  |  |
| B14 | Blood | Immunodysregulatory disorder | Multifocal intracranial vasculitis / vasculopathy, Intermittent reticular rashes, Congenital abnormalities of great vessels, suspected deficiency of adenosine deaminase 2 | No significant finding |  |  |
| B22^36^ | Blood | Neurodegenerative disorder | Neurodegenerative disease, movement disorder | *PSMF1* (gene critical for neuronal function) was detected as both AE outlier (fold change = 0·23; FDR = 0·020; unadjusted p-value= 1·3×10^-7^) and AS outlier (\|ΔJ\| = 0·44; FDR = 8·3 × 10^-24^; unadjusted p-value=8·8×10^-30^) in the patient, likely to be associated with movement disorder. |  | The detection of *PSMF1* outlier guided the identification of homozygous variant (NM_006814.4:c.282+5G>A) in the patient and his deceased elder sister. RNA-seq data showed that it can lead to donor loss and exon elongation of *PSMF1* transcript. From GeneMatcher, we have contacted a research team in London who identified a few subjects with different *PSMF1* variants and neurodegeneration. This finding and collaboration led to a discovery of the *PSMF1* gene associated with neurodegeneration and movement disorders. |
| B23 | Blood | Metabolic disorder | Aromatic L-Amino Acid Decarboxylase (AADC) deficiency, GDD, movement disorder, recurrent oculogyric crises | No significant finding |  |  |
| B24 | Blood | Neurodevelopmental disorder | Moderate ID, ASD, obesity, facial dysmorphism | No significant finding |  |  |
| B25 | Blood | Movement disorder | Double hemiplegic pattern of spasticity, congenital hypothyroidism, GDD | No significant finding |  |  |
| B26 | Blood | Neurodevelopmental disorder | Mild to moderate GDD and facial dysmorphism | No significant finding |  |  |
| B27 | Blood | Neurodevelopmental disorder | Developmental delay, hypotonia, imperforate, multiple congenital anomalies | No significant finding |  |  |
| B28 | Blood | Neurodevelopmental disorder | GDD, ASD, microtia | No significant finding |  |  |
| B29 | Blood | Neurodevelopmental disorder | Suspected Sotos syndrome, macrocephaly, frontal bossing, history of developmental delay and some dysmorphism with *PTEN* VUS (NM000314.8: c.209+3A>T) | *PTEN* was detected as splicing outlier (\|ΔJ\| =0·55; FDR = 2·7×10^-11^;unadjusted p-value=2.8×10^-17^) | The RNA-seq result showed exon 3 skipping in *PTEN* transcript. This provided evidence to upgrade the *PTEN* VUS (NM000314.8: c.209+3A>T) previously identified in proband to likely pathogenic. |  |
| B30 | Blood | Neurodegenerative disorder | Neurodegeneration of brain iron accumulation, motor and learning problem, dizziness, | No significant finding |  |  |
| B31^39^ | Blood | Neurodevelopmental disorder | short stature, macrocephaly, multiple dysmorphic features, bilateral clinodactyly of hands, GDD, generalized hypotonia, dyskinesia and dysphagia | A large number of significant AS events in 106 genes with FDR<0·1 which is significantly larger (Z-score = 4·8, Student T two-tailed p-value =4·7×10^-6^)than other control and external GTEx controls. |  | This RNA-seq result suggests a generally disrupted transcriptomics profile which is functional consequence supporting the pathogenicity of the variant NM_004640.7 (*DDX39B*): c.368G>A p.(Arg123Gln) identified in the patient, encoding an RNA splicing factor. These finding directed a collaboration with overseas researchers and led to a discovery of the *DDX39B* gene associated with neurodevelopmental disorder. |
| B32 | Blood | Neurodevelopmental disorder | GDD, dystonia, titubation of head and trunk, microcephaly with subtle dysmorphic features, severe failure to thrive, delayed gastric emptying | No significant finding |  |  |
| B33 | Blood | Neurodevelopmental disorder | Atypical Rett syndrome, ASD, severe intellectual disability, dysmorphic features, febrile status epilepticus | No significant finding |  |  |
| B34 | Blood | Endocrine disorder | Multiple congenital anomalies, central hypothyroidism | No significant finding |  |  |
| B35 | Blood | Endocrine disorder | Leydig cell hypoplasia, Disorders of sex development (DSD) | No significant finding |  |  |
| B36 | Blood | Neurodevelopmental disorder | Mild GDD, mild ID, generalized dystonia | No significant finding |  |  |
| B37 | Blood | Neurodevelopmental disorder | Mild intellectual disability, subtle dysmorphism with Xq28 duplication | 14 genes on chromosomal Xq28 were detected as significant overexpression (fold change >2). |  | The RNA-seq result confirmed the duplication on chromosomal Xq28, and suggested that there is aberrant overexpression of the genes in this duplicated region. Four genes (*GDI1, RPL10, FAM50A, LAGE3*) are associated with X-linked intellectual developmental disorder and 3 are associated with dysmorphism. |
| BF38 | Blood/Fibroblast | Metabolic disorder | Mild carnitine uptake defect, with one SLC22A5 variant identified, Atypical febrile convulsion, ASD, bilateral undescended testes | No significant finding |  |  |
| B39 | Blood | Metabolic disorder | Probable fructose intolerance, history of nausea after fructose intake and voluntary fructose avoidance since infancy, multiple vitamin deficiencies | No significant finding |  |  |
| B40 | Blood | Metabolic disorder | Abnormal newborn screening of inborn errors of metabolism with elevated Phenylalanine | No significant finding |  |  |
| B41 | Blood | Cardiac disease | Dilated cardiomyopathy, biventricular failure | No significant finding |  |  |
| B42 | Blood | Suspected mitochondrial disease | GDD, progressive generalized dystonia, hyperlactataemia, bilateral basal ganglia lesions, suspected mitochondrial disease | No significant finding |  |  |
| B43 | Blood | Nil | Left mandibular odontogenic keratocyst with coarse facial features | No significant finding |  |  |
| B44 | Blood | Cardiac disease | Arrhythmogenic Right Ventricular Cardiomyopathy | No significant finding |  |  |
| B45 | Blood | Cardiac disease | Idiopathic ventricular fibrillation, risk of sudden death | No significant finding |  |  |
| B46 | Blood | Cardiac disease | sinus node dysfunction | No significant finding |  |  |
| B47 | Blood | Neurodevelopmental disorder | GDD, ASD, frontal bossing, drooling | *KMT5B* is detected as AS outlier (\|ΔJ\| = 0·44; FDR=0·057; unadjusted p-value=2·3×10^-6^) | The RNA-seq data showed a 5bp exon elongation and identified NM_017635.5 (*KMT5B*) :c.977+2T>A in the patient which is missed by exome sequencing. The variant is inherited from mother according to parents and variant confirmed through sanger sequencing. RNA-seq result rendered further confirmation of DNAm signature. These findings provided functional evidence for the curation of this variant as likely pathogenic. |  |
| B48^1^ | Blood | Cardiac disease | Dilated cardiomyopathy with a VUS variant NM_000256.3 (*MYBPC3*):c.1224-80G>A | RNA-seq data showed that cryptic acceptor site was created leading to aberrant splicing and expansion of exon 14 but *MYBPC3* is not detected as RNA splicing outlier. |  | RNA-seq data validated the SpliceAI prediction of the creation of cryptic acceptor site leading to expansion of exon 14. This also provided a more refined picture to a previous minigene splice experiment. However this evidence is not enough to upgrade the VUS NM_000256.3 (*MYBPC3*):c.1224-80G>A |
| BF49 | Blood/Fibroblast | Suspected mitochondrial disease | GDD, central hypotonia without other neurological signs, kreb cycle disorders, progressive ptosis, progressive myopathy, progressive cerebellar ataxia, fatiguability | No significant finding |  |  |
| B50 | Blood | Suspected mitochondrial disease | Suspected neurometabolic disease, infantile parkinsonism with generalized dystonia | No significant finding |  |  |
| B54 | Blood | Suspected mitochondrial disease | Developmental delay, suspected seizure, recurrent paroxysmal dystonia, nystagmus | ECHS1 expression is the lowest level among all other controls with fold change 0·64 but the gene is not detected as significant aberrant expression or splicing outlier. |  |  |
| B55 | Blood | Neurodevelopmental disorder | Developmental delay, cerebella ataxia, seizure, | No significant finding |  |  |
| B56 | Blood | Suspected mitochondrial disease | Developmental delay and movement disorders with an intronic *RARS2* VUS NM_020320.5:c.1238-28T>G identified compound heterozygous with another likely pathogenic *RARS2* variant c.685C>T | AS event is not detected in *RARS2* in terms of FDR, but it has considerable high magnitude of \|ΔJ\| =0·46 and smallest p-value in the cohort = 0·000016. | RNA-seq result demonstrated an activation of 2 cryptic splice acceptors in intron 14 leading to exon elongation with intron retention and both of the cryptic splice acceptors are predicted to cause NMD. These provide functional evidence to upgrade the deep intronic variant NM_020320.5 (*RARS2*): c.1238-28T>G from VUS to likely pathogenic. |  |
| B62 | Blood | Neurodevelopmental disorder | Tourette syndrome, ADHD, anxiety disorder, | No significant finding |  |  |
| B63 | Blood | Neurodevelopmental disorder | Microcephaly, epilepsy, squint, GDD, tremor | No significant finding |  |  |
| B64 | Blood | Neurodevelopmental disorder | Intractable epilepsy with cognitive decline | No significant finding |  |  |
| B65 | Blood | Epilepsy | Infantile epilepsy | No significant finding |  |  |
| B66^39^ | Blood | Neurodevelopmental disorder | GDD, hypotonia, feeding dysfunction, microcephaly, autistic behaviour and seizures | This case exhibited a substantial number of AS events in 303 genes with FDR<0·1 which is significantly larger (Z-score = 14·9, Student T two-tailed p-value = 2·4×10^-29^) than other control and external GTEx controls identified. |  | These outstanding numbers of AS events indicated that the transcriptomics profiles were disrupted, potentially due to the variant identified [NM_004640.7: c.109G>T, p.(Gly37Cys)] that helped to verify the function of *DDX39B* which is a gene with an unexplored function. |
| B67^39^ | Blood | Neurodevelopmental disorder | Short stature, epicanthic folds, ASD and features of ADHD, with delayed motor development and a history of seizures | This case exhibited a substantial number of significant AS events in 297 genes with FDR<0·1 which is significantly larger (Z-score = 14·6, Student T two-tailed p = 1.2×10^-28^) than other control and external GTEx controls identified.   AS: DDX39B (\|ΔJ\| = 0·48; FDR=1·8 × 10^−10^; unadjusted p-value=3·3× 10^-15^) |  | These outstanding numbers of AS events indicated that the transcriptomics profiles were disrupted, potentially due to the variant identified (NM_004640.7: c.433-1G>T) that helped to verify the function of *DDX39B* which is a gene with an unexplored function. Aberrant splice event (33bp deletion in exon 5) was also detected in DDX39B, supporting the pathogenicity of the splice variant. |
| B68 | Blood | Neurodevelopmental disorder | Hearing impairment, spastic diplegia, ID, bilateral hydronephrosis and dysmorphic features with a VUS variant NM_078629.4 (*MSL3*): c.749+5G>A | *MSL3* was detected as significant AS outlier (\|ΔJ\| = 0·95; FDR=7·9×10^-24^; unadjusted p-value=5.1×10^-29^) | RNA-seq data showed AS events (including exon elongation of 69bp, skipping of exon 7 and intron retention of intron 7) that leads to immature termination. This provided functional evidence to upgrade the hemizygous variant NM_078629.4 (*MSL3*):c.749+5G>A from VUS to likely pathogenic. |  |
| B69 | Blood | Cardiac disease | Infantile onset cardiomyopathy | No significant finding |  |  |
| BF70 | Blood/Fibroblast | Neurodevelopmental disorder | Intractable focal epilepsy, severe ID, spastic quadriplegia, no definite developmental progress, cortical visual impairment, generalized hypotonia | No significant finding |  |  |
| B71 | Blood | Congenital anomalies | Multiple congenital anomalies, big head, frontal bossing | No significant finding |  |  |
| BF73 | Blood/Fibroblast | Neurodevelopmental disorder | Microcephaly, intrauterine growth restriction, mild intellectual disability, dysmorphic features including mild ptosis and epicanthic fold, ASDs with inattention features, generalized dystonia, lower limb spasticity, intermittent drooling, mild hyperlipidaemia | No significant finding |  |  |
| B76 | Blood | Neurodevelopmental disorder | Premature birth with dystonic cerebral palsy , ID, epilepsy | No significant finding |  |  |
| B77 | Blood | Neurodevelopmental disorder | GDD, ADHD, mild to moderate sensorineural hearing impairment | No significant finding |  |  |
| BF78 | Blood/Fibroblast | Neurodevelopmental disorder | GDD, neonatal seizure, generalized dystonia with mild spasticity, extensive leukoencephalopathy, severe neonatal jaundice, rapid and very extensive involvement of white matter, leukodystrophy | No significant finding |  |  |
| B79 | Blood | Metabolic disorder | Hepatomegaly, suggestive of glycogen storage disease | No significant finding |  |  |
| B80 | Blood | Neurodevelopmental disorder | GDD, dysmorphism with a VUS *KMT2A* variant (NM_001197104.2: c.4480-18_4480del) | *KMT2A* was detected as AS outlier(\|ΔJ\| =0·47; FDR=0·00097; unadjusted p-value=1.6×10^-8^) |  | This finding helped the case interpretation by confirming that the *KMT2A* variant (NM_001197104.2: c.4480-18_4480del) caused a deletion of 3bp with no frameshift. This evidence supported that the variant will not cause immature termination and nonsense mediated decay, and its damaging effect is limited. Therefore, *KMT2A* variant should be classified as VUS |
| B81 | Blood | Neurodevelopmental disorder | Microcephaly, GDD, hypotonia, dystonia | No significant finding |  |  |
| B82 | Blood | Nil | Seizure, polycythaemia, prolong partial thromboplastin time, dysmorphism | No significant finding |  |  |
| B83 | Blood | Neurodevelopmental disorder | GDD, ID, microcephaly, dystonia | No significant finding |  |  |
| B84 | Blood | Neurodevelopmental disorder | Distal Myopathy, delay walking, easy falling | *CTCF* was detected as an AE outlier (fold change =0·66; FDR=0·000021; unadjusted p-value= 1.5×10^-10^) | RNA-seq identified the downregulation of 5 genes in the chromosome 16,with a confirmed ~ 280kb de novo deletion [NC_000016.10:g.(67429006_67710074)del] in WGS results. Apart from *CTCF*, other 4 genes including *CARMIl2*, *ATP6V0D1*, *ACD* and *RIPOR1* were detected to be AE outliers corresponding to the deleted region, all with a fold change of around 0.5. The heterozygous loss of *CTCF* is shown to be pathogenic and associated with "Intellectual developmental disorder" in autosomal dominant form, likely explaining the patient's phenotypes |  |
| B85 | Blood | Endocrine disorder | Hypothyroidism, hearing loss | No significant finding |  |  |
| B86 | Blood | Neurodevelopmental disorder | Dysmorphic features, ID, short stature | No significant finding |  |  |
| B87 | Blood | Neurodevelopmental disorder | Intractable epilepsy, GDD, autistic feature, elevation of urine sulfocyseine | no significant finding |  |  |
| B88 | Blood | Neurodevelopmental disorder | GDD, gross motor delay | no significant finding |  |  |
| B89 | Blood | Neurodevelopmental disorder | Orotic aciduria, GDD and ASD | No significant finding |  |  |
| B90 | Blood | Muscle diseases | Muscle spasm after exercise, feature of myopathic changes | No significant finding |  |  |
| B91 | Blood | Neurodegenerative disorder | Motor clumsiness, neurocognitive decline, hand stereotypies, suspected catatonia, sudden mood swing, suspected visual hallucination | No significant finding |  |  |
| B92 | Blood | Neurotransmitter disease | Severe bradykinesia, mild hand tremor, parkinsonism feature, limited bilateral eye abduction, GDD, neuromuscular condition, facial grimacing | No significant finding |  |  |
| B93 | Blood | Neurodevelopmental disorder | ASD and GDD | *HECTD4* was detected as AE outlier (fold change = 0·51; FDR = 0·013; unadjusted p-value=1·5× 10^-7^) |  | *HECTD4* is associated with Neurodevelopmental disorder with seizures, spasticity, and complete or partial agenesis of the corpus callosum, which likely matches with patient phenotypes. However, this disease has been reported in AR form only. No variant can be found through IGV. Long read GS results showed that there is expansion of GCC repeat of >1000bp in the 5' UTR and is hypermethylated. This might explain the drop of expression in RNA-seq. However no second pathogenic variant can be found. Therefore diagnosis cannot be established at this point. But this might suggest a possible previously unestablished disease mechanism where a milder form of the disorder is manifested in monoallelic form through epigenetic events. |
| B94 | Blood | Nephrotic disease | Steroid resistant nephrotic disease with end stage renal failure, short stature, recurrent diarrhoea with weight loss | No significant finding |  |  |
| B95 | Blood | Nil | Suspected Alagille syndrome, neonatal jaundice, mild hepatosplenomegaly, cholestasis, supravalvular pulmonary stenosis, secundum atrial septal defects | No significant finding |  |  |
| BF96 | Blood/Fibroblast | Neurodevelopmental disorder | Hyperbilirubinemia, ADHD, language delay, GDD mild to moderate ASD | No significant finding |  |  |
| B97 | Blood | Hearing disorder | Bilateral profound hearing loss, auditory neuropathy of both ears | No significant finding |  |  |
| B98 | Blood | Neurodevelopmental disorder | severe GDD, soft dysmorphism, hearing loss, left eye ptosis | No significant finding |  |  |
| B99 | Blood | Neurodevelopmental disorder | GDD, ASD, regression in speech, microcephaly, short stature | No significant finding |  |  |
| B100 | Blood | Neurodevelopmental disorder | Severe GDD, mild non-paralytic hypotonia, feeding problem, congenital brain malformation, bilateral absence facial nerve, infantile haemangioma, severe oropharyngeal dysphagia | No significant finding |  |  |
| B101 | Blood | Neurodevelopmental disorder | GDD, ASD, abnormal movement | No significant finding |  |  |
| B102 | Blood | Epilepsy | Atypical febrile and infebrile convulsion | No significant finding |  |  |
| B103 | Blood | Movement disorder | Cerebral palsy with spasticity and dyskinesia | No significant finding |  |  |
| B104 | Blood | Movement disorder | Symmetrically small, gross & find motor delay, weak in function | No significant finding |  |  |
| B105 | Blood | Multisystem disorder | Clinical Neurofibromatosis 1 | *NF1* detected as AS outlier (\|ΔJ\| =0·32; FDR=0·021; unadjusted p-value= 4·1×10^-7^) | RNA-seq data showed exon 38 skipping in the mane transcript (NM_001042492.3 ) of *NF1*. This guided the identification of a pathogenic heterozygous deletion [NC_000017.11:g. 31327741_31329914delinsT] in WGS. |  |
| B106 | Blood | Suspected mitochondrial disease | Neonatal onset severe episodic lactic acidosis, hypoglycaemia, cholestasis, pierre robin sequence, atypical haemolytic uraemic syndrome, renal impairment, hydrocephalus, large patent ductus arteriosus and atrial septal defect | No significant finding |  |  |
| B107 | Blood | Multisystem disorder | Suspected likely pathogenic variant in *NF1* gene | *NF1* detected as a splicing outlier (\|ΔJ\| =0·56; FDR=0·0046; unadjusted p-value=8·5×10^-8^) | RNA-seq data showed a 15bp exon elongation, which leads to a premature termination codon. A pathogenic NM_001042492.3 (*NF1*):c.2410-16A>G is identified from RNA-seq results , later confirmed in WGS. |  |
| B108 | Blood | Neurodevelopmental disorder | Dysmorphism, severe ID, Epilepsy, hearing impairment, bilateral cataracts | No significant finding |  |  |
| B109 | Blood | Epilepsy | infantile onset refractory focal epilepsy | No significant finding |  |  |
| B110 | Blood | Epilepsy | childhood absence epilepsy, low borderline IQ | No significant finding |  |  |
| B111 | Blood | Multisystem disorder | Christmas tree cataract, borderline GDD with mild fine motor delay | No significant finding |  |  |
| B112 | Blood | Epilepsy | recurrent atypical febrile convulsion | No significant finding |  |  |
| B113 | Blood | Neurodevelopmental disorder | Microcephaly, intrauterine growth restriction, pseudosquint, mild intellectual disability with speech delay, dysgenesis of the corpus callosum, dystonia, spasticity | No significant finding |  |  |
| B114 | Blood | Multisystem disorder | Moderate grade ID, soft dysmorphism including big ear and bilateral clinodactyly, small penis, undescended right testis, hearing loss, clinical sepsis at birth, cardiovascular anomalies including small patent ductus arteriosus, dilated main and branched pulmonary arteries, dilated left atrium and ventricle, pyloric stenosis and slight microcephaly | *WDR11* was detected as an AE outlier (fold change=0·55; FDR = 0·0014; unadjusted p-value= 1·9×10^-7^)  26 genes (including WDR11) within the chr10 deletion region (NC_000010.11:g.(119969782_133655950) del) are under expressed (FDR<0·1; FC=0·32-0·61) |  | The AD form is associated with hypogonadotropic hypogonadism with or without anosmia (missense variants, Loss of function), while the AR form is associated with intellectual developmental disorder (Loss of function variants). The patient's phenotype, however, is overlapping with both forms of the diseases, suggesting possible spectrum underlying the disorders associated with this gene. |
| B115 | Blood | Multisystem disorder | Significant delay, multiple dysmorphism including broad forehead, slight downslanting eyes, epicanthic fold positive, low set ears, small chin, protruding left lower lip, web neck, pectus excavatum, scoliosis, nonspecific chest pain | No significant finding |  |  |
| B116 | Blood | Neurodevelopmental disorder | significant GDD, ASD, macrocephaly, borderline ventriculomegaly | No significant finding |  |  |
| B117 | Blood | Cardiac disease | Tetralogy of Fallot, thyrotoxicosis, mild dysmorphism, tiny gallbladder polyp, prominent spleen | No significant finding |  |  |
| B118 | Blood | Neurological immune disorder | repeated parainfection autoimmune encephalitis, significant GDD, suspected genetic epilepsy syndrome | No significant finding |  |  |
| B119 | Blood | Neurodevelopmental disorder | GDD, hearing loss, soft dysmorphism with a VUS variant NM_133433.4(*NIPBL*):c.64+5G>C | *NIPBL* was detected as an AS outlier (\|ΔJ\| =0·22; FDR=0·0055; unadjusted p-value=2.6×10^-7^) which led to exon 2 skipping. | RNA-seq data showed exon 2 skipping in *NIPBL* transcript and provided functional evidence to upgrade NM_133433.4(*NIPBL*):c.64+5G>C from VUS to Likely Pathogenic |  |
| B120 | Blood | Neurodevelopmental disorder | GDD with high level of autism spectrum-related symptoms, suspected obstructive sleep apnoea | No significant finding |  |  |

AD: autosomal dominant; ADHD: attention deficit hyperactivity disorder; AE: aberrant expression; AR: autosomal recessive; AS: aberrant splicing; ASD: autism spectrum disorder; FDR: false discovery rate; GDD: global developmental delay; GS: genome sequencing; ID: Intellectual disability; UTR: untranslated region; VUS: variant of uncertain significance; WES: whole exome sequencing; WGS: whole genome sequencing; ΔJ: difference in Intron Jaccard Index

**Supplementary table 4:** **Cohort Demographics (sex-disaggregated data)**

| **Sex** | **Female (n=46)** | | **Male (n=56)** | |
| --- | --- | --- | --- | --- |
| **Analysis** |  |  | 25 | 44·6% |
| Singleton | 24 | 52·2% |  |  |
| Duos | 4 | 8·7% | 6 | 10·7% |
| Trio | 18 | 39·1% | 24 | 42·9% |
| Quadro | 0 | 0% | 1 | 1·8% |
| **Age**  **Mean (SD)** | **9.9 (6.4)** |  | **9.3 (6.7)** |  |
| ≤18yo | 41 | 89·1% | 50 | 89·3% |
| >18yo | 5 | 10·9% | 6 | 10·7% |
| **Disease Categories** |  |  |  |  |
| Central nervous system^ | 28 | 60·9% | 31 | 55·4% |
| Suspected mitochondrial diseases | 4 | 8·7% | 4 | 7·1% |
| cardiac diseases | 2 | 4·3% | 7 | 12·5% |
| Congenital anomalies | 0 | 0% | 2 | 3·6% |
| metabolic disorders | 2 | 4·3% | 4 | 7·1% |
| Endocrine disorders | 2 | 4·3% | 1 | 1·8% |
| immunodysregulatory disorders | 2 | 4·3% | 0 | 0% |
| Others* | 6 | 13·0% | 7 | 12·5% |

*^ including neurodevelopmental disorder, neurodegenerative disorder, epilepsy, neurotransmitter disease, movement disorder and neurological immune disorder.*

********conditions associated with other systems or those cannot be classified to any disease categories*

**Supplementary table 5: Number of Outliers Before and After Filtering for OMIM genes for 11 Hypothesis-free cases**

| **Case no.** | **Before Filtering for OMIM genes** | | **After Filtering for OMIM genes** | |
| --- | --- | --- | --- | --- |
|  | **No. of aberrant expression outliers** | **No. of aberrant splicing outliers** | **No. of aberrant expression outliers** | **No. of aberrant splicing outliers** |
| **BF4** | 3 | 7 | 1 | 1 |
| **B47** | 2 | 9 | 0 | 1 |
| **B84** | 13 | 32 | 5 | 7 |
| **B105** | 5 | 6 | 1 | 1 |
| **B107** | 0 | 5 | 0 | 2 |
| **B22** | 6 | 14 | 1 | 4 |
| **B93** | 5 | 16 | 1 | 2 |
| **B114** | 35 | 11 | 7 | 5 |
| **AVERAGE** | 8·6 | 12·5 | 2 | 2·9 |

*B31, B66 and B67 have not been included here owing to their association with RNA signatures, which would require a different analytical workflow during manual inspection as opposed to searching to candidate genes among the outliers.*

**Supplementary table 6: Comparisons between predictions and RNA-seq results in expression and splicing for 9 cases with aberrant splicing events**

| **Case no.** | **Variant** | **NMD prediction (based on the rule)** | **NMD based on RNA-seq results** | **Matched/**  **partially matched/ unmatched for NMD results** | **SpliceAI score**^3^ **and prediction** | **Splice event based on RNA-seq results** | **RNA-seq refined/validated SpliceAI prediction** |
| --- | --- | --- | --- | --- | --- | --- | --- |
| B10^1^ | NM_000116.5 (*TAFAZZIN)*:  c.284+5G>A | PTC created at the start of intron 3 (out of 11 coding exons)  Predicted to undergo NMD | ***No clear indication of NMD (insignificant AE; FDR =1)***  Fold change =0·91  P-value=0·45 | Unmatched | Donor loss = 0·81 (-5bp)  Donor Gain = 0·29 (101 bp)  106bp exon elongation | Two aberrant transcripts detected  1) Major transcript: 106bp exon elongation  2) Minor transcript: 87bp exon elongation | RNA-seq provided more refined information |
| B11^1^ | NM_006767.4  *(LZTR1*)*:*  c.1261-3C>G | PTC within intron 11 and exon 12 (out of 21 coding exons)  Predicted to undergo NMD | ***Only a certain degree of NMD (insignificant AE; FDR=1)***  Fold change=0·75  P-value=0·0018 | Partially matched (possible incomplete NMD) | Acceptor loss= 0·36 (3bp)  Acceptor Gain=0·62(-19bp)  22bp exon elongation | Two aberrant transcripts detected  1) 22bp exon elongation  2) intron retention | RNA-seq provided more refined information |
|  | NM_006767.4  *(LZTR1*)*:*  c.1943-256C>T | PTC created in exon 17 (out of 21 coding exons)  Predicted to undergo NMD (validated through cDNA analysis)^9^ |  |  | Acceptor gain= 0·34 (-122bp)  Donor gain=0·24 (-6bp)  cryptic exon 117bp | Cryptic Exon 117bp | Complete match with SpliceAI |
| B29 | NM_000314.8  *(PTEN):*  c.209+3A>T | In-frame deletion  Predicted to escape NMD | ***No clear indication of NMD (insignificant AE; FDR=1)***  Fold change= 0·97  P-value= 0·83 | Matched | Acceptor loss= 0·91 (-47bp)  Donor loss=0·97 (-3bp)  Skipping of exon 3 | Skipping of exon 3 | Complete match with SpliceAI |
| B47 | NM_017635.5 *(KMT5B):*  c.977+2T>A | PTC in intron 9 right after coding exon 8 (out of 10 coding exons)  Predicted to undergo NMD | ***No clear indication of NMD (insignificant AE; FDR=1)***  Fold change= 0·99  P-value=0·89 | Unmatched | Donor loss= 1·00 (2bp)  Donor gain= 0·96 (-3bp)  Exon elongation of 5bp | Exon elongation of 5 bp | Complete match with SpliceAI |
| B56 | NM_020320.5  *(RARS2*)*:*  c.1238-28T>G | PTC in intron 14 (out of 20 coding exons)  Predicted to undergo NMD | ***Only a certain degree of NMD (insignificant AE; FDR=1)***  Fold change= 0·77  P-value= 0·0045 | Partially matched (possible incomplete NMD) | Acceptor gain= 0·58 (62bp)  90bp exon elongation | Two aberrant transcripts (with two cryptic splice acceptors in intron 14 activated) in similar proportions    1) 74bp exon elongation  2) 90bp exon elongation    Intron retention has also been detected | RNA-seq provided more refined information |
|  | NM_020320.5 (*RARS2*):  c.685C>T p.(Arg229*) | Nonsense variant in exon 9 (out of 20 coding exons)  Predicted to undergo NMD |  |  | NA | NA | NA |
| B68 | NM_078629.4  *(MSL3*)*:*  c.749+5G>A | PTC in intron 7 and exon 8 (out of 13 coding exons)  Predicted to undergo NMD | ***Only a certain degree of NMD (insignificant AE; FDR=1)***  Fold change= 0·81  P-value= 0·027 | Partially matched (possible incomplete NMD) | Donor loss=0·86 (-5bp)  Donor gain=0·71 (64bp)  69bp exon elongation | Three aberrant transcripts  1) 69bp exon elongation of exon 7  2) skipping of exon 7  3) intron retention of intron 7 | RNA-seq provided more refined information |
| B105 | Heterozygous *NF1* deletion:  NC_000017.11:  g.31327741_  31329914delinsT | PTC in exon 39 (out of 58 coding exons)  Predicted to undergo NMD | ***Only a certain degree of NMD (insignificant AE; FDR=1)***  Fold change = 0·81  P-value= 0·023 | Partially matched (possible incomplete NMD) | NA | exon 38 skipping | NA |
| B107 | NM_001042492.3  *(NF1*)*:*  c.2410-16A>G | PTC in intron 20 (out of 58 coding exons)  Predicted to undergo NMD | ***No clear indication of NMD (insignificant AE; FDR=1)***  Fold change= 0·89  P-value=0·20 | Unmatched | Acceptor loss=0·6 (16bp)  Acceptor gain=0·96 (1bp)  Exon elongation of 15bp | Exon elongation of 15 bp | Complete match with SpliceAI |
| B119 | NM_133433.4  *(NIPBL*)*:*  c.64+5G>C | NA (initiation codon affected) | NA | NA | Acceptor loss=0·87(-147bp)  Donor loss=0·94 (-5bp)  exon 2 skipping | exon 2 skipping | Complete match with SpliceAI |

*No clear indication –RNA-seq showed no indication; only a certain degree of nonsense-mediated decay (NMD) – RNA-seq showing a slight decrease in fold change but insignificant statistical result. AE: Aberrant Expression; cDNA: complementary DNA; FDR: false discovery rate; NA: not applicable; NMD: nonsense-mediated decay; PTC: premature termination codon; RNA-seq: RNA sequencing*

**References**

1 Kwok SY, Kwong AKY, Shi JZ, Shih CFY, Lee M, Mak CCY, *et al.* Whole genome sequencing in paediatric channelopathy and cardiomyopathy. *Front Cardiovasc Med* 2024; **11**: 1335527. DOI:10.3389/fcvm.2024.1335527.

2 Barth PG, Scholte HR, Berden JA, Moorsel JMVDK-V, Luyt-Houwen IEM, Veer-Korthof EThV, *et al.* An X-linked mitochondrial disease affecting cardiac muscle, skeletal muscle and neutrophil leucocytes. *J Neurol Sci* 1983; **62**: 327–55. DOI:10.1016/0022-510x(83)90209-5.

3 Jaganathan K, Panagiotopoulou SK, McRae JF, Darbandi SF, Knowles D, Li YI, *et al.* Predicting Splicing from Primary Sequence with Deep Learning. *Cell* 2019; **176**: 535-548.e24. DOI:10.1016/j.cell.2018.12.015.

4 Richards S, Aziz N, Bale S, Bick D, Das S, Gastier-Foster J, *et al.* Standards and guidelines for the interpretation of sequence variants: a joint consensus recommendation of the American College of Medical Genetics and Genomics and the Association for Molecular Pathology. *Genet Med* 2015; **17**: 405–23. DOI:10.1038/gim.2015.30.

5 Walker LC, Hoya M de la, Wiggins GAR, Lindy A, Vincent LM, Parsons MT, *et al.* Using the ACMG/AMP framework to capture evidence related to predicted and observed impact on splicing: Recommendations from the ClinGen SVI Splicing Subgroup. *Am J Hum Genet* 2023; **110**: 1046–67. DOI:10.1016/j.ajhg.2023.06.002.

6 Tayoun ANA, Pesaran T, DiStefano MT, Oza A, Rehm HL, Biesecker LG, *et al.* Recommendations for interpreting the loss of function PVS1 ACMG/AMP variant criterion. *Hum Mutat* 2018; **39**: 1517–24. DOI:10.1002/humu.23626.

7 Johnston JJ, Smagt JJ van der, Rosenfeld JA, Pagnamenta AT, Alswaid A, Baker EH, *et al.* Autosomal recessive Noonan syndrome associated with biallelic LZTR1 variants. *Genet Med* 2018; **20**: 1175–85. DOI:10.1038/gim.2017.249.

8 Chen S, Francioli LC, Goodrich JK, Collins RL, Kanai M, Wang Q, *et al.* A genomic mutational constraint map using variation in 76,156 human genomes. *Nature* 2024; **625**: 92–100. DOI:10.1038/s41586-023-06045-0.

9 Hanses U, Kleinsorge M, Roos L, Yigit G, Li Y, Barbarics B, *et al.* Intronic CRISPR Repair in a Preclinical Model of Noonan Syndrome–Associated Cardiomyopathy. *Circulation* 2020; **142**: 1059–76. DOI:10.1161/circulationaha.119.044794.

10 Kato K, Mizuno S, Inaba M, Fukumura S, Kurahashi N, Maruyama K, *et al.* Distinctive facies, macrocephaly, and developmental delay are signs of a PTEN mutation in childhood. *Brain Dev* 2018; **40**: 678–84. DOI:10.1016/j.braindev.2018.04.008.

11 Martín-Valbuena J, Gestoso-Uzal N, Justel-Rodríguez M, Isidoro-García M, Marcos-Vadillo E, Lorenzo-Hernández SM, *et al.* PTEN hamartoma tumor syndrome: Clinical and genetic characterization in pediatric patients. *Child’s Nerv Syst* 2024; **40**: 1689–97. DOI:10.1007/s00381-024-06301-2.

12 Agrawal S, Pilarski R, Eng C. Different splicing defects lead to differential effects downstream of the lipid and protein phosphatase activities of PTEN. *Hum Mol Genet* 2005; **14**: 2459–68. DOI:10.1093/hmg/ddi246.

13 Dijk T van, Baas F, Barth PG, Poll-The BT. What’s new in pontocerebellar hypoplasia? An update on genes and subtypes. *Orphanet J Rare Dis* 2018; **13**: 92. DOI:10.1186/s13023-018-0826-2.

14 Zhang Y, Yu Y, Zhao X, Xu Y, Chen L, Li N, *et al.* Study of Novel RARS2 Variations Updating Awareness of Diagnosis and Pathogenesis of Pontocerebellar Hypoplasia Type 6. *Pediatr Neurol* 2022; **131**: 30–41. DOI:10.1016/j.pediatrneurol.2022.04.002.

15 Basilicata MF, Bruel A-L, Semplicio G, Valsecchi CIK, Aktaş T, Duffourd Y, *et al.* De novo mutations in MSL3 cause an X-linked syndrome marked by impaired histone H4 lysine 16 acetylation. *Nat Genet* 2018; **50**: 1442–51. DOI:10.1038/s41588-018-0220-y.

16 Brunet T, McWalter K, Mayerhanser K, Anbouba GM, Armstrong-Javors A, Bader I, *et al.* Defining the genotypic and phenotypic spectrum of X-linked MSL3-related disorder. *Genet Med* 2021; **23**: 384–95. DOI:10.1038/s41436-020-00993-y.

17 Cornelia de Lange Syndrome - GeneReviews® - NCBI Bookshelf. <https://www.ncbi.nlm.nih.gov/books/NBK1104/> (accessed Nov 10, 2025).

18 Zhu-Tokita-Takenouchi-Kim Syndrome - GeneReviews® - NCBI Bookshelf. <https://www.ncbi.nlm.nih.gov/books/NBK618356/> (accessed Nov 11, 2025).

19 Aicher JK, Jewell P, Vaquero-Garcia J, Barash Y, Bhoj EJ. Mapping RNA splicing variations in clinically accessible and nonaccessible tissues to facilitate Mendelian disease diagnosis using RNA-seq. *Genet Med* 2020; **22**: 1181–90. DOI:10.1038/s41436-020-0780-y.

20 Vandewalle J, Esch HV, Govaerts K, Verbeeck J, Zweier C, Madrigal I, *et al.* Dosage-Dependent Severity of the Phenotype in Patients with Mental Retardation Due to a Recurrent Copy-Number Gain at Xq28 Mediated by an Unusual Recombination. *Am J Hum Genet* 2009; **85**: 809–22. DOI:10.1016/j.ajhg.2009.10.019.

21 Nicolas-Martinez EC, Robinson O, Pflueger C, Gardner A, Corbett MA, Ritchie T, *et al.* RNA variant assessment using transactivation and transdifferentiation. *Am J Hum Genet* 2024; **111**: 1673–99. DOI:10.1016/j.ajhg.2024.06.018.

22 Herdy J, Schafer S, Kim Y, Ansari Z, Zangwill D, Ku M, *et al.* Chemical modulation of transcriptionally enriched signaling pathways to optimize the conversion of fibroblasts into neurons. *eLife* 2019; **8**: e41356. DOI:10.7554/elife.41356.

23 Li S, Zhao S, Sinson JC, Bajic A, Rosenfeld JA, Neeley MB, *et al.* The clinical utility and diagnostic implementation of human subject cell transdifferentiation followed by RNA sequencing. *Am J Hum Genet* 2024; **111**: 841–62. DOI:10.1016/j.ajhg.2024.03.007.

24 Wiedemann-Steiner Syndrome - GeneReviews® - NCBI Bookshelf. <https://www.ncbi.nlm.nih.gov/books/NBK580718/> (accessed Nov 11, 2025).

25 Molina‐Berenguer M, Vila‐Julià F, Pérez‐Ramos S, Salcedo‐Allende MT, Cámara Y, Torres‐Torronteras J, *et al.* Dysfunctional mitochondrial translation and combined oxidative phosphorylation deficiency in a mouse model of hepatoencephalopathy due to Gfm1 mutations. *FASEB J* 2022; **36**: e22091. DOI:10.1096/fj.202100819rrr.

26 Fishilevich S, Nudel R, Rappaport N, Hadar R, Plaschkes I, Stein TI, *et al.* GeneHancer: genome-wide integration of enhancers and target genes in GeneCards. *Database* 2017; **2017**: bax028. DOI:10.1093/database/bax028.

27 Abascal F, Acosta R, Addleman NJ, Adrian J, Afzal V, Ai R, *et al.* Expanded encyclopaedias of DNA elements in the human and mouse genomes. *Nature* 2020; **583**: 699–710. DOI:10.1038/s41586-020-2493-4.

28 Chui MM-C, Kwong AK-Y, Leung HYC, Pang C, Scheller IF, Wong SS-N, *et al.* An outlier approach: advancing diagnosis of neurological diseases through integrating proteomics into multi-omics guided exome reanalysis. *npj Genom Med* 2025; **10**: 36. DOI:10.1038/s41525-025-00493-5.

29 Chen G, Han L, Tan S, Jia X, Wu H, Quan Y, *et al.* Loss-of-function of KMT5B leads to neurodevelopmental disorder and impairs neuronal development and neurogenesis. *J Genet Genom* 2022; **49**: 881–90. DOI:10.1016/j.jgg.2022.03.004.

30 Aref-Eshghi E, Kerkhof J, Pedro VP, France GD, Barat-Houari M, Ruiz-Pallares N, *et al.* Evaluation of DNA Methylation Episignatures for Diagnosis and Phenotype Correlations in 42 Mendelian Neurodevelopmental Disorders. *Am J Hum Genet* 2020; **106**: 356–70. DOI:10.1016/j.ajhg.2020.01.019.

31 CTCF-Related Disorder - GeneReviews® - NCBI Bookshelf. <https://www.ncbi.nlm.nih.gov/books/NBK603087/> (accessed Nov 12, 2025).

32 Konrad EDH, Nardini N, Caliebe A, Nagel I, Young D, Horvath G, *et al.* CTCF variants in 39 individuals with a variable neurodevelopmental disorder broaden the mutational and clinical spectrum. *Genet Med* 2019; **21**: 2723–33. DOI:10.1038/s41436-019-0585-z.

33 Delgado-Olguín P, Brand-Arzamendi K, Scott IC, Jungblut B, Stainier DY, Bruneau BG, *et al.* CTCF Promotes Muscle Differentiation by Modulating the Activity of Myogenic Regulatory Factors*. *J Biol Chem* 2011; **286**: 12483–94. DOI:10.1074/jbc.m110.164574.

34 Riggs ER, Andersen EF, Cherry AM, Kantarci S, Kearney H, Patel A, *et al.* Technical standards for the interpretation and reporting of constitutional copy-number variants: a joint consensus recommendation of the American College of Medical Genetics and Genomics (ACMG) and the Clinical Genome Resource (ClinGen). *Genet Med* 2020; **22**: 245–57. DOI:10.1038/s41436-019-0686-8.

35 Liu K, Jones S, Minis A, Rodriguez J, Molina H, Steller H. PI31 Is an Adaptor Protein for Proteasome Transport in Axons and Required for Synaptic Development. *Dev Cell* 2019; **50**: 509-524.e10. DOI:10.1016/j.devcel.2019.06.009.

36 Magrinelli F, Tesson C, Angelova PR, Rodriguez JA, Scardamaglia A, O’Callaghan B, *et al.* Variants in the proteasome regulator PSMF1 cause a phenotypic spectrum from parkinsonism to perinatal lethality. *Nat Commun* (in press) 2026. DOI:10.1038/s41467-026-71351-w

37 Chi B, Wang Q, Wu G, Tan M, Wang L, Shi M, *et al.* Aly and THO are required for assembly of the human TREX complex and association of TREX components with the spliced mRNA. *Nucleic Acids Res* 2013; **41**: 1294–306. DOI:10.1093/nar/gks1188.

38 Shen H-H. UAP56- a key player with surprisingly diverse roles in pre-mRNA splicing and nuclear export. *BMB Rep* 2009; **42**: 185–8. DOI:10.5483/bmbrep.2009.42.4.185.

39 Booth KTA, Jangam SV, Chui MMC, Treat K, Graziani L, Soldano A, *et al.* De novo and inherited variants in DDX39B cause a novel neurodevelopmental syndrome. *Brain* 2025; **148**: 2658–70. DOI:10.1093/brain/awaf035.

40 Faqeih EA, Alghamdi MA, Almahroos MA, Alharby E, Almuntashri M, Alshangiti AM, *et al.* Biallelic variants in HECT E3 paralogs, HECTD4 and UBE3C, encoding ubiquitin ligases cause neurodevelopmental disorders that overlap with Angelman syndrome. *Genet Med* 2023; **25**: 100323. DOI:10.1016/j.gim.2022.10.006.

41 Castel AL, Cleary JD, Pearson CE. Repeat instability as the basis for human diseases and as a potential target for therapy. *Nat Rev Mol Cell Biol* 2010; **11**: 165–70. DOI:10.1038/nrm2854.

42 Abrahams BS, Arking DE, Campbell DB, Mefford HC, Morrow EM, Weiss LA, *et al.* SFARI Gene 2.0: a community-driven knowledgebase for the autism spectrum disorders (ASDs). *Mol Autism* 2013; **4**: 36. DOI:10.1186/2040-2392-4-36.

43 Rubeis SD, He X, Goldberg AP, Poultney CS, Samocha K, Cicek AE, *et al.* Synaptic, transcriptional and chromatin genes disrupted in autism. *Nature* 2014; **515**: 209–15. DOI:10.1038/nature13772.

44 Yuen RKC, Merico D, Bookman M, Howe JL, Thiruvahindrapuram B, Patel RV, *et al.* Whole genome sequencing resource identifies 18 new candidate genes for autism spectrum disorder. *Nat Neurosci* 2017; **20**: 602–11. DOI:10.1038/nn.4524.

45 Haag N, Tan EC, Begemann M, Buschmann L, Kraft F, Holschbach P, *et al.* Biallelic loss-of-function variants in WDR11 are associated with microcephaly and intellectual disability. *Eur J Hum Genet* 2021; **29**: 1663–8. DOI:10.1038/s41431-021-00943-5.

46 Kim HG, Ahn JW, Kurth I, Ullmann R, Kim HT, Kulharya A, *et al.* WDR11, a WD protein that interacts with transcription factor EMX1, is mutated in idiopathic hypogonadotropic hypogonadism and Kallmann syndrome. *Am J Hum Genet* 2010; **87**: 465–79. DOI:10.1016/j.ajhg.2010.08.018.

47 Globa E, Zelinska N, Shcherbak Y, Bignon-Topalovic J, Bashamboo A, MсElreavey K. Disorders of Sex Development in a Large Ukrainian Cohort: Clinical Diversity and Genetic Findings. *Front Endocrinol* 2022; **13**: 810782. DOI:10.3389/fendo.2022.810782.

48 Castro S, Brunello FG, Sansó G, Scaglia P, Azcoiti ME, Izquierdo A, *et al.* Delayed Puberty Due to a WDR11 Truncation at Its N-Terminal Domain Leading to a Mild Form of Ciliopathy Presenting With Dissociated Central Hypogonadism: Case Report. *Front Pediatr* 2022; **10**: 887658. DOI:10.3389/fped.2022.887658.

49 Uggenti C, Lepelley A, Depp M, Badrock AP, Rodero MP, El-Daher MT, *et al.* cGAS-mediated induction of type I interferon due to inborn errors of histone pre-mRNA processing. *Nat Genet* 2020; **52**: 1364–72. DOI:10.1038/s41588-020-00737-3.

50 Aicardi-Goutières Syndrome - GeneReviews® - NCBI Bookshelf. <https://www.ncbi.nlm.nih.gov/books/NBK1475/> (accessed Nov 13, 2025).
